# Flow matching for reaction pathway generation

**DOI:** 10.1038/s41467-026-75654-w

**Published:** 2026-07-17

**Authors:** Ping Tuo, Jiale Chen, Ju Li

**Affiliations:** 1 Bakar Institute of Digital Materials for the Planet, University of California, Berkeley, Berkeley, CA USA; 2 Institute of Science and Technology Austria, Klosterneuburg, Austria; 3 Department of Nuclear Science and Engineering, Massachusetts Institute of Technology, Cambridge, MA USA; 4 Department of Materials Science and Engineering, Massachusetts Institute of Technology, Cambridge, MA USA

**Keywords:** Reaction kinetics and dynamics, Computational science

## Abstract

Elucidating reaction mechanisms requires efficient generation of transition states (TSs) and products. Existing diffusion and sequence-based models accelerate parts of this process over traditional string-based methods, but typically still require manual enumeration of either TSs or products, and stochastic diffusion dynamics can be inefficient and hard to control. We introduce MolGEN, a conditional flow-matching framework that uses deterministic optimal transport to map Gaussian priors to chemical distributions. For TS generation, MolGEN improves TS geometry and barrier-height prediction over diffusion models while enabling sub-second sampling. For reaction product generation, it achieves competitive top-*k* accuracy while preserving mass and electron balance. Using the same backbone for TS and product sampling, MolGEN enables template-free generative exploration of reaction networks without the repeated quantum-chemistry searches required by prior methods. For the *γ*-ketohydroperoxide decomposition network, it produces more valid TSs than string-based methods using only 12 quantum-chemistry evaluations instead of 1156, and identifies a lower-barrier pathway.

## Abstract (web summary)

The study introduces MolGEN, an optimal transport flow-matching framework that generates reaction products and transition states, enabling faster reaction-pathway discovery and identifying lower-energy routes.

## Introduction

Predicting the outcomes of chemical reactions from given reactants and conditions remains a central yet formidable challenge in chemistry. Expert chemists typically rely on mechanistic reasoning, often visualized through arrow-pushing diagrams, to anticipate reaction pathways. However, identifying plausible mechanisms purely through intuition is frequently insufficient, motivating the development of computational and quantitative approaches for mechanistic exploration.

There have been several classes of automated methods for generating reaction networks from specified reactants using quantum chemical calculations^1^. Template-based approaches encode known elementary reaction rules to construct networks of potential intermediates and products^2^. Physics-based methods identify transition states (TSs) to assess kinetic feasibility, followed by intrinsic reaction coordinate analyses to trace reaction pathways^3–9^. Graph-based algorithms represent molecules as graphs and recursively enumerate intermediates accessible via single-step transformations^10,11^, subsequently linking reactants and products through techniques such as the growing string and nudged elastic band methods. While these strategies provide powerful frameworks for elucidating mechanisms and discovering novel pathways, they remain computationally demanding. However, constructing even a moderately sized reaction network can require thousands of elementary steps, demanding millions of DFT single-point evaluations^12^. This computational bottleneck has spurred the development of machine learning approaches capable of modeling complex chemical transformations with greater efficiency.

Diffusion models have emerged as state-of-the-art approaches for molecular generation, wherein a neural network learns the underlying probability distribution and generates new configurations by modeling a reverse-time stochastic diffusion process. This process gradually transforms a simple prior distribution (e.g., Gaussian noise) into a complex data distribution. Numerous diffusion frameworks have been developed for central problems in physics and chemistry^13–15^. In chemistry, diffusion-based approaches such as TSDiff^16^ and OA-ReactDiff^17^ have been introduced to generate the transition states of each elementary reaction in a reaction network, replacing the costly nudged elastic band or string methods. However, their reliance on stochastic differential equations (SDEs) introduces limitations familiar to all diffusion approaches: stochastic noise complicates training, slows sampling, and conditional sampling and guidance of diffusion models require complex, task-specific guidance schemes and backpropagation through long stochastic trajectories^18^.

Flow matching offers a deterministic alternative. Instead of learning a stochastic reverse diffusion process, a flow-matching model learns the velocity field of an ordinary differential equation (ODE) that continuously transports a prior distribution to a target distribution. This replaces the noisy SDE formulation of diffusion models with a smooth, analytically defined flow, yielding stable training, precise trajectory control, and dramatically faster sampling. Conceptually, flow matching unifies the family of SDE-based diffusion models, rectified flows^19^, and consistency models^20^ under a single deterministic transport framework.

Here, we show that flow matching can fully replace diffusion-based formulations for reaction mechanism generation. We introduce MolGEN, a conditional flow-matching framework that generates both TSs and reaction products and, crucially, rolls out multi-step reaction networks using generative inference alone, without templates or hand-crafted rules. To our knowledge, MolGEN is the first end-to-end reaction-network generation framework powered solely by generative models for both TS and product sampling, enabling unified, automated exploration from reactants to complete networks. In generating transition states, we show that MolGEN halves the TS geometry prediction error of the state-of-the-art diffusion models while reducing sampling time to sub-second inference. Remarkably, it also uncovers alternative transition states with lower barrier heights than those reported in the reference data, underscoring its potential to reveal previously inaccessible mechanistic pathways. In generating reaction products, we evaluate MolGEN on reaction product generation from given reactants, where it attains top-*k* accuracies comparable to state-of-the-art sequence-based approaches while naturally respecting stoichiometric constraints. Together, these results show that a deterministic flow formulation provides an integrated solution for reaction-network rollout. In an integrated generative modeling solution for reaction network exploration, we apply this framework to the decomposition network of *γ*-ketohydroperoxide (KHP). In this realistic benchmark, MolGEN achieves substantial computational efficiency gains over conventional string-based methods while maintaining high mechanistic fidelity, and it identifies a new decomposition pathway with a barrier lower than the reported minimum. Overall, the combination of high-quality generation, minimal computational cost, and unified TS and product sampling positions flow matching as a compelling paradigm for automated generative reaction network generation.

## Results

### A conditional flow matching design for reaction pathway generation

A flow matching model learns a transformation from an initial distribution, typically a standard Gaussian, to a target distribution. This transformation is defined by an optimal transport path connecting the source and target distributions. A neural network is trained to approximate the corresponding velocity field that governs the evolution of the interpolant along this path.

**Fig. 1:**
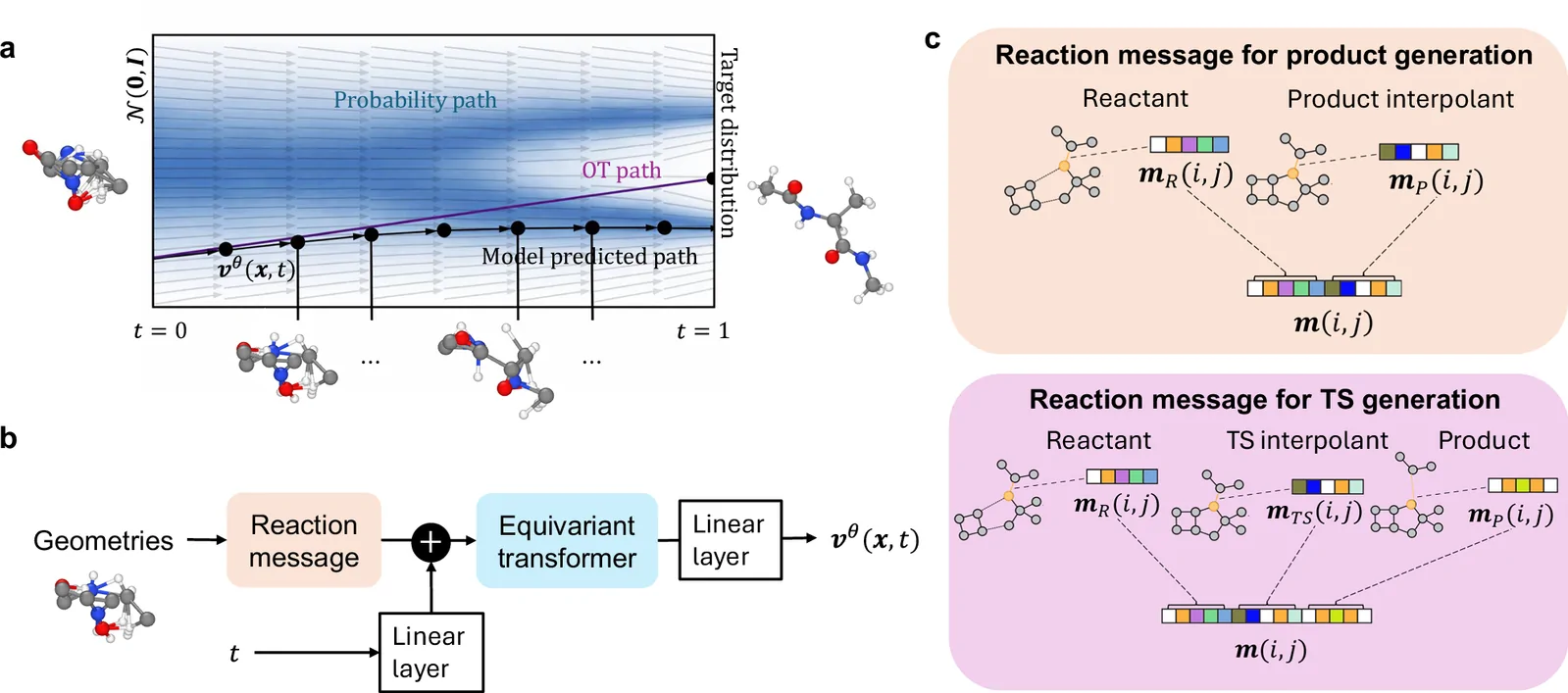
Overview of MolGEN. **a** Schematic illustration of the flow-matching workflow. Given the reactant geometry (or both reactant and product geometries), MolGEN starts from a randomly initialized product or transition-state (TS) geometry. A flow network predicts a velocity field that is iteratively integrated to transform this random geometry into the target product or TS. The model is trained using a flow-matching (FM) loss, which matches the target velocity field of the optimal-transport (OT) path, together with a Jensen-Shannon (JS) loss, which matches the probability path indicated by the blue shading. **b** Schematic of the message-passing neural network (MPNN) flow model. Input geometries, such as the reactant and the product interpolant, are encoded into reaction messages using Clebsch-Gordan contractions of SE(3)-equivariant tensor features constructed from spherical harmonics and radial basis functions^42^. In parallel, the scalar integration time *t* is passed through a linear layer to generate a time embedding. This embedding is added to the reaction messages, which are then processed by an SE(3)-equivariant transformer^43^. A final linear layer outputs the flow **v**_*t*_, which is used to update the interpolant during integration. **c** Illustration of the reaction messages. Here, **m**(*i*, *j*) denotes the pairwise reaction message associated with atoms *i* and *j*, which encodes the geometric and chemical information used for message passing between the two atoms. For product generation, the reactant message **m**_R_(*i*, *j*) is computed from the reactant geometry, while the product message **m**_P_(*i*, *j*) is computed from the flow-matching interpolant. The full reaction message **m**(*i*, *j*) is obtained by concatenating **m**_R_(*i*, *j*) and **m**_**P**_(*i*, *j*). For TS generation, the reaction message is constructed analogously by concatenating messages from the reactant geometry, product geometry, and TS interpolant, denoted by **m**_R_(*i*, *j*), **m**_P_(*i*, *j*), and **m**_TS_(*i*, *j*), respectively.

MolGEN extends this framework by introducing a conditioning mechanism that steers the generation process toward specific target distributions. MolGEN employs a message passing neural network (MPNN) architecture. For the task of TS generation, we modify the MPNN by concatenating the messages of the transition state (TS) interpolant and the reaction-product pair to form a reaction message, as illustrated in Fig. 1. This process integrates the bond information of all three states into the reaction messages. Analogously, for the task of reaction product generation, we concatenate the messages of the reactant and the product interpolant to form the reaction message. Subsequently, the model passes these reaction messages through message passing layers to predict the velocity field that evolves the distribution.

MolGEN initiates with a standard Gaussian distribution. Therefore, it also incorporates stochasticity while performing a deterministic forward pass. The probability path simulated by MolGEN resembles that of a diffusion model, in the sense that both follow a transformation from a standard Gaussian distribution to a jagged target distribution. Consequently, MolGEN is capable of exploring the free energy surface as effectively as diffusion-based models.

We train MolGEN by two different loss functions. The first one is the flow matching loss

1 $${{\mathcal{L}}}_{{\rm{FM}}}(\theta )={{\mathbb{E}}}_{t,{p}_{t}({\bf{x}})}\parallel {{\bf{v}}}_{t}^{\theta }({\bf{x}})-{{\bf{u}}}_{t}({\bf{x}}){\parallel }_{1},$$

where *θ* denotes the learnable parameters of the velocity field model and *t* ∈ [0, 1]. **x** ~ *p*_*t*_(**x**) denotes the optimal transport path evolving from the initial Gaussian distribution to the target distribution, and **u**_*t*_(**x**) denotes the corresponding velocity field driving the transformation. Details of the optimal transport path, the corresponding velocity field, and the loss functions are given in the Methods. Since MolGEN generates a distribution, we also use a conditional modified Jensen-Shannon (JS) divergence loss to measure the difference of the predicted probability path from the optimal transport probability path (refer to Methods for details):

2 $${{\mathcal{L}}}_{{\rm{JSD}}}(\theta )={{\mathbb{E}}}_{t,{p}_{t}({\bf{x}})}{D}_{{\rm{JS}}}[{p}_{t}({\bf{x}})\parallel {p}_{t}^{\theta }({\bf{x}})].$$

By design, MolGEN engrosses the strength of both diffusion and flow matching paradigms. Firstly, it performs sampling via an optimal transport path, thus enabling fast inference that requires only  ~0.8 s inference time, orders of magnitude faster than the diffusion model. Secondly, it can generate both TS and reaction products with equal diversity and increased accuracy than diffusion models.

### Generating transition states

We first demonstrate that MolGen is effective in generating transition states. We trained MolGEN on Transition1x^21^, a dataset that contains paired reactants, TSs and products calculated from climbing-image nudged elastic band method (NEB)^22^ obtained with DFT (*ω*B97x/6-31G(d))^23,24^. Transition1x contains 11,961 reactions with product-reactant pairs sampled from the GDB-7 dataset^25^. Each reaction consists of up to seven heavy atoms, including C, N, O, and 23 atoms in total. We use the same train-validation-test partitioning as reported with the database^21^. All datasets are stratified by molecular formula such that no two configurations that come from different data splits are constituted of the same atoms. Test and validation data each consist of 5% of the total data and are chosen such that configurations contain all heavy atoms (C, N, O). All results are reported on a single, fixed train/validation/test split of the database, which is kept identical across all compared models.

In the following, we compare the performance of MolGEN with that of diffusion models TSDiff and OA-ReactDiff, as well as a double-ended flow matching model ReactOT^26^. TSDiff is a diffusion model that generates TS geometries from a Gaussian prior via a stochastic denoising trajectory conditioned on 2D reactant/product graphs. OA-ReactDiff is an object-aware diffusion model that jointly generates reactant (R), TS, and product (P) structures from a Gaussian prior. ReactOT instead formulates TS prediction as an optimal-transport / rectified-flow problem, deterministically mapping the midpoint (**x**_R_ + **x**_P_)/2 to a unique **x**_TS_, where **x**_R_, **x**_P_, **x**_TS_ denote the coordinates of R, P, and TS. In contrast, MolGEN is a flow-matching model that generates TS geometries from a Gaussian prior via a deterministic flow conditioned on R and P.

MolGEN generates a stochastic distribution by a deterministic pass, indicating two key characteristics. (1) The generation occurs near stationary points on the potential energy surface, where the energy gradient approaches zero. This contrasts with the double-ended flow matching model, which yields a unique configuration. Considering this, we generate 30 independent samples for each test TS and select the one with the lowest energy as the final prediction. Since these samples can be generated in parallel, this approach does not necessarily increase inference time. (2) MolGEN is also capable of identifying alternative TS configurations, including those with lower transition barriers than the reference data, an ability that double-ended flow-matching models lack.

**Fig. 2:**
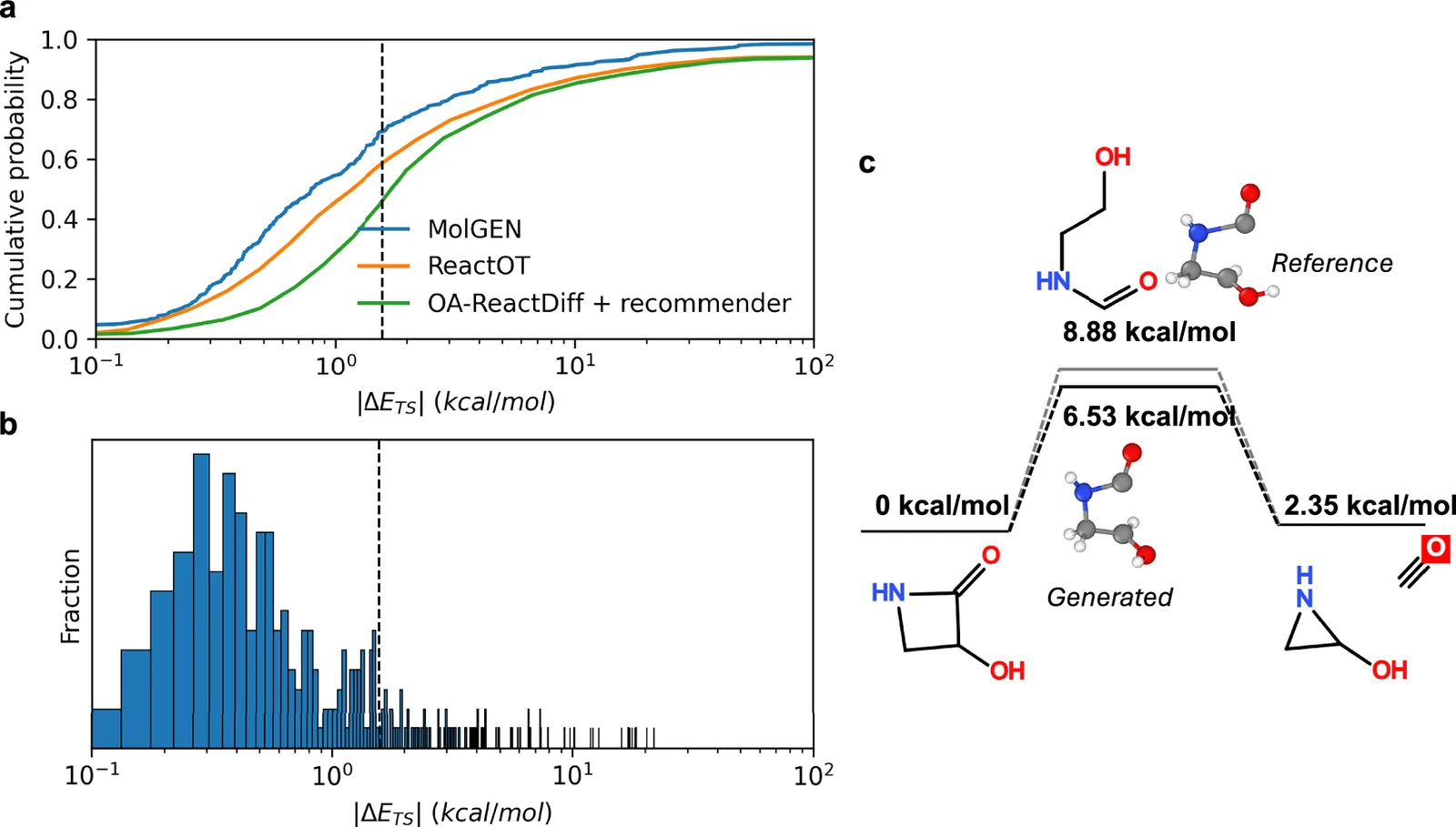
Performance of MolGEN in transition state generation. **a** Cumulative probability of the barrier height error. The ReactOT and OA-ReactDiff results are reproduced from Reference^26^ and^17^, respectively. The dashed line marks the threshold of chemical accuracy. **b** Histogram of the barrier height error. **c** An example transition state (TS) generated by MolGEN, with a lower barrier than the reference. Source data are provided as a Source Data file.

The performances of MolGEN are compared with some state-of-the-art models in Table 1. MolGEN achieves a mean root mean square deviation (RMSD) of approximately 0.106 Å between the generated and reference TS structures on the Transition1x test set, halving the error of diffusion models and matching the error of the double-ended flow matching model^26^. The mean barrier height error produced by MolGEN is about 3.285 kcal/mol, also halving that of diffusion models and lower than that of the double-ended flow matching model (3.608 kcal/mol). The barrier height error distribution of MolGEN, OA-ReactDiff and ReactOT is compared in Fig. 2a and b. In chemical reactions, a difference of one order of magnitude in reaction rate is often used as the characterization of chemical accuracy, which translates to 1.58 kcal/mol in barrier height error assuming a reaction temperature of 70 °C (further explained in “Methods”). By this standard, over 54% of TS from MolGEN have high chemical accuracy relative to the reference data, on par with that of the state-of-the-art of the double-ended flow matching model (57%).

**Table 1:** Summary of statistics for root mean squared distance (RMSD), barrier height error ∣Δ*E*∣, ratio of valid transition states (TS) and ratio of updated TS, where the generated TS have lower energies than the corresponding reference TS by more than 0.1 kcal/mol

Training and testing database	Model	Loss function	mean RMSD (Å)	mean∣Δ*E*∣(kcal/mol)	Chem. accu. (%)	Ratio of valid TS (%)	Ratio of updated TS (%)
Transition1x^a^Grambow^b^	TSDiff	JS div	0.253^a^	10.369^a^	10.9^a^	97.00^b^	30.96^b^
Transition1x	OA-ReactDiff+recommender	JS div	0.129^a^	4.453^a^	48.7^a^		
Transition1x	React-OT	FM	0.103^a^	3.337^a^	63.6^a^		
Transition1x	MolGEN	FM & finetune by JS div	0.106	3.285	54.38	87.46	7.32
Transition1x(Pretrained by RGD1-xTB)	React-OT+pretraining	FM	0.088 0.098^a^	3.608 2.864^a^	57.0 71.9^a^	74.67	4.00
RGD1+Transition1x	MolGEN	FM & finetune by JS div	0.100	2.759	70.80	89.20	27.87
RGD1+Transition1x	MolGEN	JS div	0.112	2.844	68.98	88.50	30.66

React-OT was evaluated using the same pretrained model provided by the original authors.

^a^ Values from Duan^26^, trained/tested on Transition1x^21^.

^b^ Values from Kim^16^, trained/tested on the Grambow dataset^48^.

The ratio of chemical accuracy translates to ∣Δ*E*∣ < 1.58 kcal/mol. The loss functions used for training include Jensen-Shannon divergence loss (JS div) and flow matching loss (FM).

MolGEN is capable of identifying alternative TS configurations beyond those present in the reference dataset. To quantify this capability, we performed quantum chemical validation on the predicted configurations, using saddle point optimization. The ratio of valid TS, those with a single imaginary vibration frequency, exceeds 87%, notably higher than the ratio achieving chemical accuracy. Moreover, over 7% of the TS configurations have energies lower than their corresponding reference TS by more than 0.1 kcal/mol. Figure 2c presents a representative example of MolGEN-generated TS configuration.

#### Training on additional reactions further improves MolGEN

The performance of MolGEN can be further enhanced by training on a larger and more diverse dataset. We combined RGD1^27^ with Transition1x for this purpose. RGD1 covers 177,992 reactions involving molecules containing *C*, *H*, *N*, and *O* atoms, with up to 10 heavy atoms. The TSs and products in RGD1 are derived from NEB obtained with DFT (B3LYP-D3/TZVP). Of these, 3% and 6% are held out as a test and validation set, respectively.

Training on this expanded dataset reduced the mean RMSD to 0.100 Å and the mean barrier height error to 2.759 kcal/mol. More notably, the ratio of valid TS increased to 89.20%, while the ratio of TS with energies lower than the reference rose sharply to 27.87%, approaching the performance achieved by the diffusion-based model TSDiff^16^ (30.96%).

Although RGD1 and Transition1x are computed at different levels of DFT, the resulting TS geometries are typically within  ~ 0.05 − 0.1 Å of each other in RMSD for the same reaction^28–30^. This difference is comparable to the current predictive uncertainty of MolGEN (~ 0.1 Å), so the functional mismatch mainly acts as a mild label noise rather than inducing qualitatively different structures. In practice, the additional RGD1 reactions substantially increase the diversity of TS topologies and local bonding patterns seen during training, which appears to outweigh the modest decrease in electronic-structure fidelity, leading to overall improved generalization. This suggests that generative models can be trained on relatively inexpensive DFT data without substantially compromising performance, and that further improvements may be achieved by incorporating higher-quality data.

#### Training on JS divergence improves TS identification

We can drive the model to seek a stationary point even further through training only by JS divergence loss, as evidenced in Table 1. When trained only by JS divergence, the ratio of TS with energies lower than reference rose to 30.66%, matching the performance of diffusion models^16^, even though the mean RMSD increased slightly to 0.112 Å. This indicates that the model is locating more alternative TS with lower energies than the reference data.

#### Analysis on failure cases

We performed a saddle point optimization on the 33 reactions for which the model failed to generate the correct transition state (TS) and verified the validity of the reference TSs by whether they possess a single imaginary frequency. Six of the reference TSs did not pass this saddle point validation. Among the remaining 26 reactions, 23 failed the intrinsic reaction coordinate (IRC) calculation, indicating the presence of a substantial portion of erroneous data in the database, where the transition states do not match with their corresponding reactions.

#### Inference time

Since the ODE follows an optimal transport trajectory, it theoretically requires only two evaluations to reach the same target state. To quantify the deviation of the learned dynamics from this optimal transport path, we systematically reduce the number of function evaluations (nfe) during sampling. Empirically, MolGEN attains convergence at nfe = 10, at which point the mean RMSD of the generated TS stabilizes, corresponding to an inference time of 0.8 s. This stands in stark contrast to diffusion models, as TSDiff requires 5000 nfe for sampling^16^, and OA-ReactDiff requires  ≳ 800 nfe^17^. In practice, we employ the dopri5 adaptive integrator^31^ to guarantee numerical stability and convergence, yielding an average inference time of 2.1 s.

### Generating reaction products

In the TS generation task, MolGEN was used to realize a one-to-one mapping, i.e., from the reactant-product pair to the TS. Here, we demonstrate that MolGEN can also capture open-ended mappings with multiple outcomes by training a separate MolGEN model to generate the product from a given reactant.

MolGEN was trained on a combined dataset of Transition1x and RGD1, comprising elementary reaction data as described in the previous section. Model performance was evaluated on the Transition1x test set.

We assessed predictive performance using top-*k* accuracy, which quantifies the model’s ability to correctly anticipate plausible products for a given reactant. Leveraging the distributional nature of conditional flow matching, MolGEN can be sampled repeatedly for the same reactant to generate a diverse ensemble of candidate products. Each predicted product geometry is converted to an adjacency matrix^32^ and ranked according to its sampling frequency.

The top-*k* step accuracy measures the fraction of individual elementary steps where the recorded product appears within the top-*k* ranked predictions. Multiple valid products can emerge from the same reactants due to differences in reaction conditions (e.g., the use of distinct acids, bases, or catalysts) or alternative resonance structures. Within the Transition1x test set, we identified 37 unique reactants (distinct adjacency matrix), each associated with between one and twenty-seven distinct products, totaling 287 reaction entries.

**Fig. 3:**
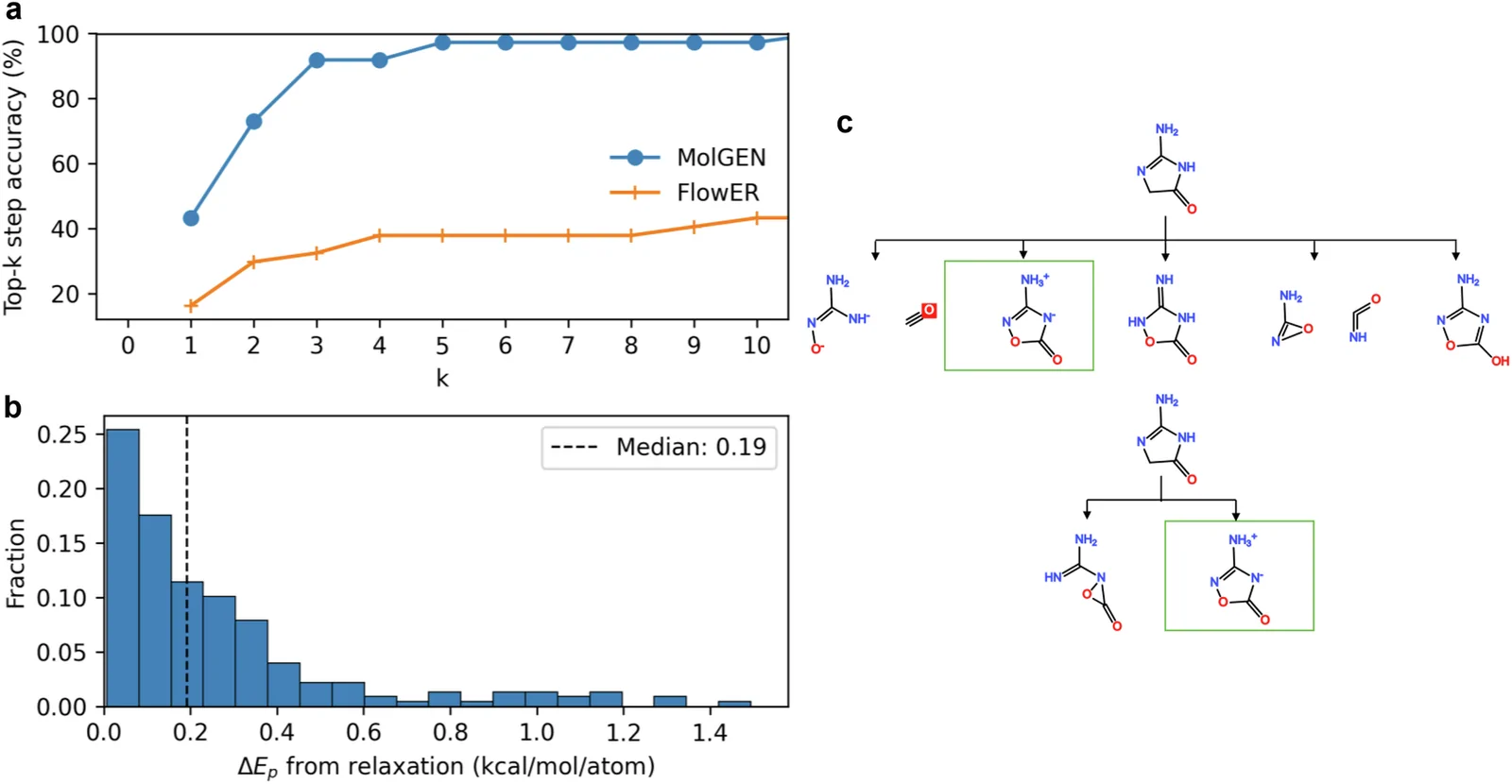
Performance of MolGEN in reaction product generation. **a** Top-*k* step accuracy for product prediction of an elementary reaction. The FlowER result is included as a contextual sequence-model baseline. **b** Energy change of the product (Δ*E*_*P*_) following structural relaxation. **c** A representative product prediction example, with reference reactions shown on top and the corresponding predictions shown below. Source data are provided as a Source Data file.

In Fig. 3a, we compare the top-*k* accuracy of MolGEN with FlowER^33^, a representative sequence-based model with electron-distribution encoding that maps reactant SMILES to product SMILES. Because FlowER and MolGEN differ substantially in representation, preprocessing, and output space, we treat this comparison as a contextual baseline rather than a strictly controlled head-to-head evaluation. On the Transition1x-derived product-generation task considered here, MolGEN achieves near-saturated top-3 accuracy. Importantly, because MolGEN generates products by moving atoms in 3D space while preserving atomic identities, it enforces stoichiometry by construction and avoids mass-balance violations that can occur in unconstrained sequence generation. Figure 3b presents the energy deviation of the predicted geometry from the local minimum, which assesses the absolute accuracy of the predicted coordinates. A representative example of the predicted product and the corresponding reference is shown in Fig. 3c.

To further demonstrate of MolGEN’s ability, in the next section, we combine the product generation model and the TS generation model obtained in the previous section to address the task of reaction network exploration.

### An integrated generative modeling solution for reaction network exploration

By integrating the product generation model with the transition-state (TS) generation model, we build a generative modeling framework that automates reaction-network exploration. A key motivation for developing predictive chemistry models that operate at the mechanistic (elementary step) level is their potential to generalize beyond standard reaction types encountered during training. To evaluate this capability, we apply our method to the decomposition network of *γ*-ketohydroperoxide (KHP), a realistic and chemically significant system whose reactant does not appear in the training dataset.

**Fig. 4:**
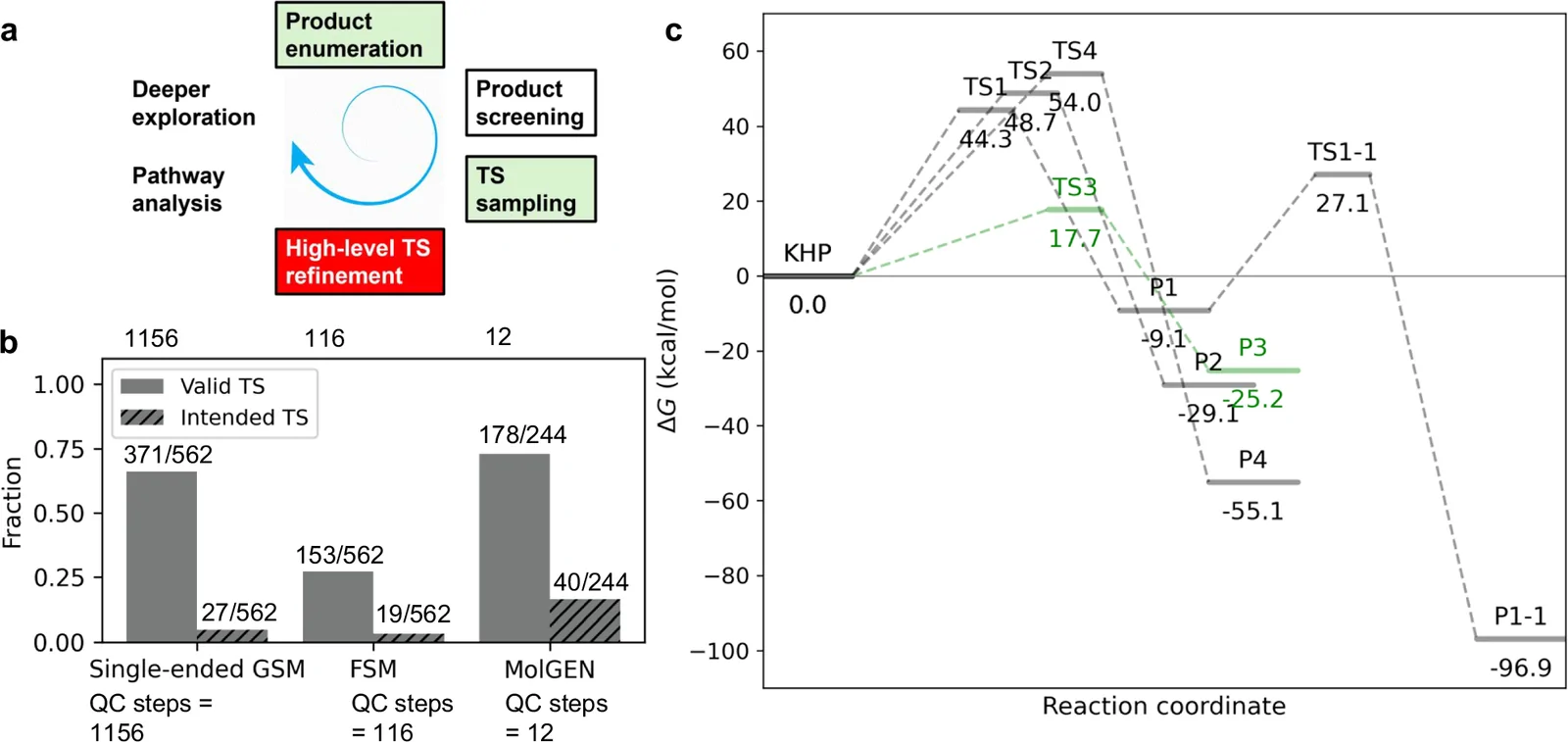
Reaction network exploration results for *γ*-ketohydroperoxide (KHP) decomposition. **a** Workflow of reaction network exploration. Product enumeration and transition states (TS) sampling are each performed by a separate MolGEN model. Product screening is carried out using fixed, physically motivated criteria. **b** Fractions of valid and intended TSs. Bars show fractions, and labels show the corresponding counts. The row below the x-axis reports the total number of high-level quantum chemical (QC) gradient-descent steps used to explore the first-step KHP decomposition reactions. The values for the single-ended growing string method (GSM) and double-ended freezing string method (FSM) are adapted from Grambow^37^. **c** Representative decomposition pathways of KHP. All energies are computed at the *ω*B97X/6-31G(d) level of theory. Highlighted in green is the pathway with the lowest transition barrier, which is lower than the minimum barrier reported by Yet Another Reaction Program (YARP)^35^. Source data are provided as a Source Data file.

KHP decomposition proceeds through multiple mechanistic steps. Figure 4a illustrates a representative workflow for reaction network exploration, comprising five stages: (1) product enumeration, (2) product pruning by physically motivated criteria (refer to Methods for details), (3) TS sampling, (4) high-level TS refinement including IRC calculations to validate that TS geometries correspond to the given reaction, and (5) reaction relevance analysis (pathway analysis). These stages are recursively applied to both the initial reactant(s) and any intermediates generated during exploration. The computational bottleneck lies in the TS refinement stage; consequently, extensive efforts focus on optimizing product and TS sampling and pruning to minimize the number of expensive TS refinements required.

For product generation, traditional reaction network exploration packages such as Yet Another Reaction Program (YARP)^34,35^ employ the graph enumeration to explore potential products based on empirical rules like b2f2 (break 2 bonds and form 2 new bonds). While effective, such rules may overlook feasible products. TS sampling presents an additional challenge. Since reactant-product pairs can be aligned in infinitely many ways, the alignment influences the success rate of subsequent TS refinements. YARP employs heuristic criteria, such as mass-weighted root-mean-square displacement, to identify alignments likely to yield successful TS optimizations via nudged elastic band (NEB) or growing string method (GSM).

In our framework, the product generation model performs stage (1), while the TS generation model handles stage (3). Stage (1): Using KHP as input, the product generation model generated 1000 candidate products, which were grouped into 80 unique products based on adjacency matrices. Geometry optimizations of the lowest-energy configurations identified 65 local minima, as verified by vibrational analysis, six of which shared the same adjacency matrix as the original KHP structure. Stage (3): The remaining 59 unique products (compared with 20 reported by YARP^35^)were used as inputs for the TS generation model. Out of the 59 selected TS guesses, 40 were successfully optimized to valid TS structures characterized by a single imaginary frequency. These TS were subjected to IRC calculations, yielding 14 intended reactions with intended transition states (compared with 13 identified by YARP^35^). Among the products of these intended reactions, three contained radicals, which tend to be endothermic and result from high transition barriers (refer to S3.3), were excluded, leaving the remaining 11 products as reactants for subsequent exploration steps. The generated first-step KHP decomposition network^36^ is benchmarked against YARP in Table 2.

**Table 2:** Comparison of the first step of *γ*-ketohydroperoxide (KHP) decomposition reaction network generated by MolGEN and by YARP^35^

	Num. of unique reactions	Num. of intended unique reactions
YARP	20	13
MolGEN	59	14

The fractions of valid and intended transition-state (TS) structures, along with the total number of gradient descent calls (excluding IRC calculations) required for TS optimization, are summarized in Fig. 4b and compared with results from string-based methods reported by Grambow et al.^37^. Both the valid and intended TS fractions achieved by MolGEN exceed those of the string methods. More importantly, the number of gradient descent calls is drastically reduced to 12 per reaction. This indicates that the MolGEN-based workflow substantially reduces the number of expensive quantum chemical calculations required to assemble a reaction network. Figure 4c presents a decomposition pathway identified in this work (highlighted in green) with a barrier lower than the minimum barrier reported by YARP^35^.

## Discussion

Flow matching can establish a mapping from a simple prior distribution to a complex data distribution by an optimal-transport path. We develop a conditional flow matching framework, MolGEN, capable of addressing both double-ended generation tasks (one-to-one mappings), such as transition-state (TS) generation from known reactant-product pairs, and open-ended generation tasks (multiple-target sampling), such as product generation from a known reactant.

For TS generation, in contrast to diffusion-based models, which rely on computationally expensive stochastic sampling, flow matching enables sub-second inference, offering a significant speed advantage with improved accuracy, and without compromising diversity. For product generation, MolGEN achieves high top-*k* accuracy on the Transition1x-derived product-generation benchmark while preserving stoichiometry by construction. Furthermore, it provides high-quality initial geometries for products, exhibiting a median post-relaxation energy change of only 0.19 kcal/mol per atom, indicating strong alignment between predicted and equilibrium structures.

By integrating the TS generating model and product generating model, MolGEN offers an integrated generative framework for automated reaction network exploration. The fraction of both valid and intended TSs is improved relative to traditional methods, while the optimization cost is drastically reduced. Nonetheless, not all intended TSs corresponding to the predicted products were recovered in our demonstration. This implies that the model requires further refinement, particularly through the use of a higher-quality database, as the current one contains a large proportion of unintended TS.

In summary, MolGEN illustrates that flow matching provides a unified, accurate, and computationally efficient framework for both double-ended and open-ended molecular generation. It provides a unified framework that explores reaction networks using a single generative modeling backbone for both TS and product sampling. Its combination of enhanced accuracy, deterministic inference, and scalability points toward a promising future for flow-matching models as a strong candidate for molecular and reaction generation.

## Methods

### Flow matching by optimal transport path

#### Preliminaries: continuous normalizing flows

Let ${{\mathbb{R}}}^{d}$ denote the data space with points ${\bf{x}}\in {{\mathbb{R}}}^{d}$. Two important variables we use are: the probability density path *p*_*t*_ ∈ [0, 1], which is a time-dependent density, and a time-dependent vector field ${{\bf{v}}}_{t}\in {{\mathbb{R}}}^{d}$. The vector field **v**_*t*_ generates a time-dependent diffeomorphism (or a flow) ${\phi }_{t}:{{\mathbb{R}}}^{d}\to {{\mathbb{R}}}^{d}$ via the ordinary differential equation (ODE):

3 $$\frac{d}{dt}{\phi }_{t}({\bf{x}})={{\bf{v}}}_{t}\left({\phi }_{t}({\bf{x}})\right),$$

4 $${\phi }_{0}({\bf{x}}) \sim {p}_{0}.$$

Chen et al.^38^ suggested modeling **v**_*t*_ by a neural network ${{\bf{v}}}_{t}^{\theta }({\bf{x}})$, where *θ* are learnable parameters, yielding a deep parametrization of the flow *ϕ*_*t*_, called a continuous Normalizing Flow (CNF). A CNF pushes a simple base density *p*_0_ (e.g., Gaussian noise) to a complex target *p*_1_ via

5 $${p}_{t}={[{\phi }_{t}]}_{*}{p}_{0},$$

where the push-forward operator is defined by the change-of-variables formula

6 $${[{\phi }_{t}]}_{*}{p}_{0}({\bf{x}})={p}_{0}\left({\phi }_{t}^{-1}({\bf{x}})\right){{\det }}\left({\partial }_{{\bf{x}}}{\phi }_{t}^{-1}({\bf{x}})\right).$$

A vector field **v**_*t*_ generates a density path *p*_*t*_ if its flow *ϕ*_*t*_ satisfies the above. Equivalently, one may verify the continuity equation

7 $${\partial }_{t}{p}_{t}+\nabla \cdot \left({p}_{t}{{\bf{v}}}_{t}\right)=0.$$

#### Flow matching

Let **x**_1_ be a random variable drawn from an unknown data distribution *q*(**x**_1_). We assume that while samples from *q*(**x**_1_) are available, the density function itself is inaccessible. Let *p*_0_ denote a simple base distribution, such as the standard normal ${p}_{0}({\bf{x}})={\mathcal{N}}({\bf{x}}| 0,{\bf{I}})$, and let *p*_1_ be a distribution that closely approximates *q*. The flow matching objective is then formulated to learn a continuous transformation or probability path, that transports samples smoothly from *p*_0_ to *p*_1_.

A simple way to construct a target probability path is via a mixture of simpler conditional paths^39^. Given a data sample **x**_1_, we denote by *p*_*t*_(**x**∣**x**_1_) a conditional probability path such that it satisfies *p*_0_(**x**∣**x**_1_) = *p*_0_ at *t* = 0, and we design *p*_1_(**x**∣**x**_1_) to be concentrated around **x**_1_ (e.g., ${p}_{1}({\bf{x}}| {{\bf{x}}}_{1})={\mathcal{N}}({\bf{x}}| {{\bf{x}}}_{1},{\sigma }^{2}{\bf{I}})$ for small *σ* > 0). Marginalizing over the unknown data distribution *q*(**x**_1_) gives the marginal probability path^39^

8 $${p}_{t}({\bf{x}})=\int\,{p}_{t}({\bf{x}}| {{\bf{x}}}_{1})q({{\bf{x}}}_{1}){\rm{d}}{{\bf{x}}}_{1},$$

which at *t* = 1 yields

9 $${p}_{1}({\bf{x}})=\int\,{p}_{1}({\bf{x}}| {{\bf{x}}}_{1})q({{\bf{x}}}_{1}){\rm{d}}{{\bf{x}}}_{1}\approx q({\bf{x}}).$$

One can also define a marginal vector field by “mixing” the conditional fields^39^:

10 $${{\bf{v}}}_{t}({\bf{x}})=\int{{\bf{u}}}_{t}({\bf{x}}| {{\bf{x}}}_{1})\frac{{p}_{t}({\bf{x}}| {{\bf{x}}}_{1})q({{\bf{x}}}_{1})}{{p}_{t}({\bf{x}})}{\rm{d}}{{\bf{x}}}_{1},$$

where **u**_*t*_(**x**∣**x**_1_) generates the conditional path *p*_*t*_(**x**∣**x**_1_). This aggregation yields the correct vector field for the marginal path *p*_*t*_(**x**).

#### Gaussian probability path

The flow matching objective works with any choice of probability path and vector field. We can construct *p*_*t*_(**x**∣**x**_1_) and **u**_*t*_(**x**∣**x**_1_) for a general family of Gaussian conditional paths^39^. Namely, we set

11 $${p}_{t}({\bf{x}}| {{\bf{x}}}_{1})={\mathcal{N}}\left({\bf{x}}| {{\boldsymbol{\mu }}}_{t}({{\bf{x}}}_{1}),{\sigma }_{t}{({{\bf{x}}}_{1})}^{2}{\bf{I}}\right),$$

where ${{\boldsymbol{\mu }}}_{t}\in {{\mathbb{R}}}^{d}$ is a time-dependent mean and *σ*_*t*_ ∈ (0, 1] a time-dependent standard deviation. We require **μ**_0_(**x**_1_) = 0, *σ*_0_(**x**_1_) = 1 so that ${p}_{0}({\bf{x}}| {{\bf{x}}}_{1})={\mathcal{N}}({\bf{x}}| 0,{\bf{I}})$ and **μ**_1_(**x**_1_) = **x**_1_, ${\sigma }_{1}({{\bf{x}}}_{1})={\sigma }_{\min }$ with ${\sigma }_{\min }\ll 1$. Thus, *p*_1_(**x**∣**x**_1_) concentrates at **x**_1_.

Among the infinitely many vector fields generating this path, Lipman et al. proposed to choose the simplest affine flow conditioned on **x**_1_^39^:

12 $${\psi }_{t}({\bf{x}})={\sigma }_{t}({{\bf{x}}}_{1}){\bf{x}}+{{\boldsymbol{\mu }}}_{t}({{\bf{x}}}_{1}),$$

which pushes the standard normal ${p}_{0}({\bf{x}}| {{\bf{x}}}_{1})={\mathcal{N}}({\bf{x}}| 0,{\bf{I}})$ to *p*_*t*_(**x**∣**x**_1_) via

13 $${[{\psi }_{t}]}_{*}{p}_{0}({\bf{x}})={p}_{t}({\bf{x}}| {{\bf{x}}}_{1}).$$

The associated conditional vector field then follows from differentiating the flow:

14 $$\frac{d}{dt}{\psi }_{t}({\bf{x}})={{\bf{u}}}_{t}\left({\psi }_{t}({\bf{x}})| {{\bf{x}}}_{1}\right).$$

Reparameterizing *p*_*t*_(**x**∣**x**_1_) in terms of just **x**_0_ gives the conditional flow matching (CFM) loss:

15 $${{\mathcal{L}}}_{{\rm{CFM}}}(\theta )={{\mathbb{E}}}_{t \sim {\mathcal{U}}[0,1],q({{\bf{x}}}_{1}),{p}_{0}({{\bf{x}}}_{0})}{\big|\big| {{\bf{v}}}_{t}^{\theta }\left({\psi }_{t}({{\bf{x}}}_{0})\right)-{{\bf{u}}}_{t}({\psi }_{t}({{\bf{x}}}_{0})| {{\bf{x}}}_{1})\big|\big| }^{2},$$

where *θ* are learnable parameters of a neural network model. ${\mathcal{U}}[0,1]$ is a uniform distribution over the interval [0, 1]. The CFM loss have identical gradients w.r.t. *θ* as the FM loss Eq. (1), but is simpler to compute. Thus, it is the common choice for flow matching training^39^.

In practice, we use a regression based objective^40^

16 $${{\mathcal{L}}}_{{\rm{CFM}}}^{{\prime} }[\theta ]={{\mathbb{E}}}_{t \sim {\mathcal{U}}[0,1],q({{\bf{x}}}_{1}),{p}_{0}({{\bf{x}}}_{0})}\left[\frac{1}{2}| {{\bf{v}}}_{t}^{\theta }({\psi }_{t}({{\bf{x}}}_{0})){| }^{2}-{{\bf{u}}}_{t}({\psi }_{t}({{\bf{x}}}_{0})| {{\bf{x}}}_{1})\cdot {{\bf{v}}}_{t}^{\theta }({\psi }_{t}({{\bf{x}}}_{0}))\right],$$

which yields more stable training.

Given the Gaussian probability path *p*_*t*_(**x**∣**x**_1_) in equation (11), and the corresponding flow map *ψ*_*t*_ in equation (12), the conditional vector field Eq. (14) has the form

17 $${{\bf{u}}}_{t}({\bf{x}}| {{\bf{x}}}_{1})=\frac{\sigma ^{\prime} ({{\bf{x}}}_{1})}{{\sigma }_{t}({{\bf{x}}}_{1})}\left({\bf{x}}-{{\boldsymbol{\mu }}}_{t}({{\bf{x}}}_{1})\right)+{\boldsymbol{\mu }}_{t}^{\prime} ({{\bf{x}}}_{1}).$$

#### Optimal transport path

A natural choice for probability paths is to define the mean and the std to simply change linearly in time:

18 $${{\boldsymbol{\mu }}}_{t}({{\bf{x}}}_{1})=t{{\bf{x}}}_{1},\qquad {\sigma }_{t}({{\bf{x}}}_{1})=1-\left(1-{\sigma }_{\min }\right)t.$$

Then this path is generated by the velocity field^39^

19 $${{\bf{u}}}_{t}({\bf{x}}| {{\bf{x}}}_{1})=\frac{{{\bf{x}}}_{1}-\left(1-{\sigma }_{\min }\right){\bf{x}}}{1-\left(1-{\sigma }_{\min }\right)t}.$$

The conditional flow that corresponds to **u**_*t*_(**x**∣**x**_1_) is

20 $${\psi }_{t}({\bf{x}})=t{{\bf{x}}}_{1}+(1-(1-{\sigma }_{\min })t){\bf{x}}.$$

Allowing the mean and std to change linearly not only leads to simple and intuitive paths, but it is actually also optimal in the following sense. The conditional flow *p*_*t*_(**x**∣**x**_1_) is in fact the optimal transport displacement map between the two Gaussians ${p}_{0}({\bf{x}}| {{\bf{x}}}_{1})={\mathcal{N}}({\bf{x}}| 0,{\bf{I}})$ and *p*_1_(**x**∣**x**_1_).

Using the conditional probability Eq. (11), we can evaluate the probability of a model predicted path by

21 $${p}_{t}^{\theta }({\bf{x}}| {{\bf{x}}}_{1})=\frac{1}{\sqrt{2\pi {\sigma }_{t}^{2}}}\exp \left[-\frac{{({\bf{x}}-{{\boldsymbol{\mu }}}_{t}^{\theta })}^{2}}{2{\sigma }_{t}^{2}}\right],$$

where the expectation of ${{\boldsymbol{\mu }}}_{t}^{\theta }$ can be evaluated by substituting the model predicted velocity field ${{\bf{v}}}_{t}^{\theta }({\psi }_{t}({{\bf{x}}}_{0}))$ in Eq. (19):

22 $${{\boldsymbol{\mu }}}_{t}^{\theta }=\frac{{{\bf{x}}}_{1}-(1-(1-{\sigma }_{\min })t){{\bf{v}}}_{t}^{\theta }({\psi }_{t}({{\bf{x}}}_{0}))}{1-{\sigma }_{\min }}=t{{\bf{v}}}_{t}^{\theta }({\psi }_{t}({{\bf{x}}}_{0})).$$

Substituting Eq. (18) and Eq. (22) in Eq. (21) yields the probability of a model predicted path:

23 $${p}_{t}^{\theta }({\bf{x}}| {{\bf{x}}}_{1})=\frac{1}{\sqrt{2\pi {(1-(1-{\sigma }_{\min })t)}^{2}}}\exp \left[-\frac{{\left({\bf{x}}-t{{\bf{v}}}_{t}^{\theta }({\psi }_{t}({{\bf{x}}}_{0}))\right)}^{2}}{2{(1-(1-{\sigma }_{\min })t)}^{2}}\right].$$

At *t* → 1, this distribution converges to a delta-like sharp Gaussian with zero variance.

#### Conditional modified Jensen-Shannon divergence loss

Besides the CFM loss, we also use a symmetrized and smoothed version of the KL divergence loss to measure the discrepancy between the predicted probability path and the optimal transport probability path. Similar to the FM loss, the KL divergence loss Eq. (2) is intractable. Instead, we propose a conditional KL divergence loss:

$$\begin{array}{l}{{\mathcal{L}}}_{{\rm{CKL}}}(\theta,{\bf{x}})={{\mathbb{E}}}_{t,q({{\bf{x}}}_{1}),{p}_{0}({{\bf{x}}}_{0})}{D}_{{\rm{KL}}}({p}_{t}({\bf{x}}| {{\bf{x}}}_{1})\parallel {p}_{t}^{\theta }({\bf{x}}| {{\bf{x}}}_{1}))\\={{\mathbb{E}}}_{t,q({{\bf{x}}}_{1}),{p}_{0}({{\bf{x}}}_{0})}\frac{1}{\sqrt{2\pi }(1-(1-{\sigma }_{\min })t)}\exp \left[-\frac{{\left({\bf{x}}-{{\boldsymbol{\mu }}}_{t}({{\bf{x}}}_{1})\right)}^{2}}{2{(1-(1-{\sigma }_{\min })t)}^{2}}\right]\\ \left(-\frac{1}{2{(1-(1-{\sigma }_{\min })t)}^{2}}\left({({\bf{x}}-{{\boldsymbol{\mu }}}_{t}({{\bf{x}}}_{1}))}^{2}-{({\bf{x}}-t{{\bf{v}}}_{t}^{\theta }({\psi }_{t}({{\bf{x}}}_{0})))}^{2}\right)\right).\end{array}$$

In practice, we set the random position **x** to **0**. To mitigate the numerical instability caused by the denominator term $(1-(1-{\sigma }_{\min })t)$, we make two modifications to Eq. (24). Firstly, we drop the normalizing factor $\frac{1}{\sqrt{2\pi }(1-(1-{\sigma }_{\min })t)}$, which only affects the relative weighting of the loss across different *t*. Secondly, we compute the KL divergence between two alternative Gaussian probabilities:

24 $${{\mathcal{L}}}_{{\rm{CKL}}}^{{\prime} }(\theta )={{\mathbb{E}}}_{t,q({{\bf{x}}}_{1}),{p}_{0}({{\bf{x}}}_{0})}\exp \left[-\frac{{({{\boldsymbol{\mu }}}_{t}({{\bf{x}}}_{1}))}^{2}}{2}\right]\left(-\frac{1}{2}\left({({{\boldsymbol{\mu }}}_{t}({{\bf{x}}}_{1}))}^{2}-{(t{{\bf{v}}}_{t}^{\theta }({\psi }_{t}({{\bf{x}}}_{0})))}^{2}\right)\right),$$

where both distributions share the same mean as the actual probability path but have a standard deviation of 1.

While KL divergence is a measure of how different two distributions are, it is not a metric. In particular, it is not symmetric in the two distributions and does not satisfy the triangle inequality. Therefore, we use a symmetric variant, the Jensen-Shannon (JS) divergence. Let *P* be the reference distribution, and *Q* be the predicted distribution, JS divergence is defined by

25 $${D}_{{\rm{JS}}}=\frac{1}{2}{D}_{{\rm{KL}}}(P\parallel M)+\frac{1}{2}{D}_{{\rm{KL}}}(Q\parallel M),\,M=\frac{1}{2}(P+Q).$$

The square root of JS divergence is a metric.

#### Dynamic optimal transport

Dynamic optimal transport, as introduced by Benamou and Brenier^41^, presents the squared Wasserstein-2 distance ${W}_{2}^{2}({p}_{0},{p}_{1})$ in a dynamic formulation. This formulation involves optimizing the squared *L*^2^ norm of a time-varying vector field **u**_*t*_(**x**), which transports samples from **x**_0_ ~ *p*_0_(**x**_0_) to **x**_1_ ~ *p*_1_(**x**_1_) according to an ordinary differential equation $\frac{d{\psi }_{t}({\bf{x}})}{dt}={{\bf{u}}}_{t}({\bf{x}})$. Mathematically, this dynamic formulation is expressed as

26 $${W}_{2}^{2}({p}_{0},{p}_{1}):={\mathop{\inf }\limits_{\pi \in \Pi ({p}_{0},{p}_{1})}}\int_{0}^{1}\int_{{{\mathbb{R}}}^{d}}\frac{1}{2}\parallel {{\bf{u}}}_{t}(x){\parallel }^{2}{p}_{t}({\bf{x}})d{\bf{x}}dt,$$

where *Π*(*p*_0_, *p*_1_) is the set of couplings with *p*_0_ and *p*_1_. This approach is in contrast with the flow matching method by Lipman et al.^39^, where the probability path is given by Gaussian distributions. In ReactOT^26^, Duan et al. assumed the optimal coupling to be ${\pi }^{*} \sim (\frac{R+P}{2},TS)$. This enables a flow matching model that generates a unique TS for a given reactant-product pair.

### MPNN

An atomic graph is embedded in 3-dimensional (3D) Euclidean space, where each node represents an atom and edges connect nodes if the corresponding atoms are within a given cutoff distance of each other. The cutoff was set to 12 Å. We represent the state of each node *i* by a tuple *σ*_*i*_ = (**r**_*i*_, *z*_*i*_, **h**_*i*_), where ${\bf{r}}\in {{\mathbb{R}}}^{3}$ is the position of atom *i*, *z*_*i*_ the chemical element, and **h**_*i*_ are learnable features. MPNN parameterizes a mapping from a graph to a target space, such as the velocity field, through multiple message passing layers.

#### Node feature

Firstly, the one-hot coded chemical element *z*_*i*_ is passed through a SiLU-activated multi-layer perception (MLP) to obtain the node feature ${{\mathcal{V}}}_{i}$.

#### Construction of edge features

An edge feature ${\mathcal{E}}={\oplus }_{l=0}^{{l}_{\max }}{d}_{l}{l}^{p(l)}$ is constructed by concatenating *d*_*l*_ copies of degree-*l* spherical components with parity *p*(*l*) equals even for even *l* and odd for odd *l*, so that the edge features transform as proper SO(3) tensors. This mirrors the standard design in higher-order equivariant MPNNs where messages are built by contracting node features with spherical harmonics of edge directions and radial envelopes, then mapped via Clebsch-Gordan (tensor-product) rules to the desired output irreps^42^.

##### Radial basis

For each edge length *r*_*ji*_ = ∥**r**_*ji*_∥, we compute a set of radial channels

27 $${\phi }_{k}=\frac{1}{2}\left(\cos (\pi r/{r}_{{\rm{c}}})+1\right)1[r < {r}_{{\rm{c}}}]\cdot \exp \left(-{\beta }_{k}{({e}^{-r}-{\nu }_{k})}^{2}\right),\,k=1,\ldots,K,$$

with evenly spaced means *ν*_*k*_ between ${e}^{-{r}_{{\rm{c}}}}$ and $$1[r < {r}_{{\rm{c}}}]=\left\{\begin{array}{ll}1,\quad &{\rm{if}}r < {r}_{{\rm{c}}}\\ 0,\quad &{\rm{if}}r > {r}_{{\rm{c}}}\end{array}\right.$$, and a shared width ${\beta }_{k}=\frac{K}{{\left(\frac{2}{K}(1-{e}^{-{r}_{{\rm{c}}}})\right)}^{2}}$. This yields smooth bounded radial features.

##### Angular basis

For the unit direction ${\hat{{\bf{r}}}}_{ji}={{\bf{r}}}_{ji}/{r}_{ji}$, the block computes real spherical harmonics ${Y}_{l}(\hat{{\bf{r}}})\in {{\mathbb{R}}}^{2l+1}$ up to ${l}_{\max }$. Spherical harmonics are the canonical basis that ensures proper SO(3) transformation.

##### Per-*l* mixing and the edge feature tensor

For each *l*, the radial channels $\phi ({r}_{ji})\in {{\mathbb{R}}}^{K}$ is mapped via a small SiLU-activated MLP to *d*_*l*_ scalar weights ${{\bf{w}}}_{l}({r}_{ji})\in {{\mathbb{R}}}^{{d}_{l}}$. These weights scale each ${Y}_{l}({\hat{{\bf{r}}}}_{ji})\in {{\mathbb{R}}}^{2l+1}$, producing

28 $${{\bf{e}}}_{l}({{\bf{r}}}_{ji})={{\bf{w}}}_{l}\otimes {Y}_{l}({\hat{{\bf{r}}}}_{ji})\in {{\mathbb{R}}}^{{d}_{l}(2l+1)},$$

where  ⊗ denotes tensor product operation. Concatenating over $l=0,\ldots,{l}_{\max }$ yields a flat edge feature tensor **e**_*ji*_ with irreps ${{\mathcal{E}}}_{ji}={\oplus }_{l}{d}_{l}{l}^{p(l)}$.

#### Construction of messages by Clebsch-Gordan contraction

Messages are computed by a learned fully connected tensor product (FCTP) that contracts node features and edge features: ${\bf{{\mathcal{M}}}}(j,i)={\rm{FCTP}}({{\mathcal{V}}}_{i},{{\bf{e}}}_{ji})$, where FCTP enumerates all Clebsch-Gordan (CG) paths ${{\mathcal{V}}}_{i}\otimes {{\mathcal{E}}}_{ji}$ and learns weights for them while preserving equivariance by construction. The invariant components of the resulting messages, denoted **m**(*j*, *i*), are subsequently extracted and propagated during message passing.

##### Reaction messages

The messages of the reactant, TS, and product configurations are concatenated to form a reaction message:

29 $${\bf{m}}(j,i)={\oplus }_{R,TS,P}({{\bf{m}}}_{{\rm{R}}}(\,j,i),{{\bf{m}}}_{{\rm{TS}}}(\,j,i),{{\bf{m}}}_{{\rm{P}}}(\,j,i)),$$

where  ⊕ _*R*,*TS*,*P*_ denotes concatenation of the reactant, TS, and product messages. Note that in practice, the **m**_TS_(*j*, *i*) is built using a flow matching sample at time *t*, while **m**_R_(*j*, *i*) and **m**_P_(*j*, *i*) are built using the true configurations.

#### Object-aware message passing

Message passing is implemented with object-aware equivariance, where the model prediction is equivariant w.r.t. the relative rotation and translation of various molecules in a system^17^.

For atoms belonging to the same molecule, the concatenated vector of the message and the associated node features, $\oplus ({{\mathcal{V}}}_{i},{{\mathcal{V}}}_{j},{\bf{m}}(j,i))$, is passed through an SiLU-activated MLP to produce *M*_*ji*_. These outputs are then aggregated to form two components: a vector feature, ${{\bf{H}}}_{i}={\sum }_{j\in {\mathcal{N}}(i)}{M}_{ji}{{\bf{r}}}_{ji}$, and a scalar feature, ${\sum }_{j\in {\mathcal{N}}(i)}{M}_{ji}{{\mathcal{V}}}_{j}$. The scalar feature is concatenated with ${{\mathcal{V}}}_{i}$ and passed through another SiLU-activated MLP to yield the updated scalar feature ${H}_{i}^{intra}$.

For atoms belonging to different molecules, only the node features are aggregated, i.e., $\oplus ({{\mathcal{V}}}_{i},{{\mathcal{V}}}_{j})$. This concatenated representation is passed through a SiLU-activated MLP to produce ${M}_{ji}^{{\rm{inter}}}$. The resulting messages are aggregated as ${\sum }_{j\in {\mathcal{N}}(i)}{M}_{ji}^{{\rm{inter}}}{{\mathcal{V}}}_{j}$, which is then concatenated with ${{\mathcal{V}}}_{i}$ and processed by another SiLU-activated MLP to obtain the inter-molecular scalar feature ${H}_{i}^{{\rm{inter}}}$.

The intra- and inter-molecular scalar features are concatenated $\oplus ({H}_{i}^{{\rm{intra}}},{H}_{i}^{{\rm{inter}}})$ and passed through a SiLU-activated MLP to produce the final updated scalar feature *H*_*i*_.

#### Readout by an equivariant transformer

To efficiently capture the intricate interactions between atoms both within and across molecules, we employ the equivariant transformer (ET)^43^ to update the scalar and vector features obtained in the previous section. The ET architecture preserves object-aware SE(3) symmetry, ensuring consistent treatment of rotations and translations, while effectively modeling complex geometric relationships. It exhibits strong scalability across large and diverse datasets, showing substantial performance gains when trained on extended molecular databases, as demonstrated by our results in the main text. This advantage is further reinforced by its computational efficiency, where the ET trains approximately 3-4 × faster per epoch than existing architectures such as React-OT.

The ET outputs a vector feature ${{\bf{H}}}_{i}^{{\prime} }$ and a scalar feature ${H}_{i}^{{\prime} }$. Finally, the vector feature ${{\bf{H}}}_{i}^{{\prime} }$ is passed through a simple linear layer to produce the predicted velocity field **v**_*i*_.

### Model training

We train the model using the AdamW optimizer^44^. The learning rate is initialized at 1 × 10^−4^ and linearly warmed up to 5 × 10^−4^ over the first 10 epochs according to

30 $$lr=1\times 1{0}^{-4}+(5\times 1{0}^{-4}-1\times 1{0}^{-4})\times \frac{{\rm{epoch}}+1}{10},$$

and then decays to 3 × 10^−5^ following a cosine schedule:

31 $$lr=3\times 1{0}^{-5}+(5\times 1{0}^{-4}-3\times 1{0}^{-5})\times 0.5\times \left(1+\cos \left(\pi \frac{{\rm{epoch}}-10}{600}\right)\right).$$

During finetuning, the learning rate starts at 1 × 10^−4^ and decays to 3 × 10^−5^ with the same cosine decay strategy. The batch size is set to 64.

### Evaluation

#### DFT

Energy evaluation was performed using DFT (*ω*B97x/6-31G(d)) for consistent comparison with the Transition1x database. For saddle point optimization, *ω*B97x/def2-TZVP was used for more reliable convergence. Energy evaluation was carried out using the pySCF package^45^, and optimization was carried out using the Psi4 package^46^.

#### IRC calculation

IRC performed to verify the optimized TS is carried out using the Psi4 package^46^. The resulting endpoints of the IRC integration are further optimized to energy minimums. The comparison of IRC endpoints and input reactants and products is based on the adjacency matrix computed by the TAFFI package^32^.

#### The characteristic chemical accuracy

Under transition-state theory, the rate constant scales as $k\propto \exp (-\Delta {G}^{\ddagger }/RT)$. An error *δ*Δ*G*‡ in the activation free energy translates into a multiplicative error of $\exp (\delta \Delta G\ddagger /RT)$ to the reaction rate. A ten-fold error in rate corresponds to *δ*Δ*G*‡ = *RT*ln10 ≈ 2.303*RT*. At a representative synthetic temperature of 70 °C, this error is 2.303*RT* ≈ 1.58 kcal/mol.

#### Measurement of top-*k* accuracy

For each reaction entry in the Transition1x test set, we extract the reactant and generate 240 corresponding product candidates. All generated samples are then combined, and unique reactants are identified. For each unique reactant, we compile the list of associated products and rank them based on their occurrence frequency. Molecular comparisons are performed using adjacency matrices computed with the TAFFI package^32^.

### Details of the reaction network exploration workflow

#### Physically motivated criteria for product pruning

The following criteria are either used or suggested to be useful for product pruning in this work

1. The product configuration must be at a local minimum, verified by vibrational analysis;
2. The product configuration can not be the same as the reactant according to adjacency matrix comparisons (This criterion is only relevant for the small-molecule reactions studied in this work and does not apply to larger molecules, for which distinct conformers sharing the same adjacency matrix become important);
3. Radical products are excluded from the list of allowed configurations for further reactions due to the typically high transition barriers. This is confirmed by S3.3, where we list the Gibbs transition barrier of all the generated first-step KHP decomposition reactions. The three radical products all result from higher transition barriers (92.703 kcal/mol, 72.119 kcal/mol, 70.842 kcal/mol) than the majority of the generated reactions.
4. (Suggested) For KHP decomposition, small molecules can be removed from the list of allowed configurations to react again. This is not because MolGEN fails to predict the correct products, but because we are modeling a decomposition-type scenario in which volatile or low-boiling coproducts are effectively removed from the system as they form. In practice, this corresponds to steering the exploration away from recombination pathways (where small fragments recombine into larger molecules) and toward further decomposition, which is the mechanistic regime of interest for KHP. Physically, this is akin to simulating a process in which small molecules are continuously extracted (e.g., by gas flow), thereby driving decomposition forward rather than allowing back-reaction and recombination.

#### Definition of “intended reactions” and “intended TS”

In reaction-network exploration, a TS is classified as an intended TS, and the corresponding reaction as an intended reaction, if, after geometry optimization, it connects the same reactant-product pair as the input reactant-product pair. The intended-TS metric therefore reflects (1) whether the specified reactant-product pair indeed corresponds to an elementary reaction and (2) the reliability of the TS-generation method.

Conversely, unintended TSs remain valuable for reaction-network construction, as they reveal alternative reactions involving different reactant-product pairs.

## Supplementary information

Supplementary Information

Transparent Peer Review file

## Source data

Source Data

## Data Availability

Structures generated in this study are provided in the Source Data file, and deposited in the GitHub repository https://github.com/tuoping/MolGEN and the figshare database under accession code 10.6084/m9.figshare.30576365. Source data are provided in this paper.

## Code availability

The MolGEN codebase is available as an open-source repository for continuous development at https://github.com/tuoping/MolGEN. A release of the code used in this work has been archived on Zenodo^47^.
