# Supplementary material for: Flow matching for reaction pathway generation
Source: Nat Commun. 2026 Jul 17;17:8769. doi: 10.1038/s41467-026-75654-w (PMC13493859; doi:10.1038/s41467-026-75654-w)
Supplement: Supplementary file 1 [file 41467_2026_75654_MOESM1_ESM.pdf]

Supplementary material of “Flow matching for reaction pathway generation”

S1. Additional quantitative results

S1.1. Energy changes following relaxation of the generated transition states

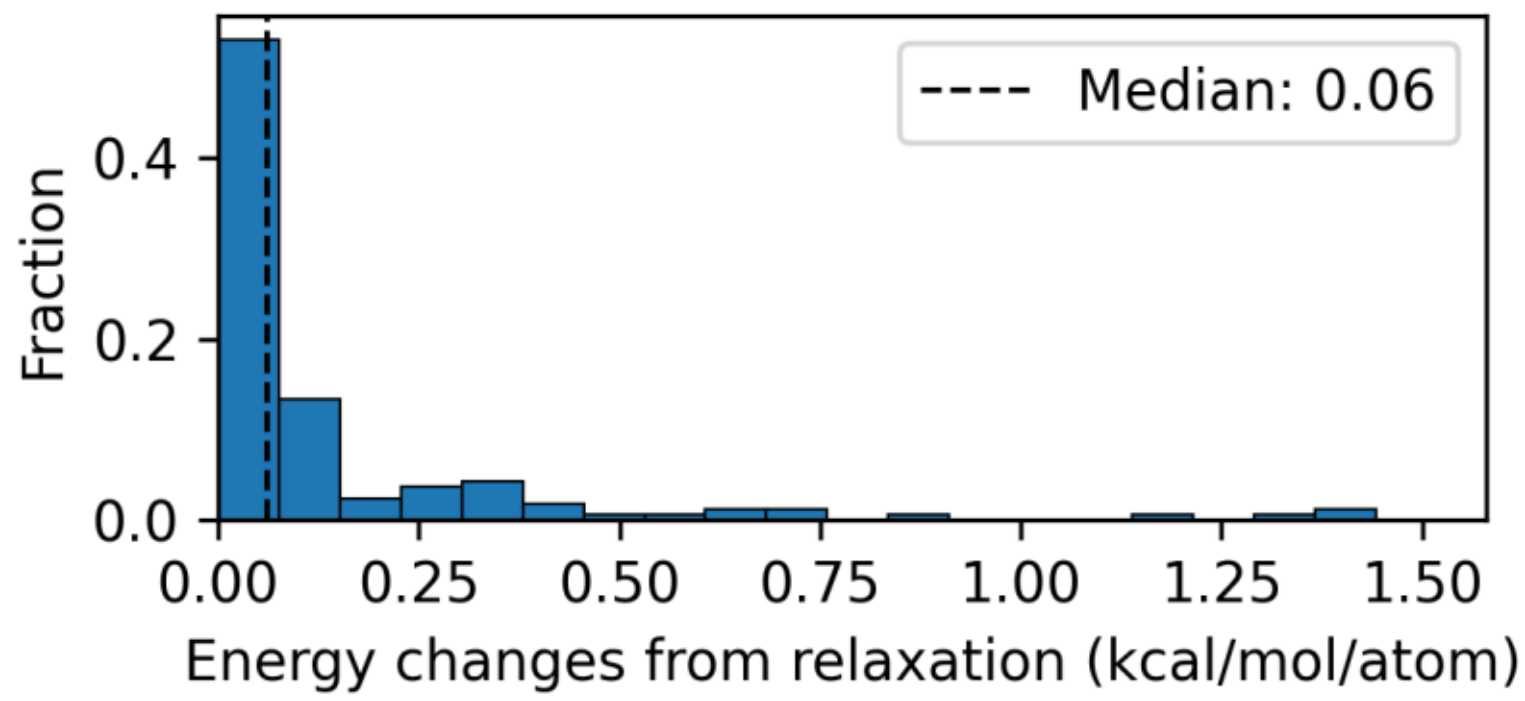

Figure S1 Energy changes following optimization of the transition states generated by MolGEN. Source data are provided as a Source Data file.

Figure S1 shows that optimized transition states (TS) have smaller energy changes than products. This arises from incorporating additional conditioning signals for TS generation, suggesting an effective strategy for reducing prediction errors of generative models.

S2. Ablation tests over different loss functions and databases

Table S1 Ablation tests over different loss functions and databases. The performance metrics include the mean root-mean-squared distance (RMSD), the ratio of predictions within chemical accuracy, defined as a barrier-height error below 1.58 kcal/mol, the ratio of valid transition states (TSs), and the ratio of updated TSs, defined as newly generated TSs with barrier heights more than 0.1 kcal/mol lower than the reference. L1 denotes the flow matching loss, and JS denotes the Jensen-Shannon divergence loss. The training dataset is either the Transition1x dataset [1] or the combined Transition1x and RGD1 dataset [2]. Tests were run on the Transition1x test set.

Training data	Message Passing	Loss	mean RMSD	Chem. Accu. (%)	Ratio of valid TS (%)	Ratio of updated TS
Transition1x	LFETNet	L1	0.172	5.76		
		Pretrained by diffusion; L1	0.143	28.27		
	MolGEN	L1	0.163	8.90		
		Pretrained by diffusion; L1	0.152	9.95		
	MolGEN	L1	0.127	41.36		
		L1; Tuned by JS	0.106	54.38	87.5	7.32
		JS+L1; tuned by JS	0.121	50.26		
		Pretrained by diffusion; L1	0.121	49.74		
RGD1+	LFETNet	L1	0.158	8.90		

Transition1x	MolGEN	L1	0.210	4.19		
	MolGEN	L1	0.115	71.73		
		L1; Tuned by JS	0.119	73.82	76.0	16.44
		JS+L1; tuned by JS	0.100	70.80	89.2	27.87
		JS	0.112	68.98	88.5	30.66

LEFTNet is the model architecture employed in ReactOT [3]. LEFTNet is more expressive than MolGEN but takes roughly 3-4 times longer per training epoch. The results shown here are tested on a model trained from scratch. One interesting observation from the table is that pretraining by diffusion significantly improves LEFTNet's performance.

S3. Reaction pathways of γ-ketohydroperoxide (KHP)

S3.1 KHP geometry

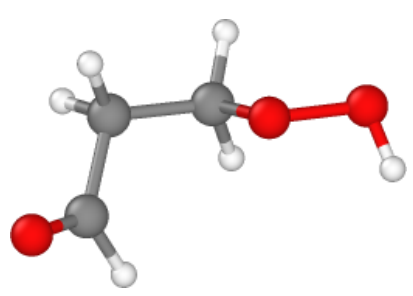

(12 atoms)

O -2.547608387657 -0.611714091433 0.217659185843

C -1.591671539409 -0.270472437685 -0.417876937226

C -0.765125470739 0.957024277346 -0.124643976709

C 0.703694945887 0.754612468289 -0.430173770765

O 1.159376857672 -0.230226458895 0.476038420268

O 2.543750778328 -0.422441078629 0.222883752928

H -1.267113864240 -0.853161729831 -1.308337837025

H -0.926111687863 1.260738017297 0.910212867278

H -1.141934614748 1.758230798867 -0.770192160863

H 1.269579366969 1.679998266663 -0.292047204233

H 0.849905937269 0.409741033887 -1.461630900890

H 2.559948927351 -1.348588916448 -0.043107106191

S3.2 First step reactions

Using KHP as input to a MolGEN model trained for reaction product generation, we generated 1000 products. We calculated each product's adjacency matrix, and categorized the geometries with the same adjacency matrix as the same product. 80 unique products were found. We selected the lowest energy configuration for each unique product and optimized it using DFT (ωB97X/6-31G(d)). Among them, 65

were optimized to a local minimum without imaginary frequencies. 6 were excluded from further steps because their adjacency matrix turned out to be the same as that of the reactant KHP after optimization. The remaining 59 reactions with optimized products were fed into a MolGEN model trained for TS generation.

For each reaction, we generated 30 TS candidates and chose the lowest energy candidates as our final prediction. The 59 selected TS were optimized and subjected to vibration analysis, among which 40 turned out to be valid TS with only one imaginary frequency. Further, IRC calculations were performed on the 40 valid TS and found 14 intended TS.

Here, we first list the product geometry, product energy, and the TS energies of the 14 intended reactions with the intended TSs.

All energies are relative to the energy of the KHP. And we also attach a zip ball of the xyz files of all the configurations. The index of the configurations here corresponds to the naming of the xyz files. The structure files are also available in our GitHub repo: https://github.com/tuoping/MolGEN.

Table S3 List of the 14 intended first-step KHP decomposition products generated by MolGEN. The products with radicals are highlighted in red, and decomposition reactions are highlighted in green; grey, red, and white spheres represent carbon (C), oxygen (O), and hydrogen (H), respectively.

Index	Product geometry	Electronic energies of products (kcal/mol)	Gibbs free energies of products (kcal/mol)	Electronic energies of TSs (kcal/mol)	Gibbs free energies of TSs (kcal/mol)
1	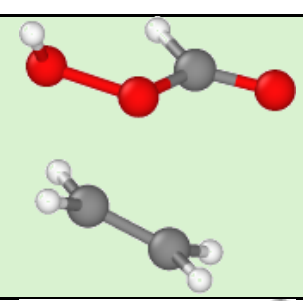	14.388	-29.052	64.969	57.836
2	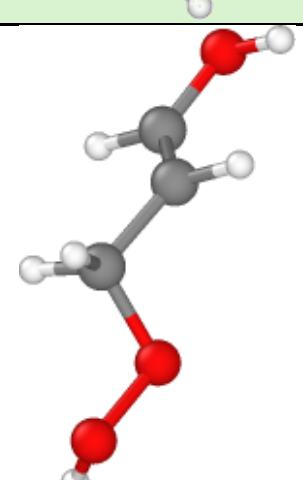	13.188	25.74	72.291	35.974
4	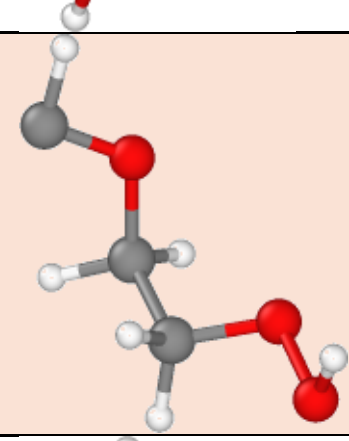	67.44	58.116	122.598	92.703
5	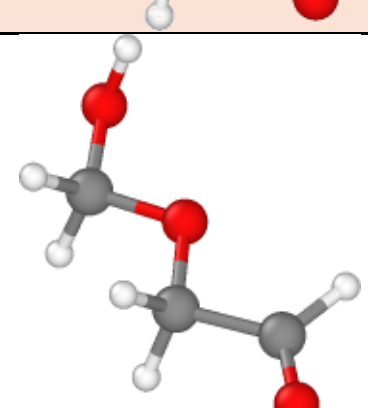	-48.768	-42.612	93.299	48.704
11	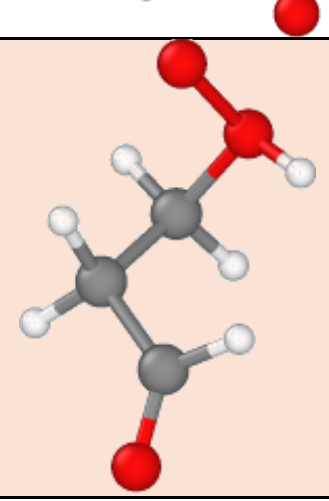	48.78	48.432	85.462	71.119

15	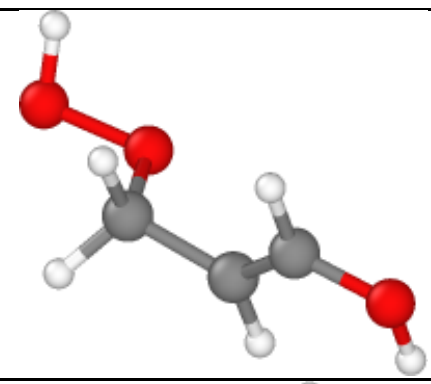	13.176	26.292	74.089	34.314
19	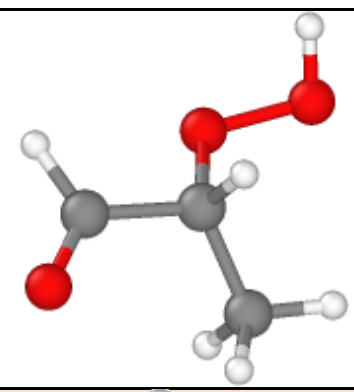	-1.32	-8.856	97.528	68.351
22	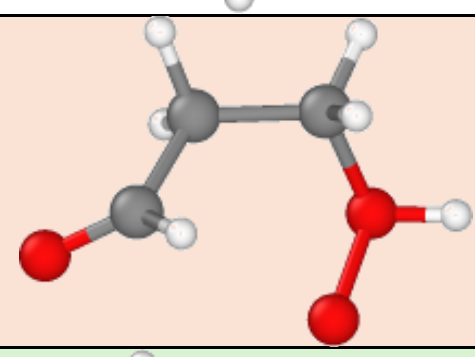	45.408	61.704	82.676	70.842
24	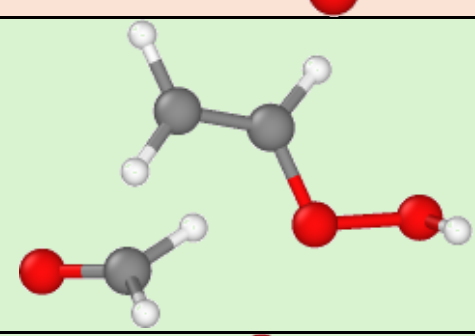	32.808	-55.068	78.599	53.962
25	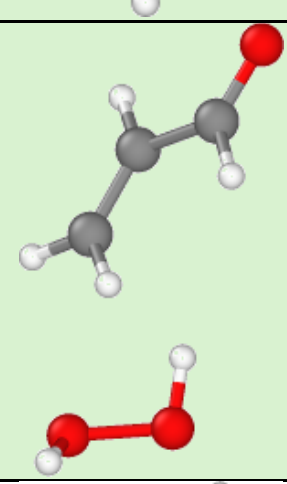	24.156	-25.188	66.058	17.710
30	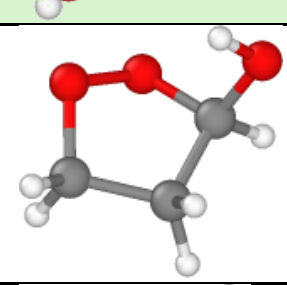	9.264	29.052	32.928	44.276
36	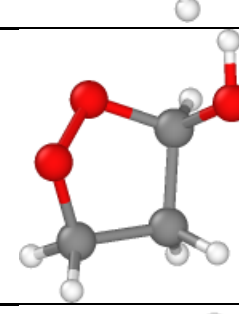	-12.948	39.024	36.375	43.723
37	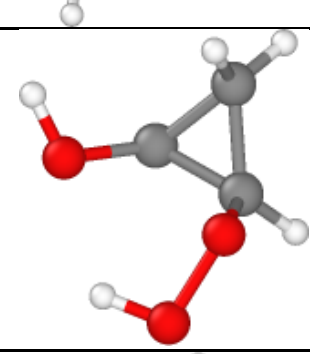	17.88	48.432	97.11	77.207
54	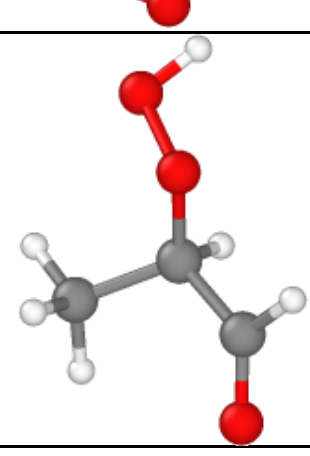	-1.32	-10.236	97.551	68.351

Table S4 List of the geometries and energies of the remaining 59 products (decomposition reactions are highlighted in green).

	Product geometry	Electronic energies of products (kcal/mol)	Gibbs free energies of products (kcal/mol)		Product geometry	Electronic energies of products (kcal/mol)	Gibbs free energies of products (kcal/mol)

0	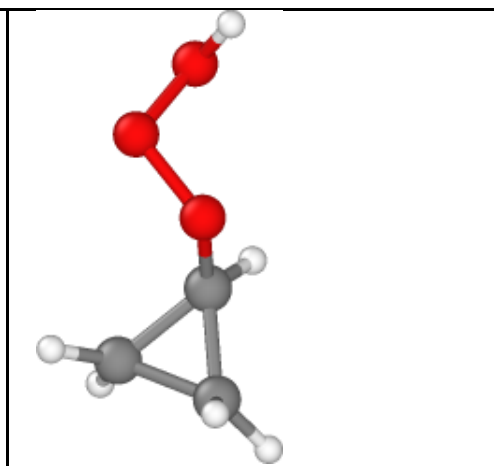	5.068	6.296	3	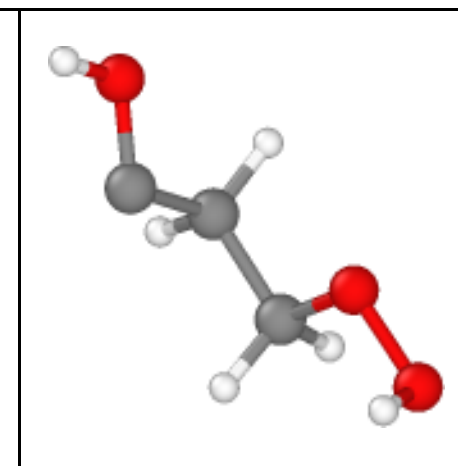	4.571	4.82
6	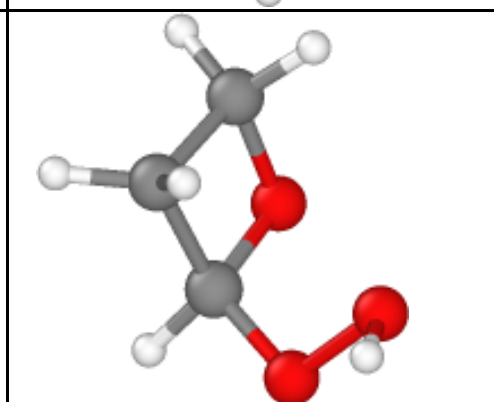	0.718	3.92	7	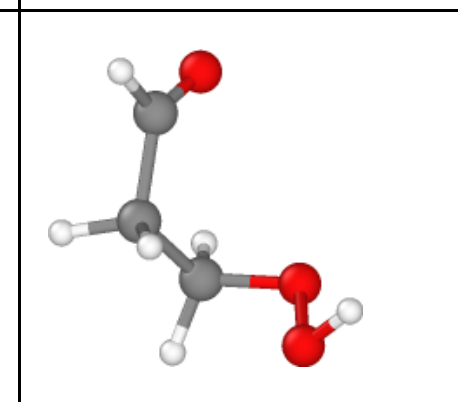	-0.093	-0.069
8	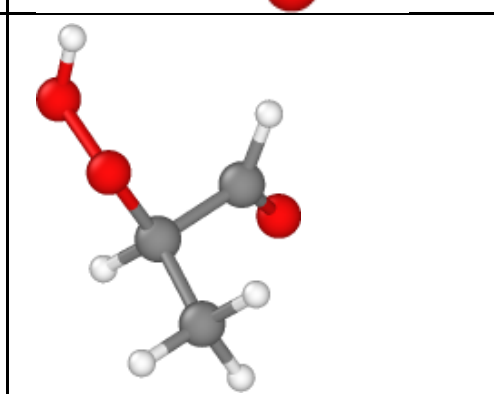	-0.203	-0.138	10	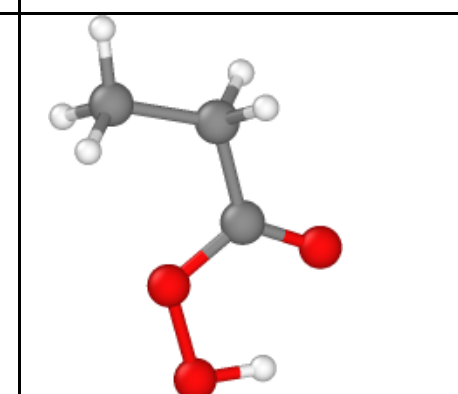	-2.37	0
12	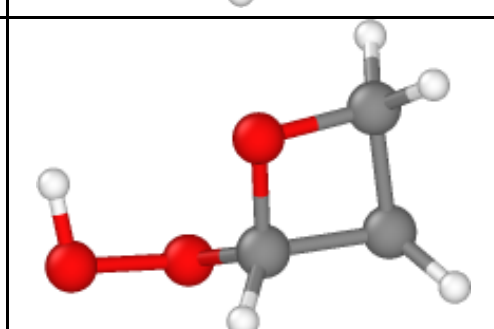	0.573	3.644	13	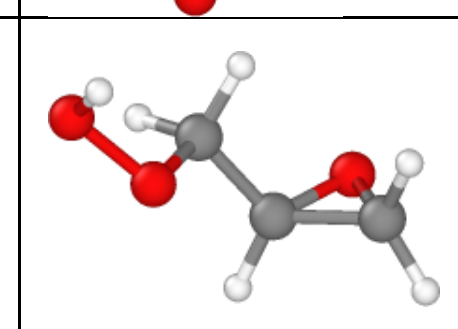	1.749	3.252
14	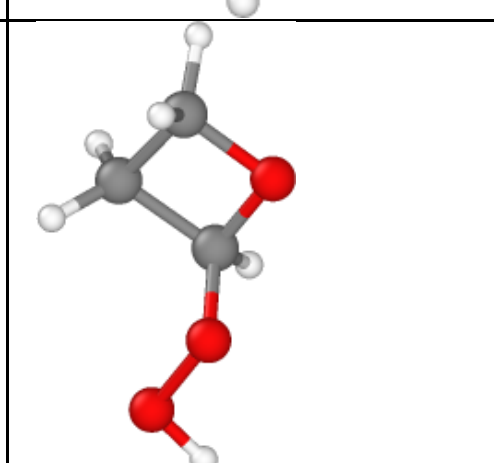	0.935	3.644	16	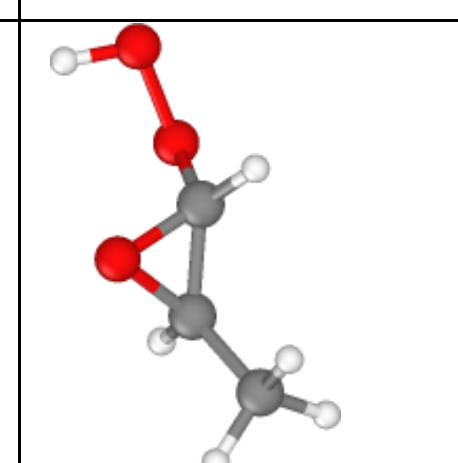	0.771	2.421
17	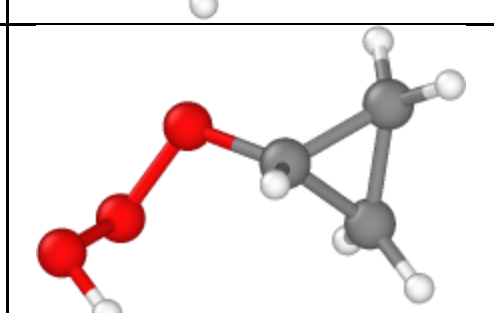	5.25	6.434	18	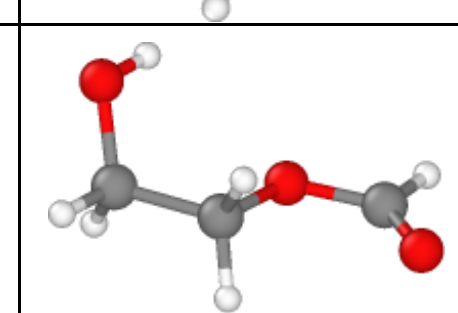	-5.821	-3.505
19	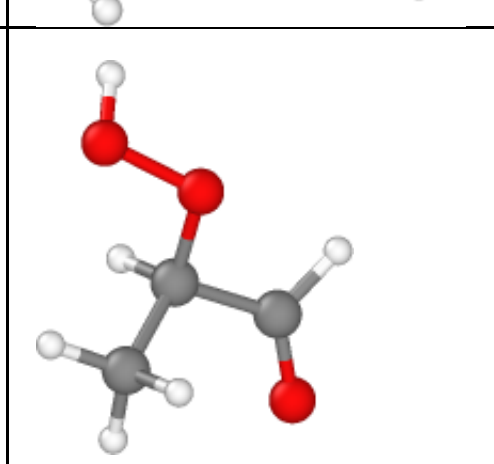	-0.11	-0.738	20	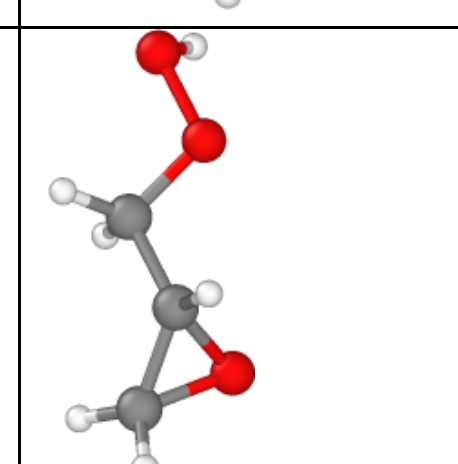	1.712	3.574
21	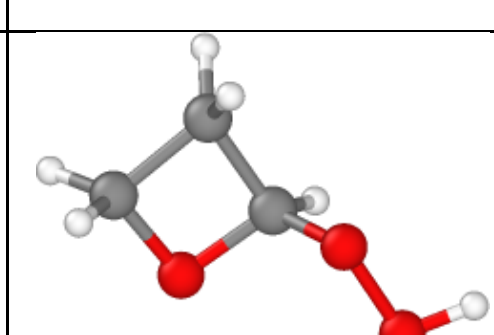	0.789	3.574	23	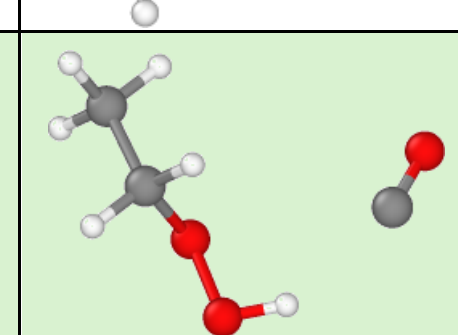	-0.086	-4.704
26	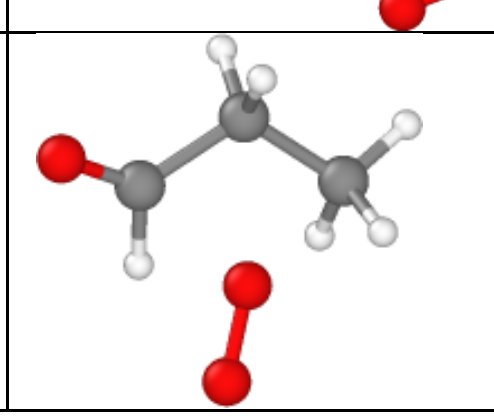	4.231	1.314	27	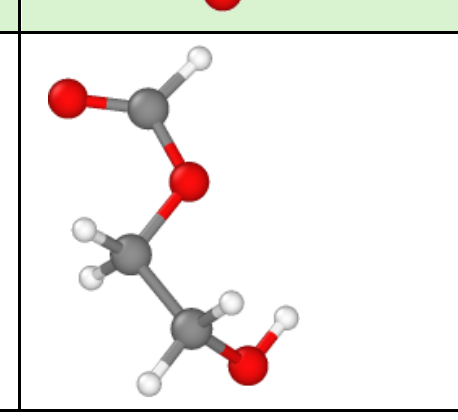	-5.82	-3.505

28	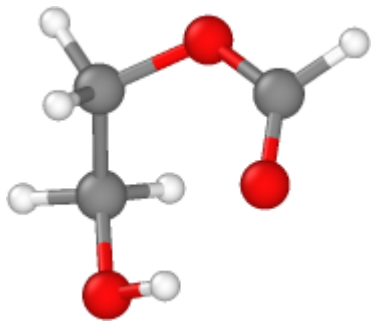	-5.951	-2.675	29	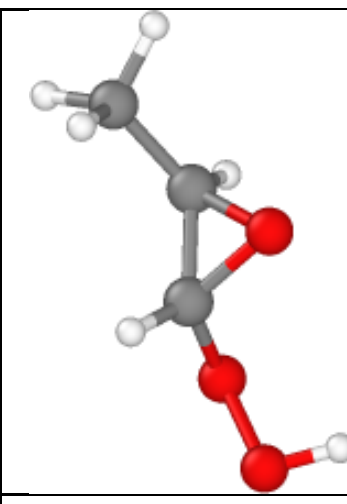	0.772	2.421
31	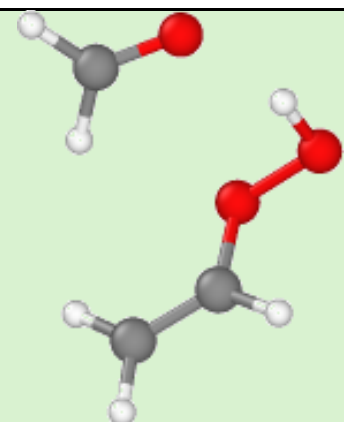	2.147	-0.83	32	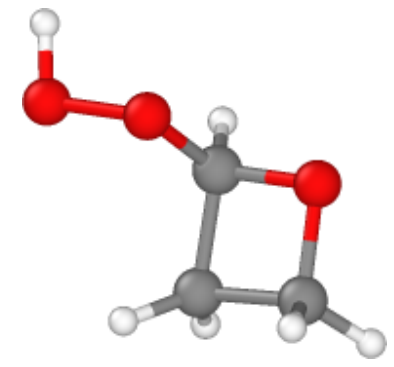	0.935	3.69
33	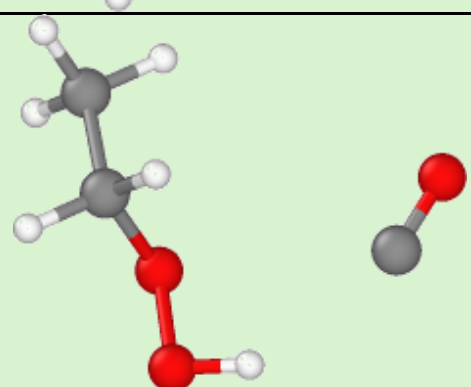	-0.094	-4.174	35	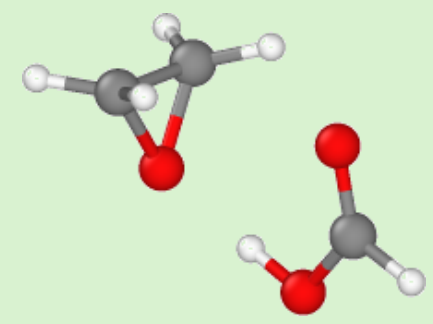	-4.534	-4.013
39	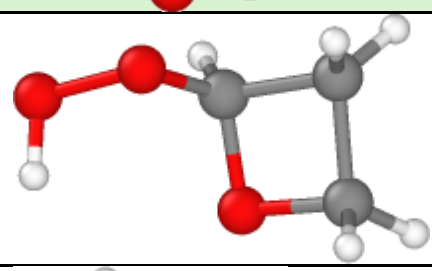	0.573	3.644	40	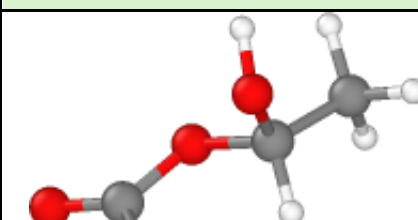	-6.332	-4.335
41	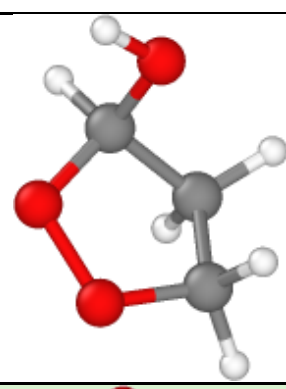	-6.332	2.744	42	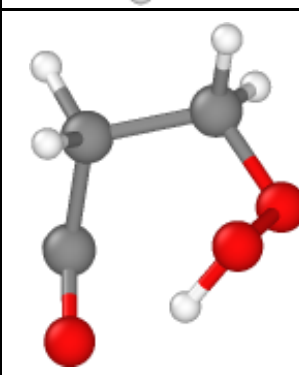	-0.995	1.361
43	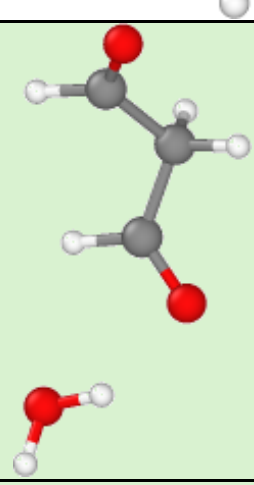	-0.295	1.361	44	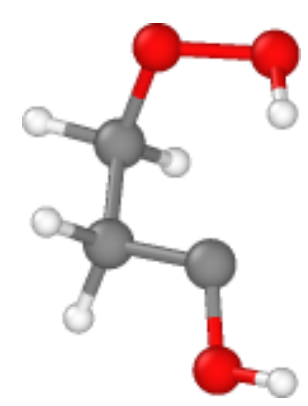	4.122	5.581
45	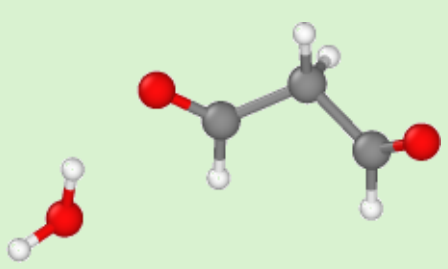	-4.09	-8.002	47	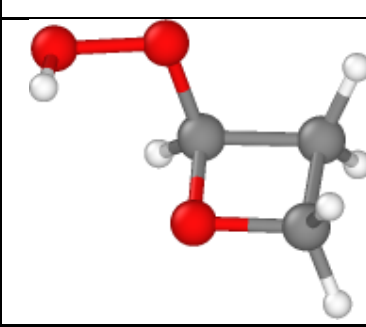	0.573	3.644
48	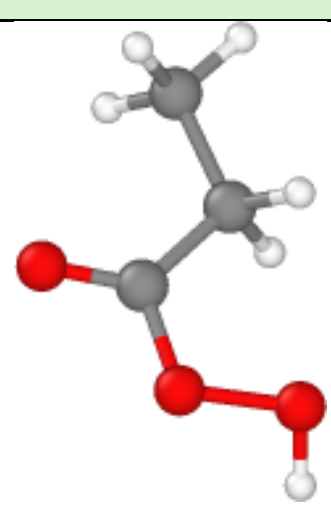	0.573	3.644	49	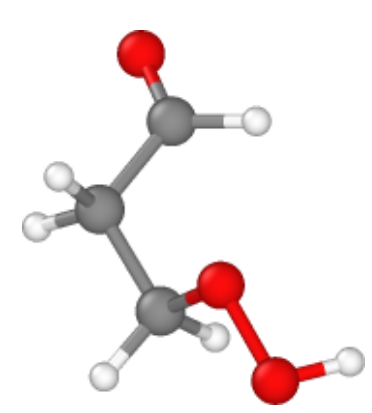	0.017	0.577
50	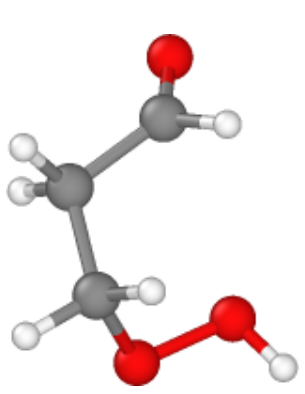	-0.064	0.692	51	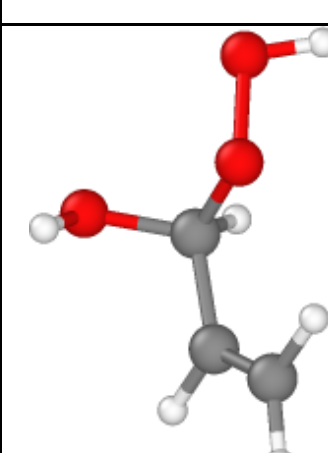	0.954	2.052

52	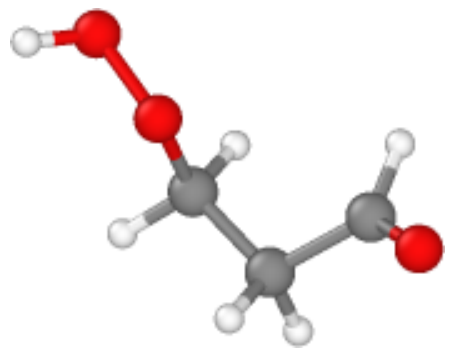	-0.017	-0.046	55	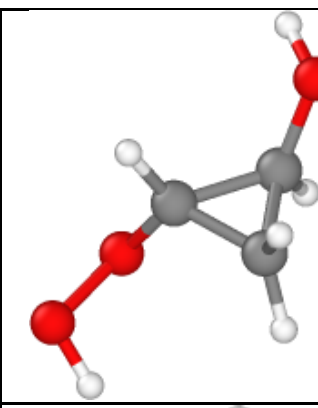	1.642	3.344
56	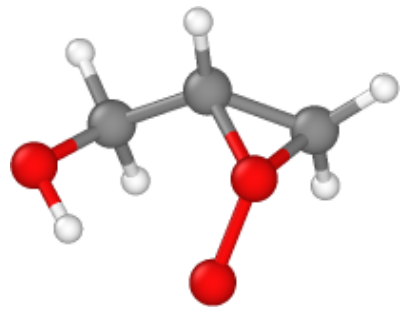	4.804	7.864	57	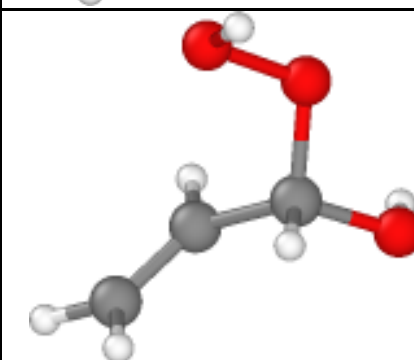	1.166	1.73
59	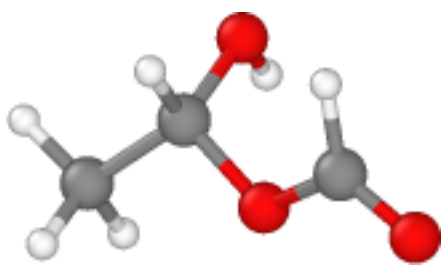	-6.332	-4.289	60	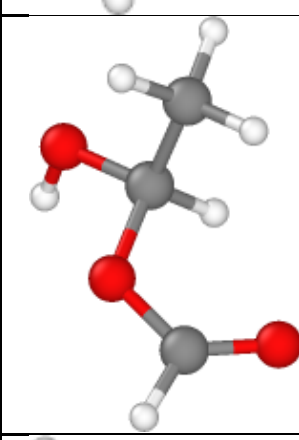	-6.544	-4.105
61	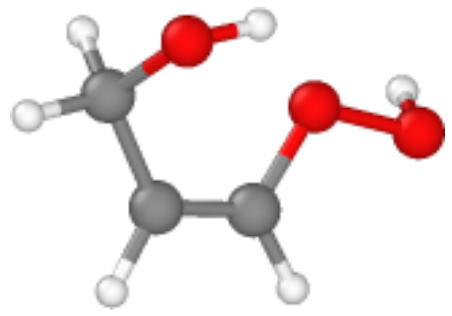	1.522	2.744	62	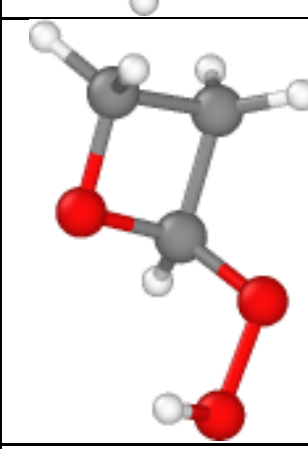	0.572	3.644
63	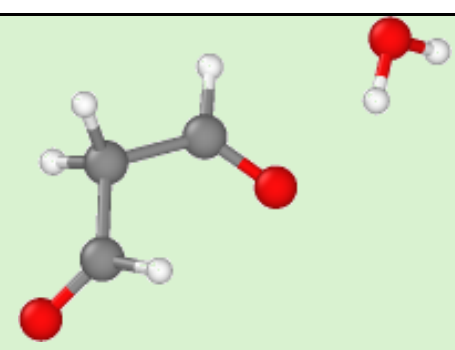	-4.207	-8.694	64	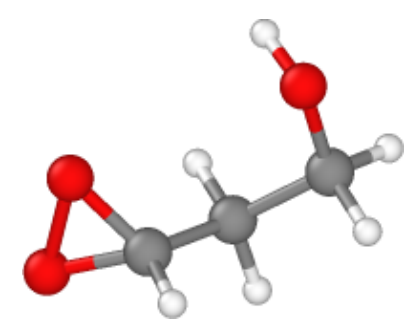	0.609	2.629

S3.3 Second step reactions

11 products from the first step were selected as reactants for further exploration. This time, 500 products were generated for each reactant. Here, we only list the products of the intended second-step reactions. Again, the index here corresponds to the naming of the files.

Reactant: product 1 from the first step:

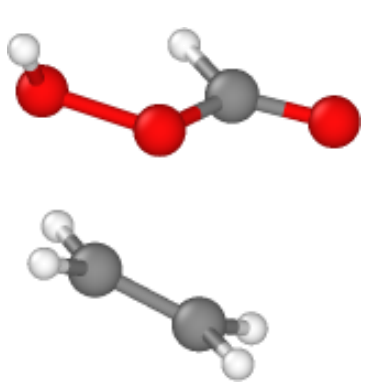

Table S5 List of intended second-step reaction products derived from product 1 of the first step.

	Product geometry		Product geometry		Product geometry		Product geometry
0	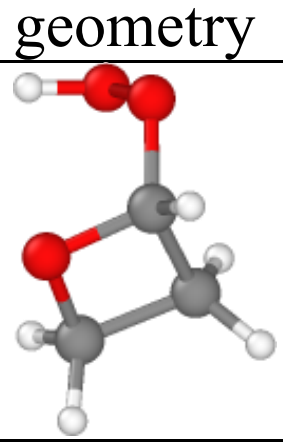	1	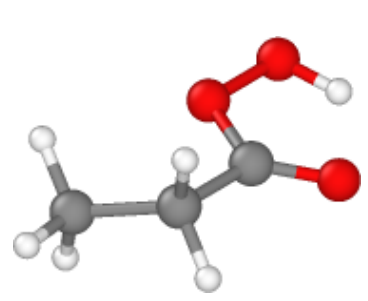	2	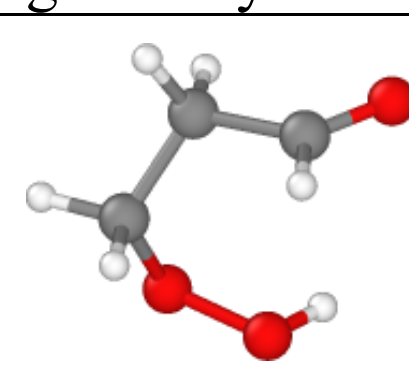	3	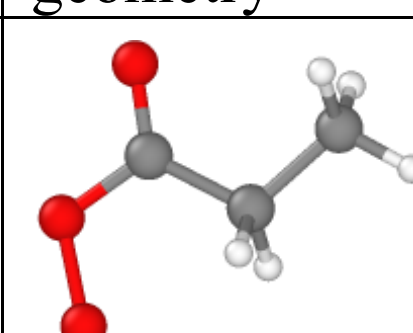

4	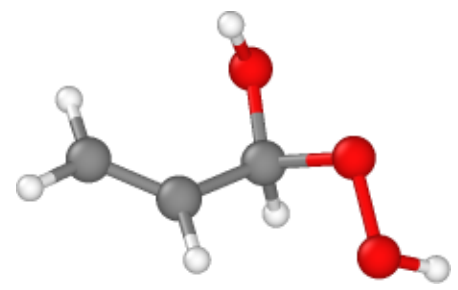	5	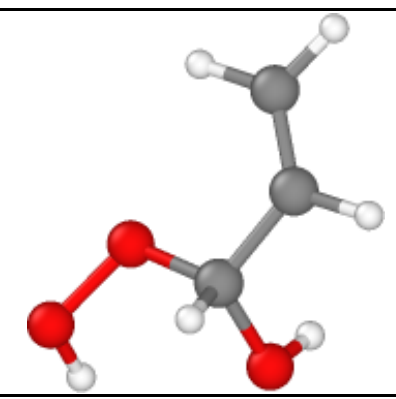	7	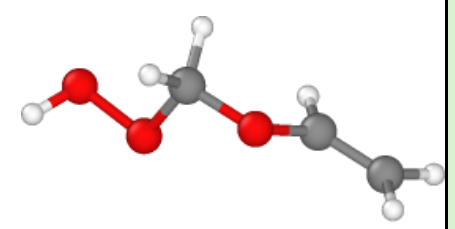	9	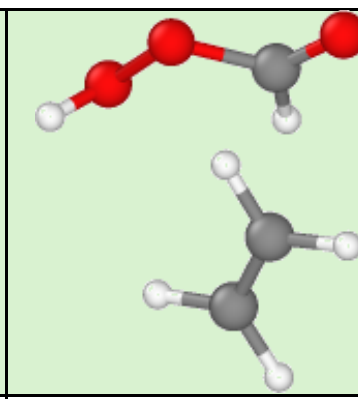
10	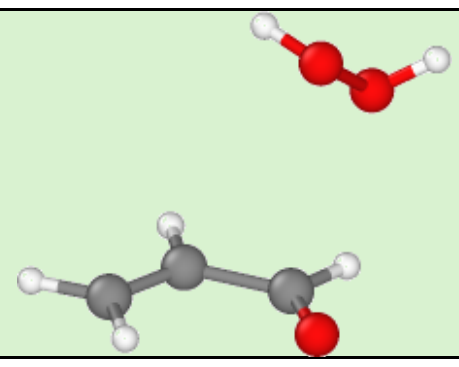						

Reactant: product 5 from the first step:

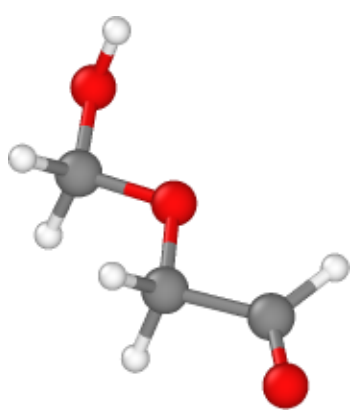

Table S6 List of intended second-step reaction products derived from product 5 of the first step.

	Product geometry		Product geometry		Product geometry		Product geometry
5	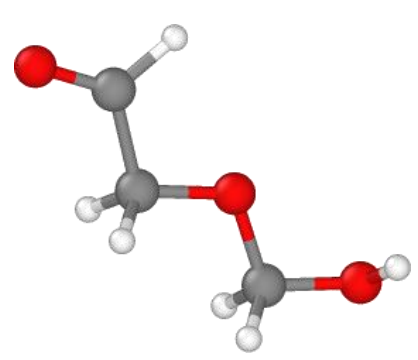	11	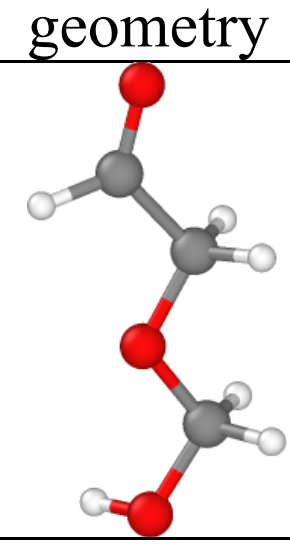	15	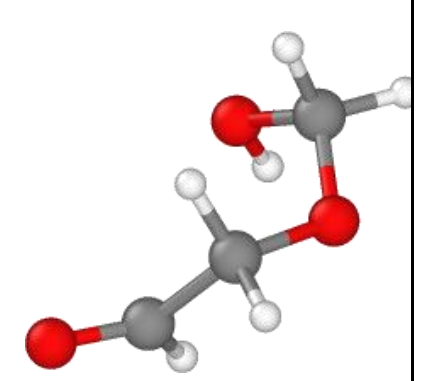		

Reactant: product 24 from the first step:

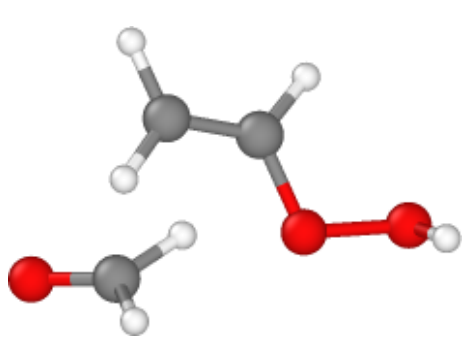

Table S7 List of intended second-step reaction products derived from product 24 of the first step.

	Product geometry		Product geometry		Product geometry		Product geometry
1	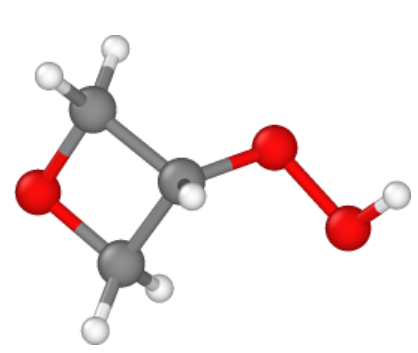	5	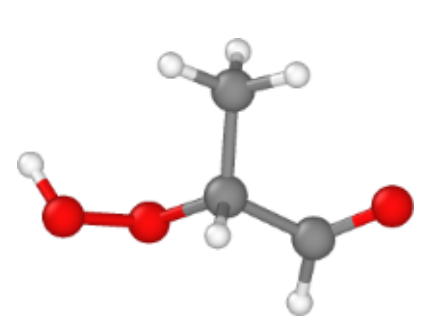	8	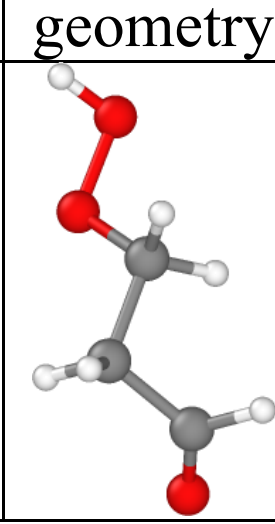	11	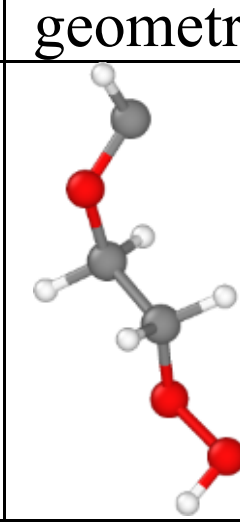

12	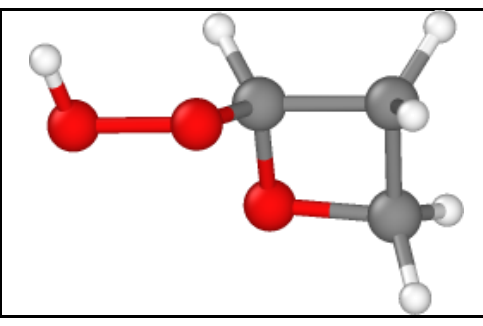						

Reactant: product 25 from the first step:

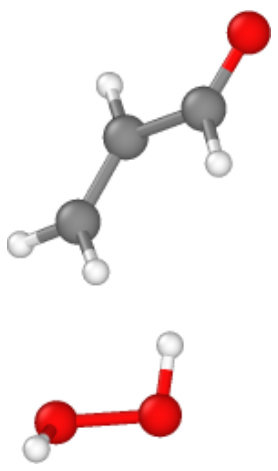

Table S8 List of intended second-step reaction products derived from product 25 of the first step.

	Product geometry		Product geometry		Product geometry		Product geometry
4	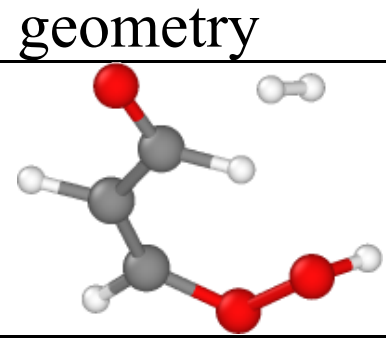	6	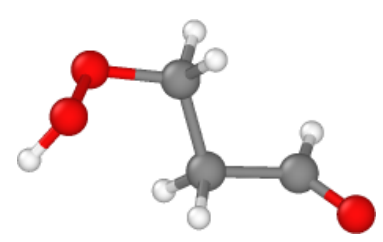				

Reactant: product 30 from the first step:

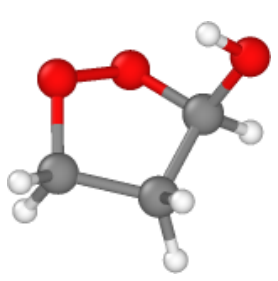

Table S9 List of intended second-step reaction products derived from product 30 of the first step.

	Product geometry		Product geometry		Product geometry		Product geometry
2	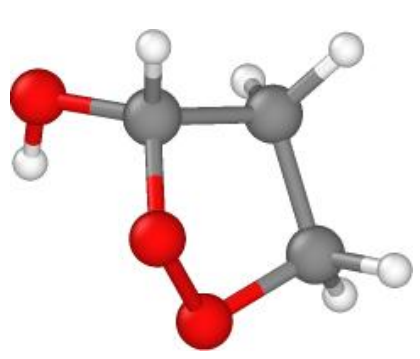	11	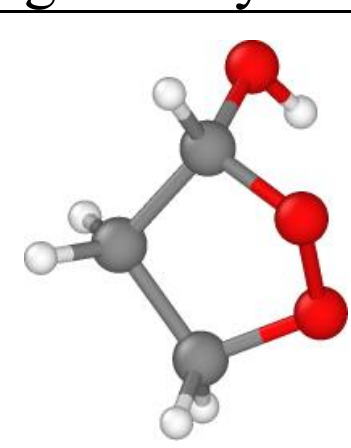	38	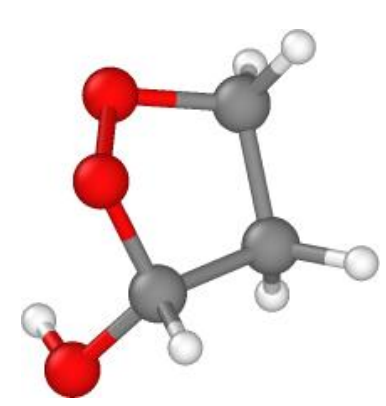	39	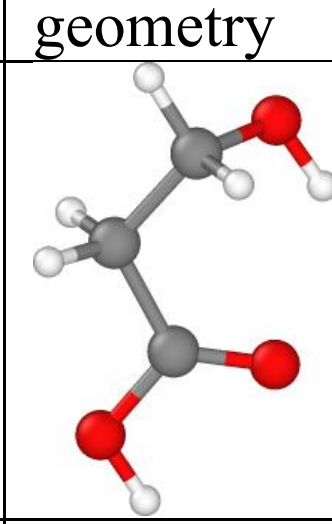
45	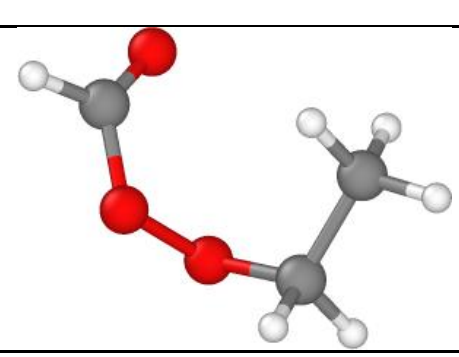						

Reactant: product 36 from the first step:

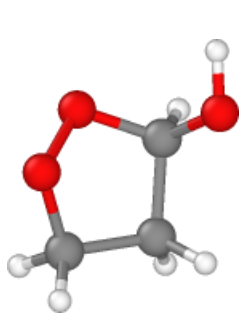

Table S10 List of intended second-step reaction products derived from product 36 of the first step.

	Product geometry		Product geometry		Product geometry		Product geometry
0	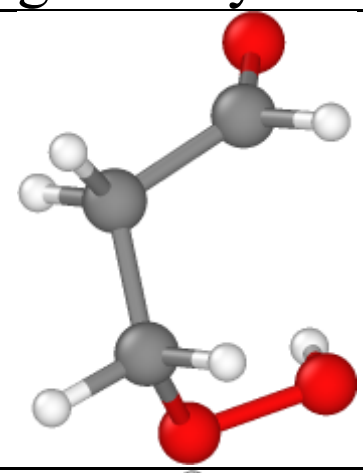	26	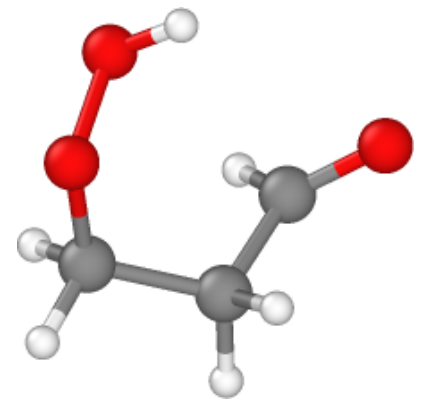	27	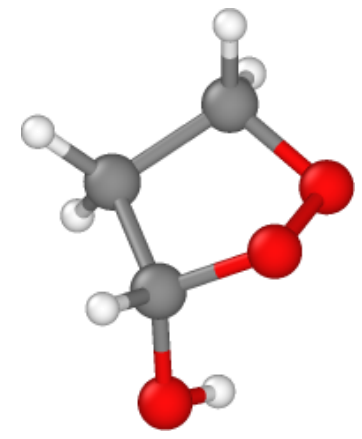	31	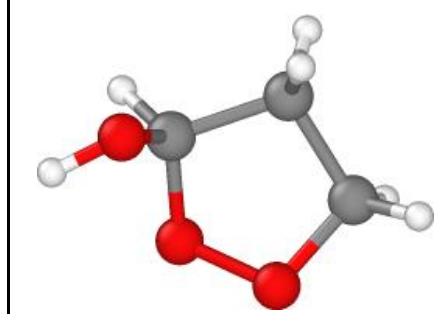
32	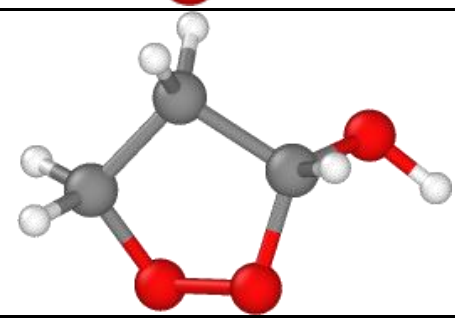						

Reactant: product 37 from the first step:

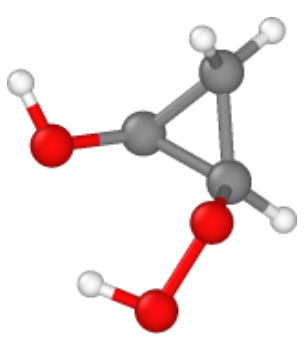

Table S11 List of intended second-step reaction products derived from product 37 of the first step.

	Product geometry		Product geometry		Product geometry		Product geometry
5	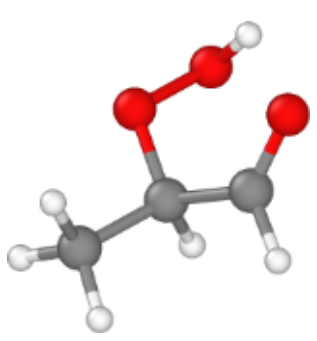	9	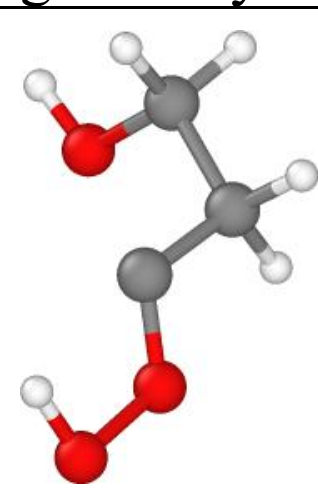	13	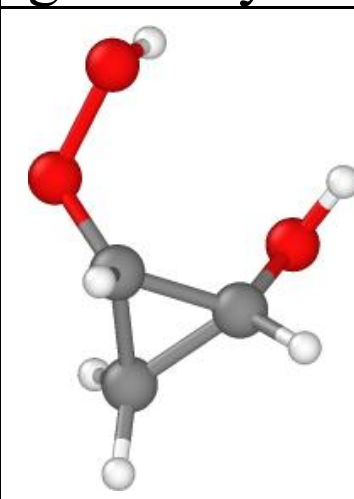	14	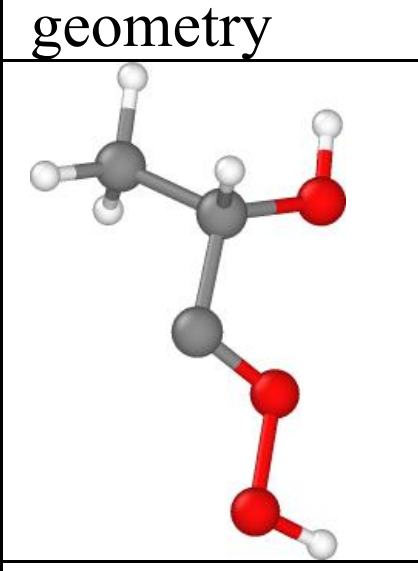
15	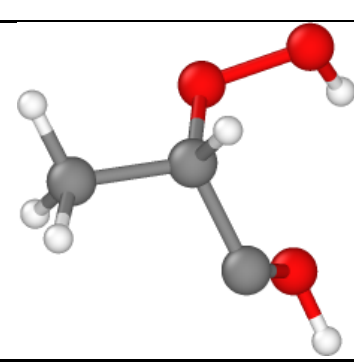	23	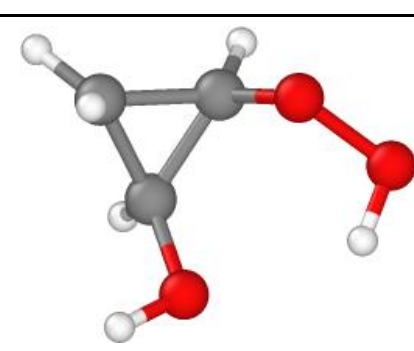	25	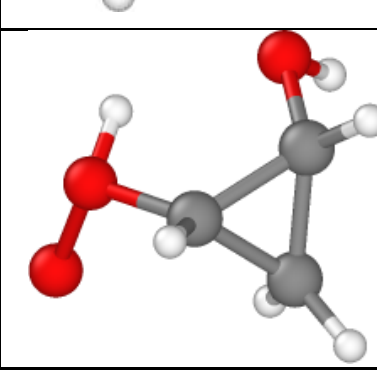		

Reactant: product 54 from the first step:

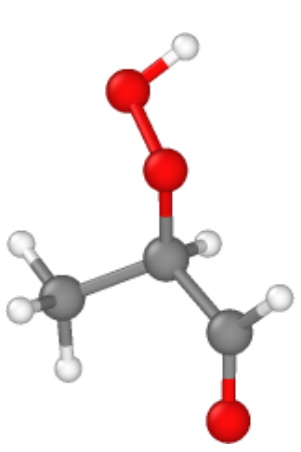

Table S12 List of intended second-step reaction products derived from product 54 of the first step.

	Product geometry
2	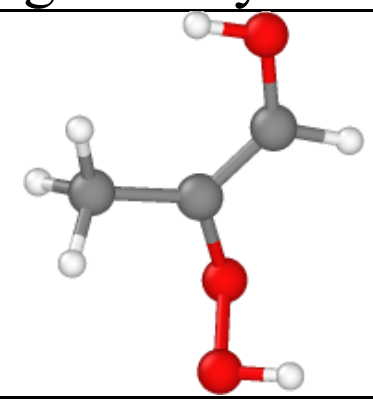

S3.4 Some key observations

- Note that most reactions here have single-molecule products. To increase the ratio of decomposition reactions, one can remove the small molecules (such as the C2H4) from the second-step reactants.
- The improvement in the fraction of intended TSs over YARP, while reasonable, is constrained by the flawed training database, which contains many unintended TSs. This was also reported by Zhao et al. [5] using ReactOT. This underscores the importance of building higher-quality reaction databases.

S4. Model architecture and hyperparameters

S4.1 Hyperparameters for constructing the equivariant messages

A glossary of the shapes of various tensors in the MolGEN architecture:

Table S13 The GNN hyperparameters.

$\mathcal{V}_i$	[N_atoms, N_channels]
$\phi_k(r_{ji})$	[N_edges, N_channels, N_radial_basis]
$w_l(r_{ji})$	[N_edges, N_channels, d_l]
$Y_l(\hat{r}_{ji})$	[N_edges, N_channels, $2l+1$]
$\boldsymbol{\mathcal{M}}(j,i)$	[N_edges, N_channels, $d_0 + d_1 \times 3 + d_2 \times 5$]
$\mathbf{m}(j,i)$	[N_edges, N_channels, $d_0 + d_1 + d_2$]
N_channels	128
N_radial_basis	96
d_0, d_1, d_2	64, 48, 8
l_{max}	2

S4.2 Equivariant transformer model architecture

The equivariant transformer (ET) updates the input equivariant scalar features $H_i \in \mathbb{R}^h$ and vector features $\mathbf{H}_i \in \mathbb{R}^{h\times 3}$ using self-attention (Attn) and Feed-Forward Networks (FFN) with per-layer normalization (LN) and residual connections preceding each operation. In the following, we omit the atom subscript i unless otherwise specified.

The scalar and vector features are iteratively updated by 8 transformer layers:

$$\left[\Delta H^{(l)}, \Delta \mathbf{H}^{(l)}\right] = \mathrm{FFN}\left(\mathrm{LN}\left(\left[H^{(l-1)}, \mathbf{H}^{(l-1)}\right] + \mathrm{Attn}\left(\mathrm{LN}\left(H^{(l-1)}, \mathbf{H}^{(l-1)}\right)\right)\right)\right)$$

$$+\left[H^{(l-1)}, \mathbf{H}^{(l-1)}\right] + \mathrm{Attn}(\mathrm{LN}(H^{(l-1)}, \mathbf{H}^{(l-1)})),$$

where l denote the layer. In the following, we omit the layer superscript l.

Self-attention: For each layer, query Q_s, key K_s, and value $\mathbf{H}_s$ matrices of the s-th head are computed as

$$Q_s = HW_s^Q, K_s = HW_s^K, \mathbf{H}_s = [HW_s^H, \mathbf{H}W_s^{\mathbf{H}}],$$

where, $W_s^Q, W_s^K \in \mathbb{R}^{h\times 4h_s}$, $W_s^H, W_s^{\boldsymbol{H}} \in \mathbb{R}^{h\times h_s}$ are trainable parameters that map the features to the appropriate query, key, and value spaces, and h_s is the dimension of each head. The attention mechanism is given by

$$H_s, \mathbf{H}_s = \mathrm{Softmax}\left(\frac{Q_s^T K_s}{2\sqrt{h_s}} - R + \varphi_r\left(\{\mathbf{e}_{ij}\}_{i,j=1}^N\right)\right)\mathbf{H}_s,$$

where $R = \{r_{ij}\}_{i,j=1}^N = \left\{\left\|\mathbf{r}_i - \mathbf{r}_j\right\|_2\right\}_{i,j=1}^N$ is the distance matrix, $\mathbf{e}_{ij}$ is the edge features and φ_r is an MLP. The outputs of the self-attention layer combine the contributions of all heads:

$$\Delta H = \sum_s H_s W_s^{O_h}, \Delta\mathbf{H} = \mathbf{H}W_s^{O_v},$$

where $W_s^{O_h}, W_s^{O_v} \in \mathbb{R}^{h_s\times h}$ are head-specific trainable parameters.

FFN: The equivariant feed-forward layer is where the scalar and vector features are fused and updated simultaneously:

$$\mathbf{H}_1, \mathbf{H}_2 = \mathbf{H}W_1, \mathbf{H}W_2,$$

$$\Delta H, U = \varphi_{\mathrm{FFN}}(H, \|\mathbf{H}_1\|_2),$$

$$\Delta\mathbf{H} = \mathrm{LN}(U) \odot \mathbf{H}_2,$$

where $W_1, W_2 \in \mathbb{R}^{h\times h}$ are learnable linear projectors, φ_{FFN} is an MLP and $\odot$ denotes element-wise multiplication. The intermediate matrix U is layer-normalized to conserve the scale of the updated vectors.

Hyperparameters of the equivariant transformer:

Table S14 Equivariant transformer hyperparameters.

Name	Value
Number of transformer layers	6
Number of heads	8
h	256
h_s	768
Dimension of φ_r	[256, 256] * 8
Dimension of FFN	[256×2, 768, 256× 2]

References

[1] Schreiner, M., Bhowmik, A., Vegge, T., Busk, J., Winther, O.: Transition1x-a dataset for building generalizable reactive machine learning potentials. Scientific Data 9(1), 779 (2022).

[2] Zhao, Q., Vaddadi, S.M., Woulfe, M., Ogunfowora, L.A., Garimella, S.S., Isayev, O., Savoie, B.M.: Comprehensive exploration of graphically defined reaction spaces. Scientific Data 10(1), 145 (2023).

[3] Duan, C., Liu, G.-H., Du, Y., Chen, T., Zhao, Q., Jia, H., Gomes, C.P., Theodorou, E.A., Kulik, H.J.: Optimal transport for generating transition states in chemical reactions. Nature Machine Intelligence 7(4), 615-626 (2025).

[4] Qiyuan Zhao and Brett M Savoie. Algorithmic explorations of unimolecular and bimolecular reaction spaces. Angewandte Chemie, 134(46):e202210693 (2022).

[5] Qiyuan Zhao, Yunhong Han, Duo Zhang, Jiaxu Wang, Peichen Zhong, Taoyong Cui, Bangchen Yin, Yirui Cao, Haojun Jia, and Chenru Duan. Harnessing machine learning to enhance transition state search with interatomic potentials and generative models. Advanced Science, page e06240 (2025).
