# Supplementary material for: Flow matching for reaction pathway generation
Source: Nat Commun. 2026 Jul 17;17:8769. doi: 10.1038/s41467-026-75654-w (PMC13493859; doi:10.1038/s41467-026-75654-w)
Supplement: Supplementary file 2 [file 41467_2026_75654_MOESM2_ESM.pdf]

nature portfolio

Peer Review File

Flow matching for reaction pathway generation

Corresponding Author: Dr Ping Tuo

This file contains all reviewer reports in order by version, followed by all author rebuttals in order by version.

Version 0:

Reviewer comments:

Reviewer #1

(Remarks to the Author)
This manuscript introduces MolGEN, a conditional flow-matching framework for reaction pathway generation that unifies transition-state (TS) prediction, reaction product generation, and reaction network exploration within a single deterministic optimal-transport formulation. By replacing stochastic diffusion-based sampling with flow matching, the authors achieve substantial improvements in sampling efficiency, controllability, and predictive accuracy, while retaining (and in several cases surpassing) the state-of-the-art performance of diffusion and sequence-based models.

The work is technically rigorous, conceptually elegant, and convincingly validated on multiple benchmark datasets and a realistic reaction-network application. The results demonstrate clear advances over existing approaches, both in terms of scientific insight and practical utility. In my view, this study represents a significant contribution to generative modeling for chemistry and reaction mechanism discovery and fully meets the standards for publication in Nature Communications.

The key conceptual advance of this work is the demonstration that deterministic flow matching can fully replace stochastic diffusion formulations for molecular and reaction generation, while offering decisive advantages in inference speed, controllability, and interpretability. This is not a marginal improvement but a fundamental methodological shift. By framing reaction generation as an optimal-transport problem governed by ODEs, the authors provide a unifying perspective that connects diffusion models, rectified flows, and consistency models within a single framework.

Importantly, the manuscript does not stop at methodological novelty: the authors convincingly show that this formulation leads to tangible gains in TS accuracy, barrier prediction, reaction network exploration efficiency, and robustness against common failure modes such as mass or electron imbalance. The successful application to the γ-ketohydroperoxide decomposition network, including the identification of a lower-barrier pathway than previously reported, highlights the genuine chemical discovery potential of the approach.

In summary, this manuscript presents a substantial and original advance in molecular generative modeling with broad implications for computational chemistry, reaction discovery, and machine-learning-driven mechanism exploration. The methodology is sound, the results are convincing, and the potential impact is high.

I therefore strongly recommend publication in Nature Communications without revision.

(Remarks on code availability)

Reviewer #2

(Remarks to the Author)
reaction upon reading
- in the abstract and in the text, claims are made that diffusion models are harder to control than flow matching models. I don't know of empirical evidence for this and I don't see citations. Furthermore, in the euclidean case, the training objectives are similar to common forms of diffusion (iDEM objective = gaussian Flow Matching objective). Diffusion models are indeed more inefficient at inference time, although this sometime results in higher quality generations. I would encourage the authors to emphasize their network design choices, data curation, use-cases, and hyperparameters instead of the difference between diffusion and flow matching.

- motivation for reaction network enumeration is quite clear and seems to be the main result of the work and the method.
- Equation (2) is not well motivated when it is introduced. After looking at the methods section, I see what you did, but calling it a KL loss seems off given the number of approximations you make in that methods section. I think conditional modified Jensen-Shannon loss would be more appropriate.
- Lines 145-147 could be expanded to explain better how the baseline methods work and specifically what differentiates MolGEN.
- Lines 160-174 indicate all of the nice results from MolGEN's standard generation. Would this also possible be made into a table? It seems more natural here with so many statistics added. Then you can also compare to the baseline methods more clearly. You can use the gained space to explain each metric and refer to the table to explain the performance.
- At the moment, the trends in Table 1 are not very clear to me. Many values are bolded, but no criteria are explained. Why are some of the MolGEN models bolded in columns where they seem to perform worse than other modes? I have a suggestions: Table 1 should have two major sections. In section 1, the models should all have been trained on the same dataset. In section 2, the training data can be adjusted. At the moment using a column to indicate training dataset is confusing.
- On line 180 you mention the introduction of new data using B3LYP-D3/TZVP. This is a significantly less accurate level of theory than in Transition1x. Can you please comment in the text about this difference and the effects it might have on the model? It seems positive you can train on lower quality data and continue to improve results... but would it have been better to train on higher quality data?
- In my opinion, lines 269-293 could be better summarized in a table and referenced more clearly in the text that way.
- In Section 3.2, you introduce an entirely novel way of using MolGEN that does not seem to correspond to Figure 1. Could you please either expand Figure 1 to explain what features you use in your model, or specifically write some equation that explains how predicting products is different from predicting TSes?
- In Section 3.2, you also introduce several benchmark models that seem to simply generate conformers (products)... Is that correct? If so, I'm not sure why those specific models were selected. Aren't there stronger baselines to compare to? I think you should clarify what is necessary in a model to create products.
- Lines 231-238 could also be in table form.
- Section 3.3 is certainly the most interesting part of the paper and one of the primary contributions for this venue. I would suggest making it more clear in the abstract and introduction that this part is so important.
- Lines 284 - 293 are strong results!

structured reply
- The noteworthy results are that the method is able to find novel transition states in unseen systems and seemingly discover new candidates. MolGEN performed well on some benchmarks against a few alternative methods. This technique can be combined with a method for generating products and the corresponding transition states which can compare favorably against other heuristic methods for exploring reaction networks.
- I have a hard time placing the method against others in the field because I am not an expert specifically on transition state generation. I do believe that the method seems novel and quite interesting. In terms of results, my only concern is that the data in question (Transition1x) contains relatively small molecules. I wonder whether transitions states of these systems are particularly interesting when not in contact with another system, e.g. on a catalyst.
- The evidence supports the claims for the most part, but I do believe it can be presented better. I identified above the sections that would be better conveyed in tables or structured data representations (perhaps bar graphs would also work). This would better convince me that the results are significant improvements compared to baselines.
- One major confusion I had on methodology was how the model transitions to generating product states. I could imagine that you simply take the architecture and train it without the concatenated reactant, transition state, and product features... but I don't see where this is made clear. Please explain this part.
- I appreciate that you have shared the code.

(Remarks on code availability)

Reviewer #3

(Remarks to the Author)
This paper introduces a flow-matching framework to generate reaction products and transition states for chemical reactions, which the authors call MolGEN. The framework improves on diffusion-based models when scored on geometry and energy-barrier predictions, while enabling an increase in sampling speed. Particularly, for the prediction of transition-states from reactant-product pairs, MolGEN was able to find faulty entries within the training dataset. The authors also applied their framework to reaction network generation, by repeatedly applying their algorithm on predicted products as new reactants for subsequent steps.

While the framework seems interesting, major improvements and revisions are necessary before I can recommend publication of this article. Additional results are necessary to show if the framework presented here is a significant contribution to the field, as there is too little to currently base a fair assessment on.

Major comments:
The paper lacks good explanations of the model, training and inference procedures and results. Only 2 figures are included, both of which are unclear.
Figure 1 could use a schematic explanation of the full model pipeline (or rather pipelines, if one considers the TS and product prediction as separate instances), as well as better representations about the used data and scoring mechanisms.

The results on TS prediction lack a figure altogether, making it harder to understand the training and prediction mechanisms, as well as interpreting the results. Ideally, true vs predicted plots (or something similar) are shown for the train and test data, as well as examples of the Transition States (both correct and incorrect predictions).
Figure 2 lacks a schematic explanation of the product prediction as well. Additionally, the numeric comparisons to FlowER or Graph2SMILES are only mentioned in the text, and the shown results are very minimal.
The section on Reaction Network Generation requires a figure of its own, both to explain the chosen KHP decomposition network and the methods used to perform the RN generation.
Most importantly, the paper overall lacks sufficient results to back up the claims made. While the comparison to other TS prediction models is nice, the dataset of reactions with only up to 7 atoms is really too limited to make claims about a general-purpose design or flow-matching as the future of molecular generation across chemistry. Similarly for the product predictions. Ideally, not just simulated datasets, but also experimentally verified datasets are included.
The demonstration of RN generation suffers from a lack of clear results, and on second inspection, are not very impressive, barely outperforming single-ended GSM according to fig. 2d. It is unclear whether the predictions are, or can be, experimentally verified. The procedure for RN generation also seems rather subjective, with a lot of intermediate filtering of TSs/products, making the claim of a self-contained and automated framework dubious.
Throughout the paper, references to the Methods and SI are missing, as well as proper explanations of various mechanisms used in this research. It would also be nice to include explanations about the model code, both in the SI and on Github.

Minor comments:
Line 121: where does the velocity field ut(x) come from?
Line 130: 'MolGen demonstrates that flow matching is all that is needed', statement is not backed up by the paper.
Line 165: Characteristic chemical accuracy is ~ 1.58 kcal/mol at 70 C. Why is this specific temperature relevant? Are the datasets for reactions at exactly 70 C, or why is this reference temperature used? Is there a way for the model to include different temperatures and other macroscopic variables in its predictions?
Is the train/validate/test done on a single split, or is multi-fold training used?
It is unclear if the model is re-trained for the product-prediction task, or if the same model as for the TS prediction is used.
For the reaction network generation, why is the prediction of transition states needed at all? Why can you not go from reactants directly to a set of products?
Line 281: 'three radicals were excluded as reaction products.' However, if the model predictions are correct, and radicals are indeed produced, why leave them out from further reaction prediction? In that case, these would seem important to include in the network mechanism.
Line 380: Why is linear change in time a natural choice for probability paths?
Line 386: Why would the final distribution p_1(x|x_1) be a Gaussian?
The Github repository (github.com/tuoping/MolGEN) contains a lot of code that does not relate to the paper at all, and seems to be copied from various ML repositories (DeepMind/Alphafold, Meta/ChemNet, Microsoft)

Overall, I appreciate the interest of a flow-matching framework for molecule prediction, but more results and improved figures are strongly needed to properly assess the framework and warrant a publication in Nature Communications.

(Remarks on code availability)
The code does contain a readme to install and run the training pipeline, but it lacks any explanation about the overall code structure and individual scripts. After looking through the code, I noticed that large parts of the repository contain code not related to the manuscript, but instead were seemingly copied over from various other machine-learning repositories (possibly AlphaFold, ChemNet, perhaps others). It is not necessarily bad to have copied code (and appropriate copyright claims were provided), but there is currently simply no way to properly assess the code related to the MolGen framework. The repository will need to be cleaned up, and documentation needs to be added.

Version 1:

Reviewer comments:

Reviewer #1

(Remarks to the Author)
I have carefully examined the revised version of the manuscript entitled "Flow matching for reaction pathway generation" by Ping Tuo, Jiale Chen, and Ju Li.

The authors have appropriately addressed the comments raised during the first round of review. In particular, the revisions made in response to the other reviewers' suggestions have further strengthened the clarity and presentation of the work.

My overall assessment remains unchanged. I continue to consider this manuscript a significant and original contribution to the field, and I maintain my previous recommendation for publication in Nature Communications without further revision.

(Remarks on code availability)

Reviewer #2

(Remarks to the Author)
- The authors said they changed the name of the loss to modified Jensen-shannon, but I still see "KL div" throughout the paper. Now there is an inconsistency. Please fix this.

If they fix this, I think you had thoughtful responses to my questions. I would support publication.

(Remarks on code availability)

Reviewer #3

(Remarks to the Author)
The authors carefully and appropriately addressed the comments I had, and the presentation of the results and context of this work has much improved.

In my opinion, the figures and some results still need a careful look at:

Fig 1: A schematic overview of the principles of flow networks would help the reader better understand how they are different from diffusion models, and what the two loss functions physically/computationally represent. For fig 1c, the text denoting the various messages and their variables is really too small to read. Additionally, I would like to see which architectural choices have been made regarding the MPNN. The Methods section on this is quite extensive, but it would be useful to the reader to see some of this back in the manuscript.

Fig 2. The cumulative probability of barrier height error is a confusing way of showing the (relative) model performance. Why not just show error histograms, or predicted-vs-true plots for every model? This would allow the reader to more easily assess the relative performance and also the performance for different barrier heights.

Fig 3. a) The Graph2SMILES benchmark mentioned in the previous version is now missing from both the text and the figures. Furthermore, fig 3b and 3c are not mentioned in the text. For 3b I'm not sure why the distribution of product relaxation energy change is relevant. Is this relaxation done after MolGen, or is it performed by MolGen? And if done after MolGen, then why in Fig 2b are the Reference and Generated TS not the same. They look like a non-relaxed and relaxed counterpart (only a slight difference in geometry).

Section 3.3, fig 4. Here, I'm still missing a clear visualization of which products are screened out. There is text in the Methods describing the procedure, but I'm interested to see which exact products are generated and filtered at every step.

Lastly, for a framework focussed on speed vs diffusion methods, I am still missing explicit results on their speed comparison, and how speed and accuracy scale for both flow and diffusion models.

(Remarks on code availability)

Version 2:

Reviewer comments:

Reviewer #3

(Remarks to the Author)
The authors have appropriately addressed my remaining comments, and I support publication in Nature Communications.

(Remarks on code availability)

Open Access This Peer Review File is licensed under a Creative Commons Attribution 4.0 International License, which permits use, sharing, adaptation, distribution and reproduction in any medium or format, as long as you give appropriate credit to the original author(s) and the source, provide a link to the Creative Commons license, and indicate if changes were made.
In cases where reviewers are anonymous, credit should be given to 'Anonymous Referee' and the source.
The images or other third party material in this Peer Review File are included in the article's Creative Commons license, unless indicated otherwise in a credit line to the material. If material is not included in the article's Creative Commons license and your intended use is not permitted by statutory regulation or exceeds the permitted use, you will need to obtain permission directly from the copyright holder.
To view a copy of this license, visit https://creativecommons.org/licenses/by/4.0/

We thank the referees for their examination of our manuscript. The referees' comments helped to further clarify the demonstrations of our method.

In what follows, we address each of the points raised by the referees. The revision of the manuscript in response to the referees' comments is highlighted in red in the main text.

Reviewer #1 (Comments to Author):

This manuscript introduces MolGEN, a conditional flow-matching framework for reaction pathway generation that unifies transition-state (TS) prediction, reaction product generation, and reaction network exploration within a single deterministic optimal-transport formulation. By replacing stochastic diffusion-based sampling with flow matching, the authors achieve substantial improvements in sampling efficiency, controllability, and predictive accuracy, while retaining (and in several cases surpassing) the state-of-the-art performance of diffusion and sequence-based models.

The work is technically rigorous, conceptually elegant, and convincingly validated on multiple benchmark datasets and a realistic reaction-network application. The results demonstrate clear advances over existing approaches, both in terms of scientific insight and practical utility. In my view, this study represents a significant contribution to generative modeling for chemistry and reaction mechanism discovery and fully meets the standards for publication in Nature Communications.

The key conceptual advance of this work is the demonstration that deterministic flow matching can fully replace stochastic diffusion formulations for molecular and reaction generation, while offering decisive advantages in inference speed, controllability, and interpretability. This is not a marginal improvement but a fundamental methodological shift. By framing reaction generation as an optimal-transport problem governed by ODEs, the authors provide a unifying perspective that connects diffusion models, rectified flows, and consistency models within a single framework.

Importantly, the manuscript does not stop at methodological novelty: the authors convincingly show that this formulation leads to tangible gains in TS accuracy, barrier prediction, reaction network exploration efficiency, and robustness against common failure modes such as mass or electron imbalance. The successful application to the γ-ketohydroperoxide decomposition network, including the identification of a lower-barrier pathway than previously reported, highlights the genuine chemical discovery potential of the approach.

In summary, this manuscript presents a substantial and original advance in molecular generative modeling with broad implications for computational chemistry, reaction discovery, and machine-learning-driven mechanism exploration. The methodology is sound, the results are convincing, and the potential impact is high.

I therefore strongly recommend publication in Nature Communications without revision.

Authors:

We thank the reviewer for their time and appreciation of our work.

Reviewer #2 (Comments to Author):

Reaction upon reading 1. in the abstract and in the text, claims are made that diffusion models are harder to control than flow matching models. I don't know of empirical evidence for this and I don't see citations. Furthermore, in the euclidean case, the training objectives are similar to common forms of diffusion (iDEM objective = gaussian Flow Matching objective). Diffusion models are indeed more inefficient at inference time, although this sometime results in higher quality generations. I would encourage the authors to emphasize their network design choices, data curation, use-cases, and hyperparameters instead of the difference between diffusion and flow matching.

Authors:

We thank the referee for the insightful comments and constructive suggestions. The referee raised two main concerns: (1) our claim that diffusion models are harder to control than flow-based models lacked appropriate citations; and (2) while diffusion models are indeed less efficient at inference time, they can sometimes yield higher-quality generations, so slower sampling is not necessarily a weakness. (3) Furthermore, the referee suggested us to emphasize our model design instead of the difference between diffusion and flow matching.

In response to the 1st concern, we rewrite lines 67-69 of the introduction as:

"*However, their reliance on stochastic differential equations (SDEs) introduces limitations familiar to all diffusion approaches: stochastic noise complicates training, slows sampling, and conditional sampling and guidance of diffusion models require complex, task-specific guidance schemes and backpropagation through long stochastic trajectories [18]*

[18] Jiang, R., Zheng, G.-C., Li, T., Yang, T.-R., Wang, J.-D., Li, X.: A survey of multimodal controllable diffusion models. Journal of Computer Science and Technology 39(3), 509–541 (2024)"

In response to the 2nd concern, the "higher-quality generations" referred to in the reviewer's comment are relative to faster/shorter-step diffusion samplers. Our results indicate that the diffusion model requires substantially higher inference cost to achieve comparable quality to the flow-matching model. In our experiments on TS generation, both MolGEN and ReactOT attain better quality than strong diffusion baselines, including TSDiff and OA-ReactDiff, while using significantly fewer function evaluations.

In response to the referee's suggestion, as well as comment 12 and 13, we restructure the abstract and the last paragraph of the introduction to highlight the fully automated reaction network exploration workflow enabled by our model:

Abstract: *Elucidating reaction mechanisms requires efficient generation of transition states (TSs), products, and complete reaction networks. While diffusion models and sequence-based generators accelerate TS and product sampling, respectively, current pipelines still depend on manual enumeration for at least one of these steps. Moreover, the stochastic dynamics underlying diffusion models can be inefficient and difficult to control. We show that flow matching, a deterministic ordinary differ-*

ential equation formulation, can replace stochastic differential equation-based diffusion for reaction generation, and introduce MolGEN, a conditional flow-matching framework that learns an optimal-transport path mapping Gaussian priors to target chemical distributions. On benchmarks used by TSDiff, OA-ReactDiff, and ReactOT, MolGEN improves TS geometry accuracy and barrier-height prediction while enabling sub-second sampling. For product generation, it achieves competitive top-k accuracy and avoids mass/electron-balance violations common to sequence models. Crucially, MolGEN unifies TS and product sampling within a single backbone, enabling reaction network exploration driven by generative inference rather than templates or repeated quantum-chemistry TS searches. In a realistic test on the γ-ketohydroperoxide decomposition network, MolGEN yields more valid/intended TSs than string-based methods while drastically reducing the number of quantum-chemistry evaluations from 1,156 to 12. Furthermore, MolGEN uncovers a novel pathway with a barrier lower than the reported minimum, demonstrating an accurate and efficient framework for generative reaction-network exploration.

Introduction: *Here, we show that flow matching can fully replace diffusion-based formulations for reaction mechanism generation. We introduce MolGEN, a conditional flow-matching framework that generates both TSs and reaction products and, crucially, rolls out multi-step reaction networks using generative inference alone, without templates or hand-crafted rules. To our knowledge, MolGEN is the first end-to-end reaction-network generation framework powered solely by generative models for both TS and product sampling, enabling unified, automated exploration from reactants to complete networks. In Section 3.1, we show that MolGEN halves the TS geometry prediction error of the state-of-the-art diffusion models while reducing sampling time to sub-second inference. Remarkably, it also uncovers alternative transition states with lower barrier heights than those reported in the reference data, underscoring its potential to reveal previously inaccessible mechanistic pathways. In Section 3.2, we evaluate MolGEN on reaction product generation from given reactants, where it attains top-k accuracies comparable to state-of-the-art sequence-based approaches while naturally respecting stoichiometric constraints. Together, these results show that a deterministic flow formulation provides an integrated solution for reaction-network rollout. In Section 3.3, we apply this framework to the decomposition network of γ-ketohydroperoxide (KHP). In this realistic benchmark, MolGEN achieves substantial computational efficiency gains over conventional string-based methods while maintaining high mechanistic fidelity, and it identifies a new decomposition pathway with a barrier lower than the reported minimum. Overall, the combination of high-quality generation, minimal computational cost, and unified TS and product sampling positions flow matching as a compelling paradigm for automated generative reaction network generation.*

2. motivation for reaction network enumeration is quite clear and seems to be the main result of the work and the method.

3. Equation (2) is not well motivated when it is introduced. After looking at the methods section, I see what you did, but calling it a KL loss seems off given the number of approximations you make in that methods section. I think a conditional modified Jensen-Shannon loss would be more

appropriate.

Author:

We thank the referee for this suggestion and fully agree. We now refer to this loss as "*conditional modified Jensen-Shannon divergence loss*" in the revised manuscript.

4. Lines 145-147 could be expanded to explain better how the baseline methods work and specifically what differentiates MolGEN.

Author:

We thank the referee for this suggestion and agree. We now expand lines 145-147 (lines 156-164 of the revised manuscript) by:

"*TSDiff is a diffusion model that generates TS geometries from a Gaussian prior via a stochastic denoising trajectory conditioned on 2D reactant/product graphs. OA-ReactDiff is an object-aware diffusion model that jointly generates reactant (R), TS, and product (P) structures from a Gaussian prior. ReactOT instead formulates TS prediction as an optimal-transport / rectified-flow problem, deterministically mapping the midpoint* $(\boldsymbol{x}_R + \boldsymbol{x}_P)/2$ *to a unique* $\boldsymbol{x}_{TS}$*, where* $\boldsymbol{x}_R, \boldsymbol{x}_P, \boldsymbol{x}_{TS}$ *denote the coordinates of R, P, and TS. In contrast, MolGEN is a flow-matching model that generates TS geometries from a Gaussian prior via a deterministic flow conditioned on R and P.* "

5. Lines 160-174 indicate all of the nice results from MolGEN's standard generation. Would this also possible be made into a table? It seems more natural here with so many statistics added. Then you can also compare to the baseline methods more clearly. You can use the gained space to explain each metric and refer to the table to explain the performance.

Author:

We apologize for the confusion. These results are listed in Table 1. To avoid this confusion, we now add a sentence pointing to Table 1 at the start of the paragraph, in line 175 of the revised manuscript.

6. At the moment, the trends in Table 1 are not very clear to me. Many values are bolded, but no criteria are explained. Why are some of the MolGEN models bolded in columns where they seem to perform worse than other modes? I have a suggestions: Table 1 should have two major sections. In section 1, the models should all have been trained on the same dataset. In section 2, the training data can be adjusted. At the moment using a column to indicate training dataset is confusing.

Author:

We thank the referee for these comments about Table 1. The referee (1) raised concerns that some of the bolded performance of MolGEN seem to be worse than other models; (2) suggested separating the Table into two sections, each trained on the same dataset.

In response to the 1st comment, we clarify that the bold entries in Table 1 originally denoted values tested in this work, whereas the regular (non-bold) entries corresponded to values cited from

literature. We agree that this distinction is redundant, since the cited values are already accompanied by references. Accordingly, we have removed the bold formatting in the revised manuscript. Regarding the cases where the MolGEN models appear to perform worse than other baselines, this is because the reproduction accuracy of MolGEN is indeed close to that of ReactOT, but MolGEN tends to identify alternative TS configurations beyond those in the reference dataset. This point is specifically discussed in lines 189-195.

In response to the 2nd comment, we changed Table 1 according to the referee's suggestion as

Table 1 Summary of statistics for RMSD, barrier height error $|\Delta E|$, ratio of valid TS and ratio of updated TS, where the generated TS have lower energies than the corresponding reference TS by more than $0.1\ \mathrm{kcal/mol}$.

Training and testing database	Model	Loss function	mean RMSD (Å)	mean $\lvert\Delta E\rvert$ (kcal/mol)	Chem. accu. (%)	Ratio of valid TS (%)	Ratio of updated TS (%)
Transition1x[a] Grambow[b]	TSDiff	KL div	0.253[a]	10.369[a]	10.9[a]	97.00[b]	30.96[b]
Transition1x	OA-ReactDiff +recommender	KL div	0.129[a]	4.453[a]	48.7[a]		
Transition1x	React-OT	FM	0.103[a]	3.337[a]	63.6[a]		
Transition1x	MolGEN	FM & finetune by KL div	0.106	3.285	54.38	87.46	7.32
Transition1x (Pretrained by RGD1-xTB)	React-OT +pretraining	FM	0.088<br>0.098[a]	3.608<br>2.864[a]	57.0<br>71.9[a]	74.67	4.00
RGD1+ Transition1x	MolGEN	FM & finetune by KL div	0.100	2.759	70.80	89.20	27.87
RGD1+ Transition1x	MolGEN	KL div	0.112	2.844	68.98	88.50	30.66

Notes: React-OT was evaluated using the same pretrained model provided by the original authors.

[a] Values from Duan [26], trained/tested on Transition1x [21].

[b] Values from Kim [16], trained/tested on the Grambow dataset [27].

7. On line 180 you mention the introduction of new data using B3LYP-D3/TZVP. This is a

significantly less accurate level of theory than in Transition1x. Can you please comment in the text about this difference and the effects it might have on the model? It seems positive you can train on lower quality data and continue to improve results... but would it have been better to train on higher quality data?

Author:

We thank the referee for this insightful comment. The referee pointed out that the RGD1 and Transition1x data are computed at different levels of DFT, and suggested commenting in the text about this difference and the effects it might have on the model.

In response, we add a paragraph after the introduction of the RGD1 data, in lines 208-218, commenting on this difference:

"*Although RGD1 and Transition1x are computed at different levels of DFT, the resulting TS geometries are typically within* $\sim 0.05 - 0.1$ Å *of each other in RMSD for the same reaction [29-31]. This difference is comparable to the current predictive uncertainty of MolGEN* (~ 0.1 Å)*, so the functional mismatch mainly acts as a mild label noise rather than inducing qualitatively different structures. In practice, the additional RGD1 reactions substantially increase the diversity of TS topologies and local bonding patterns seen during training, which appears to outweigh the modest decrease in electronic-structure fidelity, leading to overall improved generalization. This suggests that generative models can be trained on relatively inexpensive DFT data without compromising performance, while further improvements may be achieved by incorporating higher-quality data.*

[29] Lynch, B.J., Truhlar, D.G.: How well can hybrid density functional methods predict transition state geometries and barrier heights? The Journal of Physical Chemistry A 105(13), 2936–2941 (2001)

[30] Heinen, S., Rudorff, G.F., Lilienfeld, O.A.: Transition state search and geometry relaxation throughout chemical compound space with quantum machine learning. The Journal of Chemical Physics 157(22) (2022)

[31] Beaglehole, I.W., Pemberton, M.J., Farrar, E.H., Grayson, M.N.: Machine learning transition state geometries and applications in reaction property prediction. Wiley Interdisciplinary Reviews: Computational Molecular Science 15(3), 70025 (2025) "

8. In my opinion, lines 269-293 could be better summarized in a table and referenced more clearly in the text that way.

Author:

We thank the referee for this suggestion and fully agree. The generated first-step reaction of KHP decomposition by MolGEN and YARP was originally summarized in a table in the Supplementary Section 3.1. In response to the referee's suggestion, we now add a table in the main text and refer to it in line 322 of the revised manuscript.

Table 2 Comparison of the first step of KHP decomposition reaction network generated by MolGEN and by YARP [37].

	Num. of unique reactions	Num. of intended unique reactions
YARP	20	13
MolGEN	59	14

9. In Section 3.2, you introduce an entirely novel way of using MolGEN that does not seem to correspond to Figure 1. Could you please either expand Figure 1 to explain what features you use in your model, or specifically write some equation that explains how predicting products is different from predicting TSes?

Author:

We thank the referee for this question. The referee raised concerns that the pipeline for product generation used in Section 3.2 is not illustrated in Figure 1, and suggested expanding Figure 1 to explain the features used in the model.

To better illustrate the pipelines used in this work, both for TS generation in Section 3.1 and for product generation Section 3.2, we replot Figure 1 as shown below.

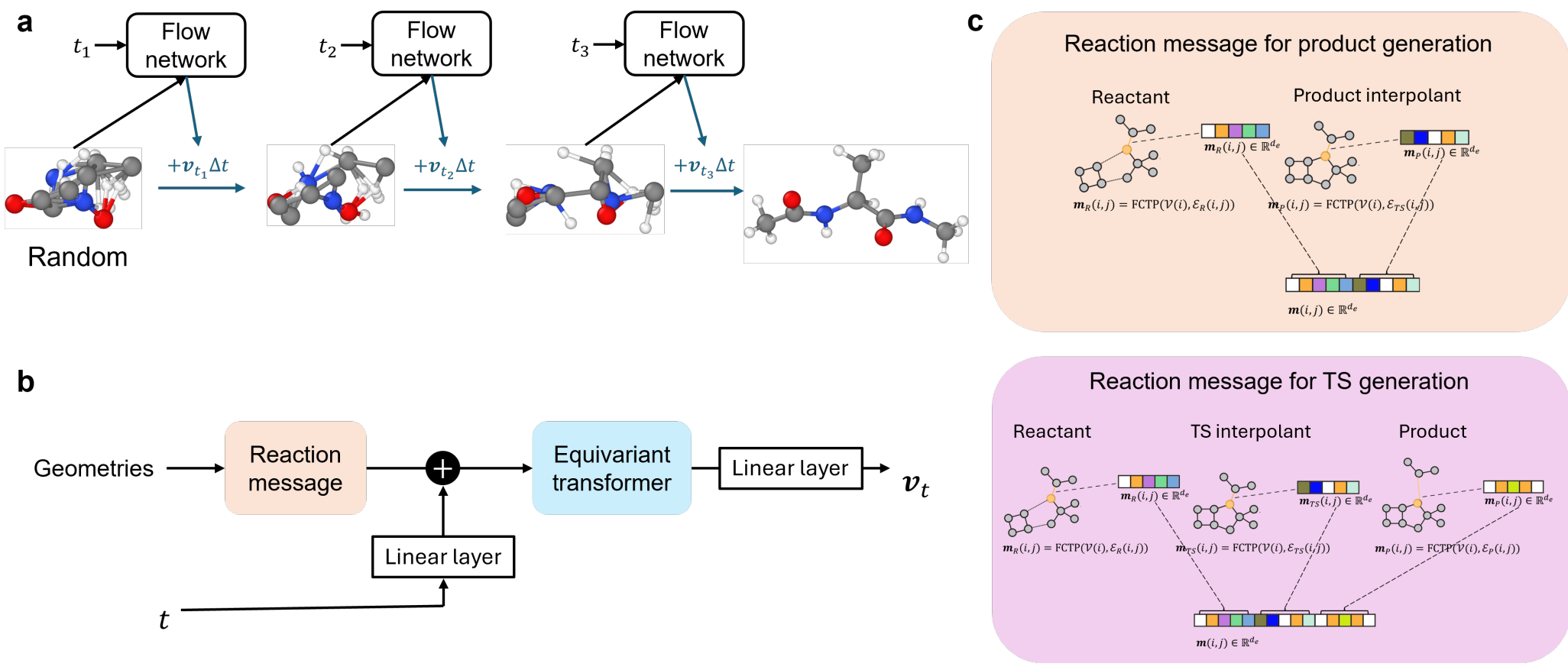

Fig. 1 Overview of MolGEN. a. Schematic illustration of the flow matching workflow. The reactant (or reactant and product) geometry and a randomly initialized product (or TS) geometry are fed into a flow network, which predicts a velocity field ("flow"). This field is then applied iteratively to the random geometry, transforming it into the target product (or TS). **b.** A schematic of the MPNN flow model. The workflow takes the input geometries (e.g., the reactant and the product interpolant) and encodes them into a reaction message. In parallel, the scalar integration time t is passed through a linear layer to produce a time embedding, which is added to the reaction messages and then processed by an SE(3)-equivariant transformer, and a final linear layer outputs the flow v_t, which iteratively updates the product interpolant. **c.** Illustration of the reaction messages. For product generation, the molecular messages of the reactant $\boldsymbol{m}_R(i,j)$ is computed from its geometry, while the product message $\boldsymbol{m}_P(i,j)$ is obtained from the flow-matching interpolants. The overall reaction message, $\boldsymbol{m}(i,j)$, is constructed by concatenating $\boldsymbol{m}_R(i,j)$ and $\boldsymbol{m}_P(i,j)$. The reaction message for TS generation is constructed similarly by concatenating $\boldsymbol{m}_R(i,j)$ and $\boldsymbol{m}_P(i,j)$ constructed from the corresponding geometries, and $\boldsymbol{m}_{\mathrm{TS}}(i,j)$ constructed from the TS interpolants.

10. In Section 3.2, you also introduce several benchmark models that seem to simply generate conformers (products)... Is that correct? If so, I'm not sure why those specific models were selected. Aren't there stronger baselines to compare to? I think you should clarify what is necessary in a model to create products.

We thank the reviewer for the opportunity to clarify this point. The benchmark models used in Section 3.2, Graph2SMILES and FlowER, do not merely generate conformers of a fixed connectivity; they are the state-of-the-art reaction product prediction models that take reactant SMILES as input and output new molecular SMILES strings corresponding to products. That is, they explicitly change bond connectivity and, in the case of FlowER, model electron flow along the reaction.

We chose Graph2SMILES and FlowER for three reasons:

1) Strength and relevance. Both are state-of-the-art, template-free models for single-step reaction product prediction on standard benchmarks (e.g. USPTO). They represent the two main classes

of modern data-driven baselines in this space: a generic sequence-to-sequence SMILES predictor (Graph2SMILES) and an electron-distribution–aware model (FlowER).

2) Task alignment. Our product task in Section 3.2 is to predict the set of possible products of an elementary step given the reactant; Graph2SMILES and FlowER were designed precisely for this type of reactant $\rightarrow$ product SMILES mapping.

Regarding the question of "what is necessary in a model to create products", for the elementary-step product prediction task considered here, a suitable model must: (i) take as input the reactant (and optionally conditions), (ii) generate one or more new molecular graphs representing possible products, (iii) respect fundamental constraints such as atom conservation and chemically reasonable valence, and (iv) support multi-valued mappings (multiple plausible products for the same reactant). Graph2SMILES and FlowER satisfy these criteria in SMILES space, while MolGEN satisfies them in 3D coordinate space.

To clear this up, we add the following comment in lines 265-266 of the revised manuscript:

"*We compare MolGEN's top-k accuracy against that of a state-of-the-art sequence model with electron distribution encoding, FlowER, which takes reactant SMILES as input and outputs product SMILES strings.*"

11. Lines 231-238 could also be in table form.

Author:

We thank the referee for this constructive suggestion. To better present the product generation results in lines 231-238. We dedicated a new Figure (Figure 3 of the revised manuscript) to product generation results. In the new Figure 3a, we compare the top-k step accuracy of MolGEN and FlowER on the Transition1x test set, and discuss the results in lines 269-270 of the revised manuscript:

"*The results are shown in Figure 3a, where MolGEN achieves near 100% top-3 (and larger k) accuracy. (The top-k accuracy of FlowER in Figure 3a is $< 50\%$, significantly lower than that of MolGEN and the value reported by Joung et al. [34]. We speculate that this is because the FlowER parameters require careful tuning and are more sensitive to the faulty data in the Transition1x database.)*

[34] Joung, J.F., Fong, M.H., Casetti, N., Liles, J.P., Dassanayake, N.S., Coley, C.W.:Electron flow matching for generative reaction mechanism prediction. Nature 645,115–123 (2025) "

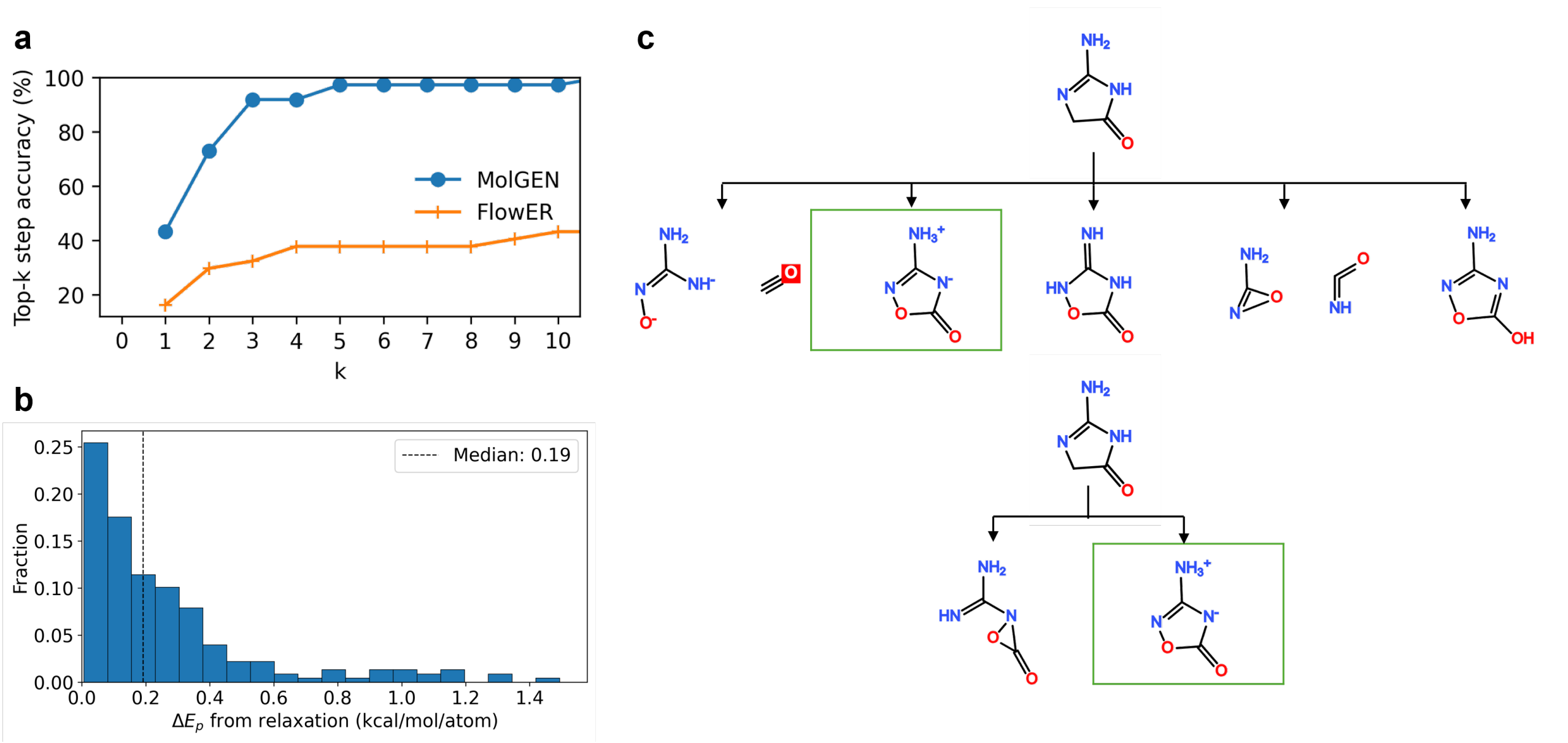

Fig. 3 a. Top-k step accuracy for product prediction of a single elementary reaction. **b.** Energy change of the transition state (ΔE_{TS}) following structural relaxation. **c.** Representative product prediction example, with reference reactions shown on top and the corresponding predictions shown below.

12. Section 3.3 is certainly the most interesting part of the paper and one of the primary contributions for this venue. I would suggest making it more clear in the abstract and introduction that this part is so important.

Author:

We thank the referee for this suggestion. To highlight the strong result that MolGEN provides a framework that substantially automates reaction network exploration, and managed to identify a previously unreported KHP decomposition pathway with a lower barrier than the reported minimum, we rewrite the abstract and the introduction of the revised manuscript, as explained in the response to Q1.

13. Lines 284 - 293 are strong results!

structured reply - The noteworthy results are that the method is able to find novel transition states in unseen systems and seemingly discover new candidates. MolGEN performed well on some benchmarks against a few alternative methods. This technique can be combined with a method for generating products and the corresponding transition states which can compare favorably against other heuristic methods for exploring reaction networks.

- I have a hard time placing the method against others in the field because I am not an expert specifically on transition state generation. I do believe that the method seems novel and quite interesting. In terms of results, my only concern is that the data in question (Transition1x) contains relatively small molecules. I wonder whether transitions states of these systems are particularly

interesting when not in contact with another system, e.g. on a catalyst.

Author:

We thank the referee for these insightful comments. The referee raised two points: (1) they find it hard to place the method against others in the field; (2) they raised concerns about the relevance of small-molecule TS data such as Transition1x.

In response to the 1st point, in our manuscript, we benchmark MolGEN against the state-of-the-art models for each subtask: TSDiff, OA-ReactDiff, and ReactOT for TS generation; sequence-based model FlowER for product generation; and string-based YARP framework for reaction network exploration. So we agree that a single "full-process" baseline is not available in the literature. However, we see this as precisely one of the main contributions of our work. Previous approaches are specialized to individual tasks, whereas MolGEN is, to our knowledge, the first model that simultaneously achieves competitive performance on both TS and product generation and, in doing so, enables an integrated, fully generative workflow for automated reaction network exploration. To better convey this point, we have rewritten the abstract and introduction as explained in the response to Q1.

In response to the 2nd point, we thank the referee for pointing out this limitation. We agree that TS benchmarks are indeed restricted to relatively small molecules, primarily because high-level electronic-structure calculations for transition states remain computationally demanding. This is why many prior TS datasets (including Transition1x and RGD1) and models (including TSDiff, OA-ReactDiff, ReactOT) are also constructed from small organic reactions.

At the same time, we believe that small-molecule TSs are still scientifically meaningful: they capture fundamental elementary steps such as bond dissociation/formation, proton transfers, radical abstractions, and rearrangements that also occur in catalytic contexts (e.g. on surfaces or in homogeneous catalyst coordination spheres). Notably, MolGEN's architecture does not assume gas-phase conditions; in principle, the same conditional flow-matching framework can incorporate additional conditioning variables (e.g. explicit catalyst fragments, surface slabs, or learned descriptors of the environment) once suitable TS datasets that include these effects become available.

- The evidence supports the claims for the most part, but I do believe it can be presented better. I identified above the sections that would be better conveyed in tables or structured data representations (perhaps bar graphs would also work). This would better convince me that the results are significant improvements compared to baselines.

Author:

We thank the referee for all the constructive suggestions. To better convey our results, we have made the following modifications in the revised manuscript based on the suggestions given above:

For TS generation, our results show that MolGEN surpasses the state-of-the-art generative models (such as TSDiff, OA-ReactDiff, and ReactOT) in accuracy, while managing to locate alternative

TS with lower barriers. To improve the presentation:

1) We rearrange Table 1 according to the referee's suggestion and split the table into two parts, each trained on the same dataset, as described in the response to Q6.

2) We add a new Figure (Figure 2 in the revised manuscript) and show a comparison among the performances of various models.

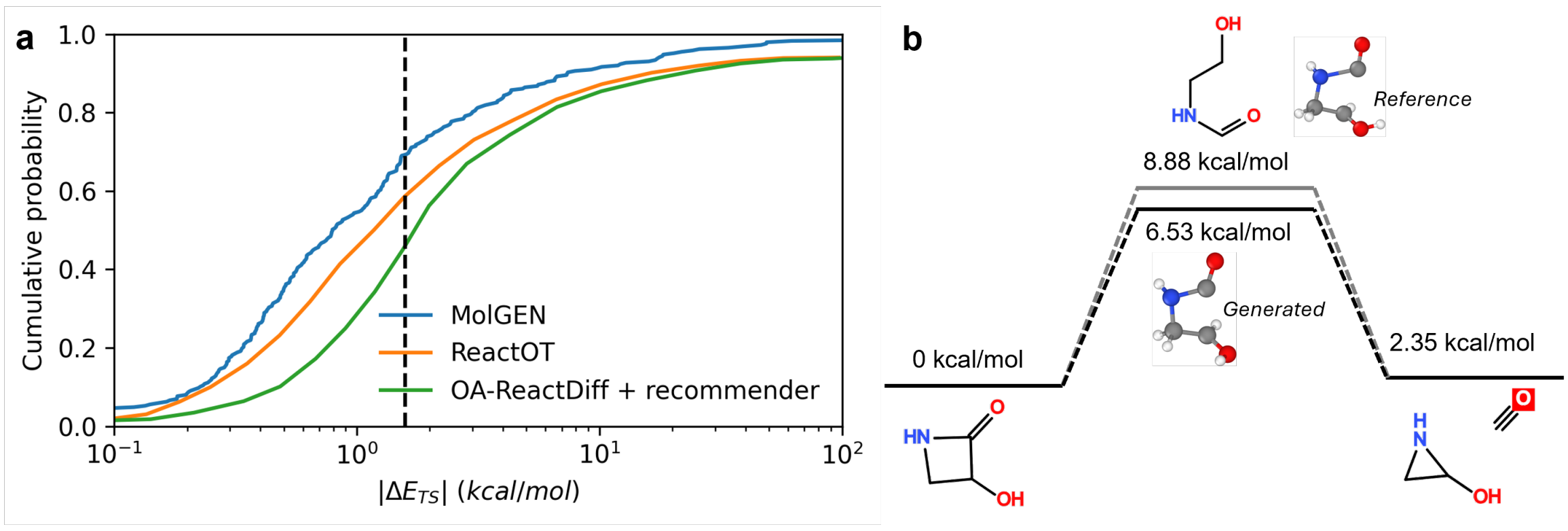

Fig. 2 a. Cumulative probability of the barrier height error. The ReactOT and OA-ReactDiff results are reproduced from Reference [26] and [17], respectively. **b.** An example transition state (TS) generated by MolGEN, with a lower barrier than the reference.

For product generation, our results show that MolGEN can predict reaction products with high top-k accuracy while avoiding mass/electron-balance violations common to the state-of-the-art sequence models. To improve the presentation:

3) We add a new Figure (Figure 3 in the revised manuscript) and compare the top-k step accuracy of MolGEN and FlowER on the Transition1x test set, as described in the response to Q11.

For reaction network exploration, MolGEN provides the first unified generative model-driven framework that substantially automates reaction network exploration, and manages to identify a new KHP decomposition pathway with a lower barrier than previously reported. To improve the presentation:

4) We add Table 2 to summarize the comparison between the performance of MolGEN and YARP in KHP decomposition reaction network exploration, as described in the response to Q8.

5) We rewrite the abstract and introduction to highlight this result.

- One major confusion I had on methodology was how the model transitions to generating product states. I could imagine that you simply take the architecture and train it without the concatenated reactant, transition state, and product features... but I don't see where this is made clear. Please explain this part.

Author:

We thank the referee for this detailed question. As described in the response to Q9, we replot

Figure 1 to better illustrate the pipelines for product generation and TS generation in the revised manuscript.

- I appreciate that you have shared the code.

Reviewer #3 (Comments to Author):

This paper introduces a flow-matching framework to generate reaction products and transition states for chemical reactions, which the authors call MolGEN. The framework improves on diffusion-based models when scored on geometry and energy-barrier predictions, while enabling an increase in sampling speed. Particularly, for the prediction of transition-states from reactant-product pairs, MolGEN was able to find faulty entries within the training dataset. The authors also applied their framework to reaction network generation, by repeatedly applying their algorithm on predicted products as new reactants for subsequent steps.

While the framework seems interesting, major improvements and revisions are necessary before I can recommend publication of this article. Additional results are necessary to show if the framework presented here is a significant contribution to the field, as there is too little to currently base a fair assessment on.

Major comments: 1. The paper lacks good explanations of the model, training and inference procedures and results. Only 2 figures are included, both of which are unclear. Figure 1 could use a schematic explanation of the full model pipeline (or rather pipelines, if one considers the TS and product prediction as separate instances), as well as better representations about the used data and scoring mechanisms.

Author:

We thank the referee for these constructive suggestions. The referee suggested (1) a schematic explanation of the full model pipeline for both TS and product prediction in Figure 1; (2) better representations about the used data and scoring mechanisms.

In response to suggestion (1), to better illustrate the pipelines used in this work, both for TS generation and for product generation, we replot Figure 1 as shown below.

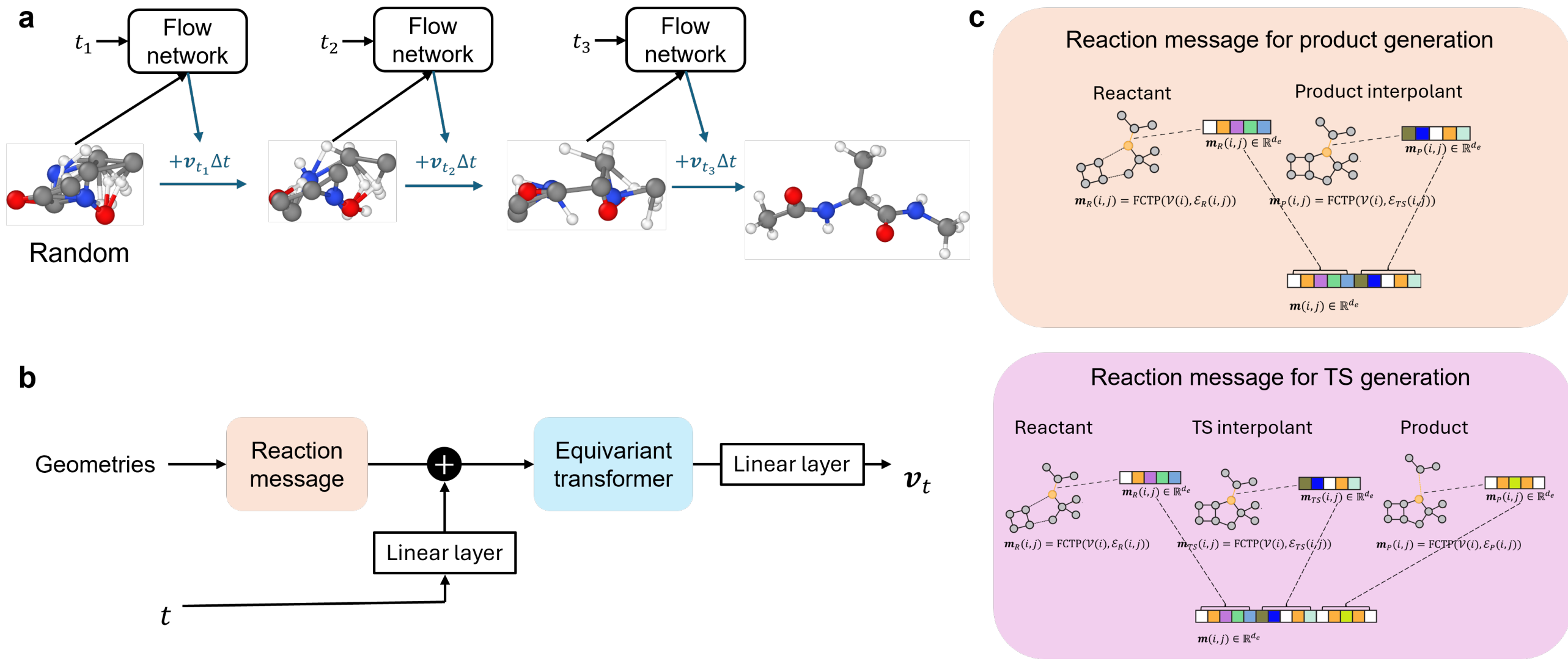

Fig. 1 Overview of MolGEN. a. Schematic illustration of the flow matching workflow. The reactant (or reactant and product) geometry and a randomly initialized product (or TS) geometry are fed into a flow network, which predicts a velocity field ("flow"). This field is then applied iteratively to the random geometry, transforming it into the target product (or TS). **b.** A schematic of the MPNN flow model. The workflow takes the input geometries (e.g., the reactant and the product interpolant) and encodes them into a reaction message. In parallel, the scalar integration time t is passed through a linear layer to produce a time embedding, which is added to the reaction messages and then processed by an SE(3)-equivariant transformer, and a final linear layer outputs the flow v_t, which iteratively updates the product interpolant. **c.** Illustration of the reaction messages. For product generation, the molecular messages of the reactant $\boldsymbol{m}_R(i,j)$ is computed from its geometry, while the product message $\boldsymbol{m}_P(i,j)$ is obtained from the flow-matching interpolants. The overall reaction message, $\boldsymbol{m}(i,j)$, is constructed by concatenating $\boldsymbol{m}_R(i,j)$ and $\boldsymbol{m}_P(i,j)$. The reaction message for TS generation is constructed similarly by concatenating $\boldsymbol{m}_R(i,j)$ and $\boldsymbol{m}_P(i,j)$ constructed from the corresponding geometries, and $\boldsymbol{m}_{\mathrm{TS}}(i,j)$ constructed from the TS interpolants.

In response to suggestion (2), to improve the presentation of the results, we make the following major revisions:

1) **For TS generation,** to improve clarity, we rearrange Table 1 and split the table into two parts, each trained on the same dataset, in response to Q2.

2) **For TS generation,** we add a new Figure (Figure 2 in the revised manuscript) of examples TS configurations generated by MolGEN, in response to Q2.

3) **For product generation,** we add a new Figure (Figure 3 in the revised manuscript) and compare the top-k step accuracy of MolGEN and FlowER on the Transition1x test set, in response to Q3.

4) **For reaction network exploration,** we add a new Table 2 to summarize the comparison between the performance of MolGEN and YARP on KHP decomposition reaction network exploration.

2. The results on TS prediction lack a figure altogether, making it harder to understand the training and prediction mechanisms, as well as interpreting the results. Ideally, true vs predicted plots (or something similar) are shown for the train and test data, as well as examples of the Transition States (both correct and incorrect predictions).

Author:

We thank the referee for these constructive suggestions. To better illustrate the results on TS prediction, we make two major modifications in the revised manuscript:

1) We add a new Figure (Figure 2 in the revised manuscript) and show a comparison among the performances of various models.

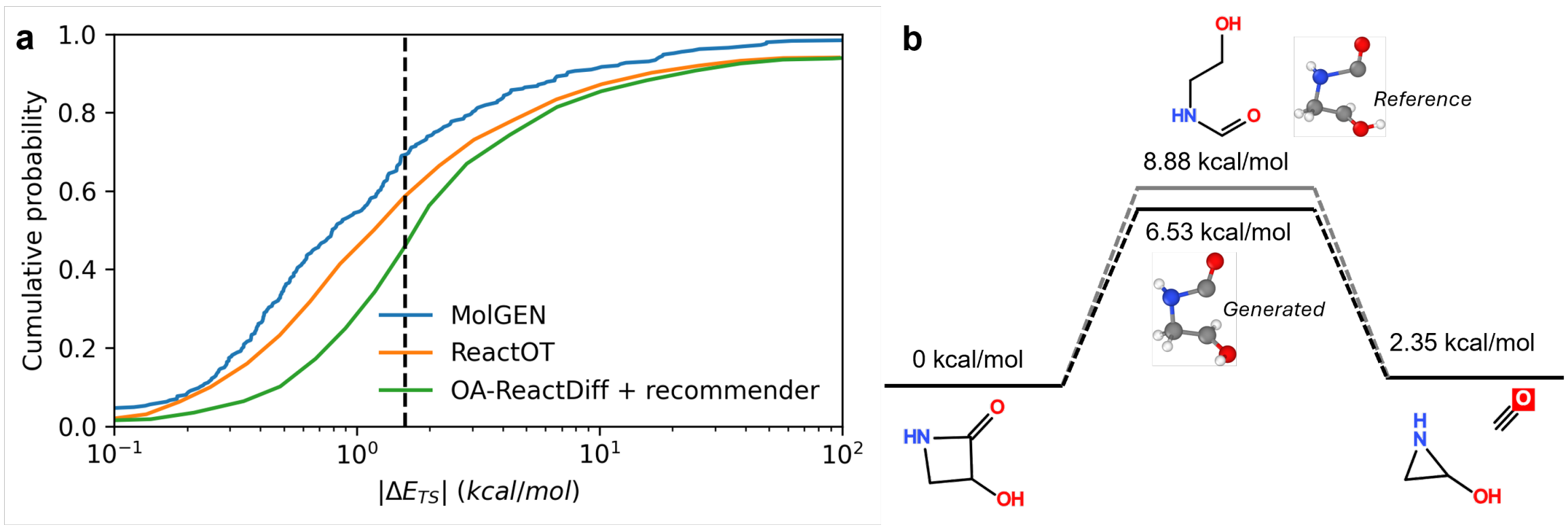

Fig. 2 a. Cumulative probability of the barrier height error. The ReactOT and OA-ReactDiff results are reproduced from Reference [26] and [17], respectively. **b.** An example transition state (TS) generated by MolGEN, with a lower barrier than the reference.

2) To compare the performance of different models more clearly, we rearrange Table 1 and split the table into two parts, each trained on the same dataset.

Table 1 Summary of statistics for RMSD, barrier height error $|\Delta E|$, ratio of valid TS and ratio of updated TS, where the generated TS have lower energies than the corresponding reference TS by more than 0.1 kcal/mol.

Training and testing database	Model	Loss function	mean RMSD (Å)	mean $\lvert\Delta E\rvert$ (kcal/mol)	Chem. accu. (%)	Ratio of valid TS (%)	Ratio of updated TS (%)
Transition1x[a] Grambow[b]	TSDiff	KL div	0.253[a]	10.369[a]	10.9[a]	97.00[b]	30.96[b]
Transition1x	OA-ReactDiff +recommender	KL div	0.129[a]	4.453[a]	48.7[a]		
Transition1x	React-OT	FM	0.103[a]	3.337[a]	63.6[a]		
Transition1x	MolGEN	FM & finetune by KL div	0.106	3.285	54.38	87.46	7.32
Transition1x (Pretrained by RGD1-xTB)	React-OT +pretraining	FM	0.088<br>0.098[a]	3.608<br>2.864[a]	57.0<br>71.9[a]	74.67	4.00
RGD1+ Transition1x	MolGEN	FM & finetune by KL div	0.100	2.759	70.80	89.20	27.87
RGD1+ Transition1x	MolGEN	KL div	0.112	2.844	68.98	88.50	30.66

Notes: React-OT was evaluated using the same pretrained model provided by the original authors.

[a] Values from Duan [26], trained/tested on Transition1x [21].

[b] Values from Kim [16], trained/tested on the Grambow dataset [27].

3. Figure 2 lacks a schematic explanation of the product prediction as well. Additionally, the numeric comparisons to FlowER or Graph2SMILES are only mentioned in the text, and the shown results are very minimal.

Author:

We thank the referee for pointing out (1) the lack of a schematic explanation of the product prediction; (2) the shown comparison results to FlowER and Graph2SMILES are minimal.

In response to the 1st remark, as described in the response to Q1, we replot Figure 1 to better illustrate the model pipeline.

In response to the 2nd remark, we thank the referee for pointing out that the numeric comparisons to FlowER and Graph2SMILES were only briefly mentioned in the text. To better present the product generation results. We dedicated a new Figure (Figure 3 of the revised manuscript) to product generation results. In the new Figure 3a, we compare the top-k step accuracy of MolGEN and FlowER on the Transition1x test set, and discuss the results in lines 266-271 of the revised manuscript:

"*The results are shown in Figure 3a, where MolGEN achieves near 100% top-3 (and larger k) accuracy. (The top-k accuracy of FlowER in Figure 3 is $< 50\%$, significantly lower than that of MolGEN and the value reported by Joung et al. [34]. We speculate that this is because the FlowER parameters require careful tuning and are more sensitive to the faulty data in the Transition1x database.)*

[34] Joung, J.F., Fong, M.H., Casetti, N., Liles, J.P., Dassanayake, N.S., Coley, C.W.:Electron flow matching for generative reaction mechanism prediction. Nature 645,115–123 (2025) "

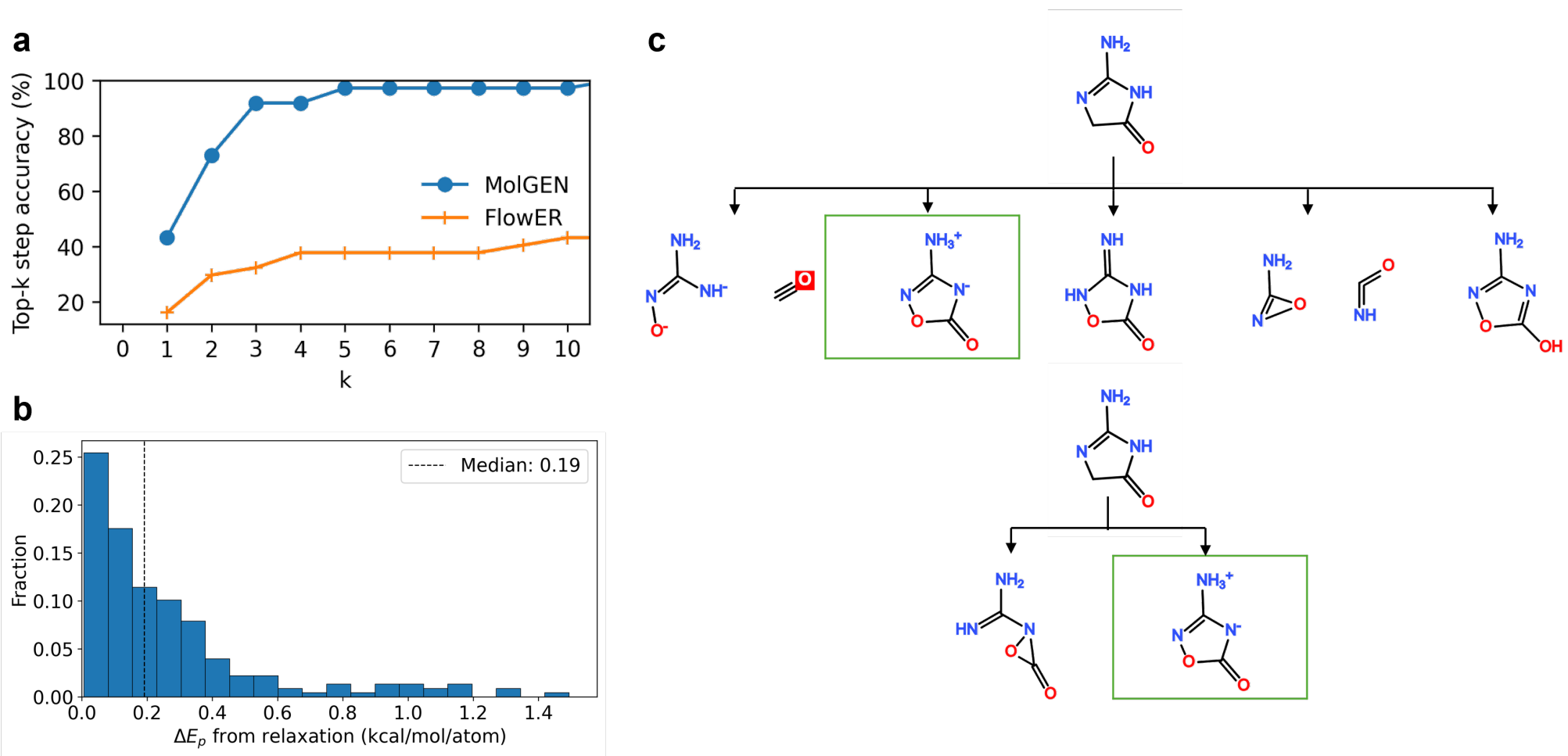

Fig. 3 a. Top-k step accuracy for product prediction of a single elementary reaction. **b.** Energy change of the transition state (ΔE_{TS}) following structural relaxation. **c.** Representative product prediction example, with reference reactions shown on top and the corresponding predictions shown below.

4. The section on Reaction Network Generation requires a figure of its own, both to explain the chosen KHP decomposition network and the methods used to perform the RN generation.

Author:

We thank the referee for this constructive suggestion. We now dedicate Figure 4 of the revised manuscript only to KHP decomposition network exploration, and illustrate the workflow of reaction network exploration in Figure 4a.

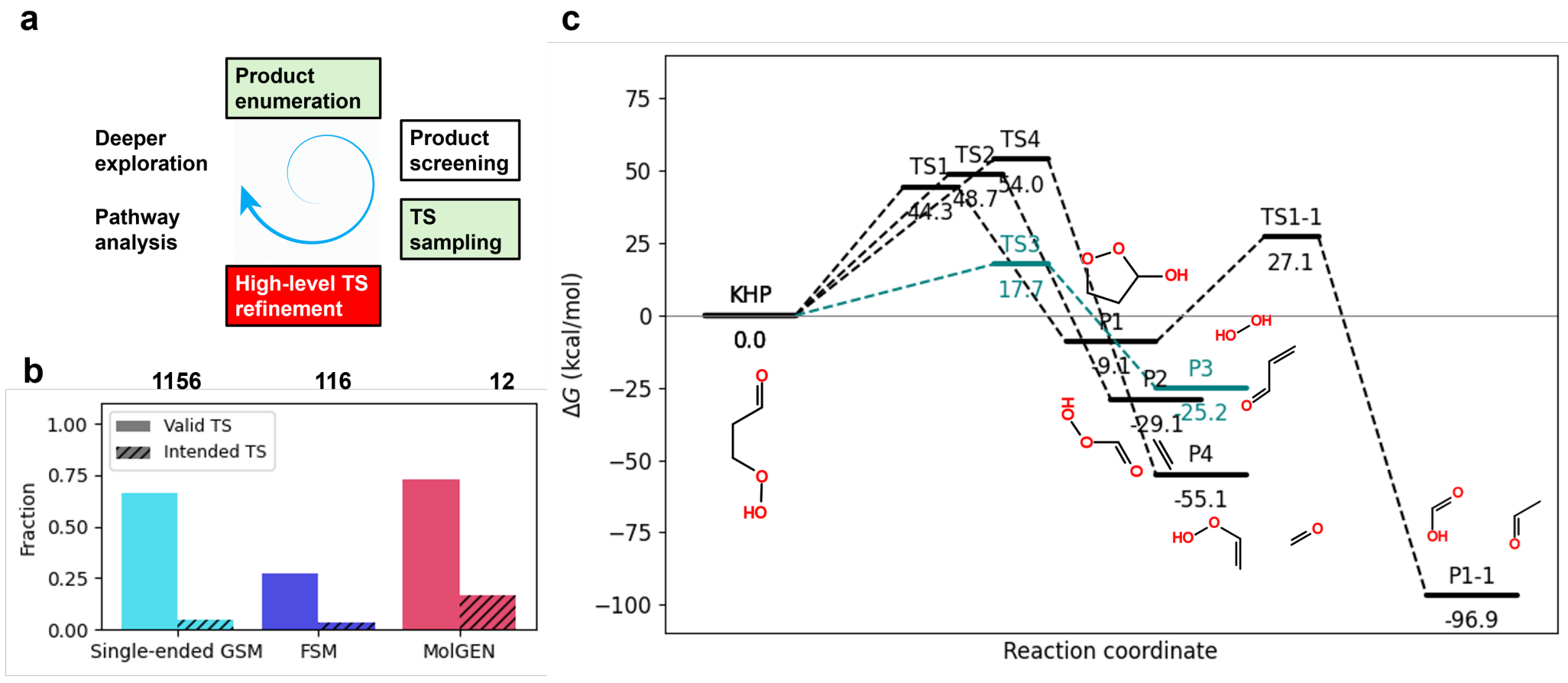

Fig. 4 a. Workflow of reaction network exploration. Product enumeration and TS sampling are each performed by a separate MolGEN model. Product screening is carried out using fixed, physically motivated criteria. **b** Fraction of valid and intended transition states (TS) obtained using three methods: MolGEN, single-ended growing string method (GSM), and freezing string method (FSM). The average number of high-level quantum chemical gradient descents per TS optimization is indicated above each bar. The number of high-level steps is significantly reduced by using MolGEN models for product and TS sampling. Values for single-ended GSM and FSM are adapted from Grambow [35]. **c** Representative decomposition pathways of KHP. All energies are computed at the ωB97X/6-31G(d) level of theory. Highlighted in green is the pathway with the lowest transition barrier, which is lower than the minimum barrier reported by YARP [36].

5. Most importantly, the paper overall lacks sufficient results to back up the claims made. While the comparison to other TS prediction models is nice, the dataset of reactions with only up to 7 atoms is really too limited to make claims about a general-purpose design or flow-matching as the future of molecular generation across chemistry. Similarly for the product predictions. Ideally, not just simulated datasets, but also experimentally verified datasets are included.

Author:

We thank the reviewer for pointing out this limitation and we agree that it is important to be precise about the scope of our conclusions. TS benchmarks are indeed restricted to relatively small molecules, primarily because high-level electronic-structure calculations for transition states remain computationally demanding. This is why many prior TS datasets (including Transition1x and RGD1) and models (including TSDiff, OA-ReactDiff, ReactOT) are also constructed from small organic reactions.

In the revised manuscript, we have softened our language wherever it might suggest that the present MolGEN model is already a fully general-purpose solution “across all of chemistry”. Our intention is instead to highlight two points: (i) on a standard small-molecule TS benchmark, flow-matching provides a competitive and more efficient alternative to diffusion-based TS generators; and (ii) the framework is general and, in principle, applicable to larger and more complex systems as related

data become available.

Concretely, we have made the following revisions:

1) We rewrite the abstract as

"Elucidating reaction mechanisms requires efficient generation of transition states (TSs), products, and complete reaction networks. While diffusion models and sequence-based generators accelerate TS and product sampling, respectively, current pipelines still depend on manual enumeration for at least one of these steps. Moreover, the stochastic dynamics underlying diffusion models can be inefficient and difficult to control. We show that flow matching, a deterministic ordinary differential equation formulation, can replace stochastic differential equation-based diffusion for reaction generation, and introduce MolGEN, a conditional flow-matching framework that learns an optimal-transport path mapping Gaussian priors to target chemical distributions. On benchmarks used by TSDiff, OA-ReactDiff, and ReactOT, MolGEN improves TS geometry accuracy and barrier-height prediction while enabling sub-second sampling. For product generation, it achieves competitive top-k accuracy and avoids mass/electron-balance violations common to sequence models. Crucially, MolGEN unifies TS and product sampling within a single backbone, enabling reaction network exploration driven by generative inference rather than templates or repeated quantum-chemistry TS searches. In a realistic test on the γ-ketohydroperoxide decomposition network, MolGEN yields more valid/intended TSs than string-based methods while drastically reducing the number of quantum-chemistry evaluations from 1,156 to 12. Furthermore, MolGEN uncovers a novel pathway with a barrier lower than the reported minimum, demonstrating an accurate and efficient framework for generative reaction-network exploration."

2) We changed the title of Section 2 from "A general-purpose conditional flow matching design" to "*A conditional flow matching design for reaction pathway generation*".

3) We now name the MolGEN-based reaction network solution "*an integrated generative model-based framework that substantially automates reaction network exploration*", instead of a self-contained solution to reaction network exploration.

4) We removed the last sentence of Section 2: "Ultimately, MolGEN demonstrates that flow matching is all that is needed for molecular generation."

5) We rewrite the last paragraph of the introduction as

"Here, we show that flow matching can fully replace diffusion-based formulations for reaction mechanism generation. We introduce MolGEN, a conditional flow-matching framework that generates both TSs and reaction products and, crucially, rolls out multi-step reaction networks using generative inference alone, without templates or hand-crafted rules. To our knowledge, MolGEN is the first end-to-end reaction-network generation framework powered solely by generative models for both TS and product sampling, enabling unified, automated exploration from reactants to complete networks. In Section 3.1, we show that MolGEN halves the TS geometry prediction error of the

state-of-the-art diffusion models while reducing sampling time to sub-second inference. Remarkably, it also uncovers alternative transition states with lower barrier heights than those reported in the reference data, underscoring its potential to reveal previously inaccessible mechanistic pathways. In Section 3.2, we evaluate MolGEN on reaction product generation from given reactants, where it attains top-k accuracies comparable to state-of-the-art sequence-based approaches while naturally respecting stoichiometric constraints. Together, these results show that a deterministic flow formulation provides an integrated solution for reaction-network rollout. In Section 3.3, we apply this framework to the decomposition network of γ-ketohydroperoxide (KHP). In this realistic benchmark, MolGEN achieves substantial computational efficiency gains over conventional string-based methods while maintaining high mechanistic fidelity, and it identifies a new decomposition pathway with a barrier lower than the reported minimum. Overall, the combination of high-quality generation, minimal computational cost, and unified TS and product sampling positions flow matching as a compelling paradigm for automated generative reaction network generation."

6. The demonstration of RN generation suffers from a lack of clear results, and on second inspection, are not very impressive, barely outperforming single-ended GSM according to fig. 2d. It is unclear whether the predictions are, or can be, experimentally verified. The procedure for RN generation also seems rather subjective, with a lot of intermediate filtering of TSs/products, making the claim of a self-contained and automated framework dubious.

Author:

We thank the referee for these comments. The referee raised concerns that (1) the RN generation seems barely outperforming single-ended GSM according to fig. 2d; (2) it is unclear whether the predictions can be experimentally verified; (3) the procedure for RN generation seems subjective, with intermediate filtering of products/TSs, making the claim of self-contained automated framework dubious.

In response to the 1st concern, we thank the reviewer for this observation and agree that the goal of the reaction-network (RN) experiment needs to be stated more clearly. The MolGEN model is trained on databases (Transition1x and RGD1) constructed using single-ended GSM at the DFT level. As a consequence, MolGEN is not expected to discover drastically new reaction pathways that are completely absent from the GSM-discovered pathways. Rather, the KHP example is designed as a proof-of-concept that a generative TS model + a generative product model, once trained, can (i) reproduce the GSM-accessible mechanistic landscape and (ii) substantially reduce the number of expensive quantum-chemical calculations required to assemble a reaction network.

In other words, for KHP the right question is not "Can MolGEN dramatically outperform GSM on coverage of known pathways?", since the training data itself is GSM-based, but instead:

1) Can MolGEN generate high-quality TS guesses in a single forward pass, so that fewer NEB/TS optimizations are needed?

2) Can it concentrate probability mass on mechanistically meaningful channels, improving the fraction of valid/intended TSs among candidates sent to high-level refinement?

Our results in Fig. 2d and the surrounding text show that MolGEN achieves comparable or slightly better intended-pathway coverage than single-ended GSM on KHP while requiring far fewer high-level optimizations per successful TS. Furthermore, the MolGEN RN framework managed to identify a new reaction pathway with a lower barrier than the previously reported minimum.

To make these points clearer, 1) we add the following comment in lines 328-329 of the revised manuscript:

"*This indicates that the MolGEN-based workflow substantially reduces the number of expensive quantum chemical calculations required to assemble a reaction network.*"

2) And we add the following comment in the caption of Figure 3b:

"*The number of high-level steps is significantly reduced by using MolGEN models for product and TS sampling.*"

3) We rewrite the abstract and introduction to highlight these points, as shown in the response to Q5.

In response to the 2nd concern, we thank the referee for raising this important point. In this work, our primary focus is on developing and validating the MolGEN framework at the level of quantum-chemical accuracy, so our evaluation is based on comparison to the DFT reference by Zhao et al. [1], rather than direct experiments. As for whether the predictions can be experimentally verified, we refer to the comment by Zhao et al. [1]:

"Unfortunately, the proliferation of such algorithms has not been paralleled by the availability of shared benchmark problems and performance statistics. In developing such benchmarks, two aspects of these methods are critical for assessing performance. **The first is whether a given method correctly identifies all relevant products and their low-barrier TSs under given reaction conditions. Any given method will find an array of reaction channels, but without a direct comparison against either detailed experimental data or other algorithms applied to the same problem the actual relevance of discovered pathways is unknown. To the best of our knowledge, a recent study by Grambow et al. [2] (combined with revised AFIR results later published by Maeda et al. [3]) represents the only head-to-head comparison of this kind, albeit only for three distinct families of methods and a single unimolecular degradation problem.** When it comes to assessing accuracy there are also the related questions of whether the lowest barrier transition state for a given reaction is discovered (e.g., a change of conformation can qualitatively modulate barriers and reaction mechanisms), whether the rank ordering of various channels is correct (e.g., a method with inaccurate absolute barriers that still reliably predicts the major product could still be useful), the level of theory of the final PES (e.g., choice of functional or use of a solvent model), whether the transition states are actually validated (e.g., using pathway sampling or an intrinsic reaction coordinate [IRC] calculation), and whether multi-step reactions yielding identical products are compared against single-step reaction channels. The second critical aspect for assessing performance is the computational cost of running the algorithm.

To address these gaps, a new series of benchmarks are reported. **The data generated as part of these benchmarks include competing TSs for each set of reactants, distinct TS conformers for each reaction, intrinsic reaction coordinate (IRC) characterizations, and general measures of computational cost** characterized using the updated version of the Yet Another Reaction Program (YARP) algorithms. "

[1] Zhao, Q., Savoie, B.M.: Algorithmic explorations of unimolecular and bimolecular reaction spaces. Angewandte Chemie 134(46), 202210693 (2022).

[2] C. A. Grambow, A. Jamal, Y. P. Li, W. H. Green, J. Zador, Y. V. Suleimanov, J. Am. Chem. Soc. 2018, 140, 1035.

[3] S. Maeda, Y. Harabuchi, J. Chem. Theory Comput. 2019, 15, 2111.

In response to the 3rd concern, we agree that the filtering logic should be made more explicit. The intermediate filtering steps in our RN rollout are not ad hoc manual interventions applied on a case-by-case basis, but fixed, physically motivated criteria that are applied algorithmically to all candidates. In the revised Methods, we provide a detailed description of all filters used.

"*Details of the reaction network exploration workflow*

Physically motivated criteria for product pruning: *The following criteria are either used or suggested to be useful for product pruning in this work*

1. *The product configuration must be at a local minimum, verified by vibrational analysis;*
2. *The product configuration can not be the same as the reactant according to adjacency matrix comparisons (This criterion is only relevant for the small-molecule reactions studied in this work and does not apply to larger molecules, for which distinct conformers sharing the same adjacency matrix become important);*
3. *Radical products are excluded from the list of allowed configurations for further reactions due to the typically high transition barriers. This is confirmed by Supplementary Section 3.3, where we list the Gibbs transition barrier of all the generated first-step KHP decomposition reactions. The three radical products all result from higher transition barriers (*92.703 kcal/mol, 72.119 kcal/mol, 70.842 kcal/mol*) than the majority of the generated reactions.*
4. *(Suggested) For KHP decomposition, small molecules can be removed from the list of allowed configurations to react again. This is not because MolGEN fails to predict the correct products, but because we are modeling a decomposition-type scenario in which volatile or low-boiling coproducts are effectively removed from the system as they form. In practice, this corresponds to steering the exploration away from recombination pathways (where small fragments recombine into larger molecules) and toward further decomposition, which is the mechanistic regime of interest for KHP. Physically, this is akin to simulating a process in which small molecules are continuously extracted (e.g. by gas flow), thereby driving decomposition forward rather than allowing back-reaction and recombination.*

Definition of "intended reactions" and "intended TS": *In reaction-network exploration, a TS is classified as an intended TS, and the corresponding reaction as an intended reaction, if, after geometry optimization, it connects the same reactant–product pair as the input reactant-product pair. The intended-TS metric therefore reflects (1) whether the specified reactant-product pair indeed corresponds to an elementary reaction and (2) the reliability of the TS-generation method.*

Conversely, unintended TSs remain valuable for reaction-network construction, as they reveal alternative reactions involving different reactant-product pairs."

In addition, to avoid overstating our level of automation, we have also softened our wording and now describe the MolGEN-based reaction network exploration framework as an *"integrated generative model-based framework that substantially automates reaction-network construction"*, while still relying on standard quantum-chemical refinement and programmatic filters, rather than as a completely self-contained solution.

7. Throughout the paper, references to the Methods and SI are missing, as well as proper explanations of various mechanisms used in this research. It would also be nice to include explanations about the model code, both in the SI and on Github.

Author:

We thank the reviewer for this helpful suggestion. In the revised manuscript, we now add cross-references to the Methods and the Supplementary Information wherever corresponding contents are invoked in the main text.

Regarding the model code, we now show the new Figure 1 in the GitHub README and add a subsection on model hyperparameters.

Minor comments: 8. Line 121: where does the velocity field ut(x) come from?

Author:

We thank the referee for pointing this out. To clear the definition up, we now change line 121 (line 129 of the revised manuscript) to

"$\boldsymbol{u}_t(\boldsymbol{x})$ *denotes the corresponding velocity field driving the transformation. Details of the optimal transport path, the corresponding velocity field, and the loss functions are given in the Methods.*"

9. Line 130: 'MolGen demonstrates that flow matching is all that is needed', statement is not backed up by the paper.

Author:

We agree that this statement is too strong, and as described in the response to Q5, we have removed this sentence.

10. Line 165: Characteristic chemical accuracy is 1.58 kcal/mol at 70 C. Why is this specific temperature relevant? Are the datasets for reactions at exactly 70 C, or why is this reference temperature used? Is there a way for the model to include different temperatures and other macroscopic variables in its predictions?

Author:

We apologize for the confusion and thank the reviewer for pointing this out. The number $1.58\ \mathrm{kcal/mol}$ at $70\ {}^{\circ}\mathrm{C}$ is not a property of the datasets themselves, but a way to provide physical intuition for barrier errors via transition-state theory.

In more detail, "*under transition-state theory, the rate constant scales as* $k \propto$ *exp*$(-\Delta G^{\ddagger}/RT)$. *An*

error $\delta\Delta G\ddagger$ *in the activation free energy translates into a multiplicative error of exp*$(\delta\Delta G\ddagger/RT)$ *to the reaction rate. A ten-fold error in rate corresponds to* $\delta\Delta G\ddagger = RT\textit{ln}10 \approx 2.303\ RT$. *At a representative synthetic temperature of* $70\ ^{\circ}\mathrm{C}$, *this error is* $2.303\ RT \approx 1.58\ \mathrm{kcal/mol}$." We chose $70\ ^{\circ}\mathrm{C}$ simply as a typical moderate reaction temperature to contextualize what a "chemically meaningful" barrier error means in terms of rate uncertainty. We now add this explanation in *Section Methods Subsection Evaluation*.

In response to the question "Is there a way for the model to include different temperatures and other macroscopic variables in its predictions?", in the current work, MolGEN is not conditioned on temperature or other macroscopic variables, because the available TS datasets (Transition1x, RGD1) do not provide barrier labels as a function of T, solvent, or pressure. However, the framework is readily extensible to such conditioning: additional inputs such as reaction temperature, solvent descriptors, or other macroscopic parameters could be concatenated to the molecular features or used as global conditioning variables in the flow.

11. Is the train/validate/test done on a single split, or is multi-fold training used?

Author:

We use a single, fixed train/validation/test split and reuse this split for all models. We now explicitly state this after introducing the Transition1x database, in line 152 of the revised manuscript.

12. It is unclear if the model is re-trained for the product-prediction task, or if the same model as for the TS prediction is used.

Author:

We thank the referee for pointing this out, a separate model is trained for the product-prediction task. We now explicitly point this out in the first paragraph of Section 3.2 by

"*Here, we demonstrate that MolGEN can also capture open-ended mappings with multiple outcomes, by training a separate MolGEN model to generate the product from a given reactant.*"

13. For the reaction network generation, why is the prediction of transition states needed at all? Why can you not go from reactants directly to a set of products?

Author:

We appreciate the opportunity to clarify this point. It is certainly possible to build models that map reactants directly to products without explicit transition states, and we agree that such "black-box" product predictors are useful for some tasks. Our goal in this work, however, is to go beyond product enumeration and to provide a mechanistic reaction network with kinetic information.

Explicit transition states play several roles in this context:

i) Kinetic relevance. TSs and their barrier heights are required to estimate rate constants via transition-state theory or related kinetic models. A network constructed only from reac-

tant–product pairs lacks the activation energies needed to distinguish fast vs. slow channels and therefore cannot readily support kinetic simulations.

ii) Microreversibility and consistency. By explicitly modeling TSs along both forward and reverse directions, we can enforce (or at least check) detailed-balance-like constraints and ensure that the network is thermodynamically and kinetically consistent. Pure reactant→product mappings do not provide this information.

iii) Mechanistic interpretability. Many applications, especially in catalysis and complex decomposition, require not only "what products are formed" but also how they are formed (e.g., radical vs ionic pathways, concerted vs stepwise). TS geometries and connecting paths are central to this mechanistic interpretation.

For these reasons, our reaction-network demonstration trains both a product generator and a TS generator.

14. Line 281: 'three radicals were excluded as reaction products.' However, if the model predictions are correct, and radicals are indeed produced, why leave them out from further reaction prediction? In that case, these would seem important to include in the network mechanism.

Author:

Excluding radicals from further exploration is a common choice by traditional methods, such as YARP, due to the fact that reactions with radical products tend to be endothermic and have high transition barriers. This is confirmed by Supplementary Section 3.3, where we list the Gibbs transition barrier of all the generated products of the first-step reaction. The three radical products all result from higher transition barriers (92.703 kcal/mol, 72.119 kcal/mol, 70.842 kcal/mol) than the majority of the generated reactions. To clear this up, we rewrite lines 316-317 of the revised manuscript as

"*Among the products of these intended reactions, three contained radicals, which tend to be endothermic and result from high transition barriers (refer to Supplementary Section 3.3), were excluded, leaving the remaining 11 products as reactants for subsequent exploration steps.*"

15. Line 380: Why is linear change in time a natural choice for probability paths? Line 386: Why would the final distribution $p_1(x|x_1)$ be a Gaussian?

Author:

We thank the reviewer for these questions regarding the choice of probability paths in our flow-matching formulation.

1) "Why is linear change in time a natural choice for probability paths?" In our construction, "linear change in time" refers to the fact that we interpolate linearly between the endpoints and use a simple, analytically tractable schedule for the variance. This choice is standard in the flow-matching and rectified-flow literature because it yields closed-form expressions for the conditional velocity

field. We do not claim that linear interpolation is the only possible or physically mandated choice; rather, it is a mathematically convenient path that has been empirically found to be robust for a wide range of generative tasks.

2) "Why would the final distribution $p_1(x|x_1)$ be a Gaussian?" In the manuscript we follow the usual conditional flow-matching formulation and define a Gaussian path $p_t(x|x_0, x_1)$ whose mean interpolates between the endpoints and whose variance shrinks as $t \to 1$. For $t < 1$, $p_t(x_t|x_1)$ is indeed Gaussian with finite variance. At $t = 1$, this distribution collapses to a Dirac-like distribution concentrated at x_1. The Gaussian form is used as a convenient family along the path, and the endpoint is recovered as the zero-variance limit. To make this clearer, we add the following sentence at the end of Section Methods Subsection (Optimal transport path):

"*At $t \to 1$, this distribution converges to a delta-like sharp Gaussian with zero variance.*"

Overall, our use of a linear, Gaussian path is consistent with established flow-matching practice and chosen for analytical tractability. At the same time, the method itself is not restricted to this specific choice, and alternative paths could in principle be explored without changing the high-level MolGEN framework.

16. The Github repository (github.com/tuoping/MolGEN) contains a lot of code that does not relate to the paper at all, and seems to be copied from various ML repositories (DeepMind/Alphafold, Meta/ChemNet, Microsoft)

Overall, I appreciate the interest of a flow-matching framework for molecule prediction, but more results and improved figures are strongly needed to properly assess the framework and warrant a publication in Nature Communications.

Author:

We thank the referee for their detailed inspection of our work and helpful suggestions. As summarized in the response to Q1, we make the following modifications in the revised manuscript to improve the presentation of our results:

1) To better illustrate the pipelines used in this work, both for TS generation and for product generation, we replot Figure 1, as shown in the response to Q1.

2) **For TS generation,** to improve clarity, we rearrange Table 1 and split the table into two parts, each trained on the same dataset.

3) **For TS generation,** we add a new Figure (Figure 2 in the revised manuscript) and compare the performance of various models, in response to Q2.

4) **For product generation,** we add a new Figure (Figure 3 in the revised manuscript) and compare the top-k step accuracy of MolGEN and FlowER on the Transition1x test set, in response to Q3.

5) **For reaction network exploration,** we add a new Table 2 to summarize the comparison between the performance of MolGEN and YARP on KHP decomposition reaction network exploration.

6) **For reaction network exploration,** we rewrite the abstract and introduction to highlight this part of the result.

Reviewer #3 (Remarks on code availability):

The code does contain a readme to install and run the training pipeline, but it lacks any explanation about the overall code structure and individual scripts. After looking through the code, I noticed that large parts of the repository contain code not related to the manuscript, but instead were seemingly copied over from various other machine-learning repositories (possibly AlphaFold, ChemNet, perhaps others). It is not necessarily bad to have copied code (and appropriate copyright claims were provided), but there is currently simply no way to properly assess the code related to the MolGen framework. The repository will need to be cleaned up, and documentation needs to be added.

Author:

We thank the referee for these helpful suggestions. We have cleaned the repository and removed all unused scripts. The only scripts containing a main function are now "train-Transition1x-equivariant.py" and "Transition1x-inference.py", and we provide detailed instructions on how to run them. In addition, we show the new Figure 1, and add a subsection titled "Model hyperparameters" to clarify the code structure.

We thank the referees for their examination of our revised manuscript and helpful suggestions.

In what follows, we address each of the points raised by the referees. The new revisions of the manuscript in response to the referees' comments is highlighted in red in the main text.

Reviewer #1 (Remarks to Author):

I have carefully examined the revised version of the manuscript entitled "Flow matching for reaction pathway generation" by Ping Tuo, Jiale Chen, and Ju Li.

The authors have appropriately addressed the comments raised during the first round of review. In particular, the revisions made in response to the other reviewers' suggestions have further strengthened the clarity and presentation of the work.

My overall assessment remains unchanged. I continue to consider this manuscript a significant and original contribution to the field, and I maintain my previous recommendation for publication in Nature Communications without further revision.

Reviewer #2 (Remarks to the Author):

- The authors said they changed the name of the loss to modified Jensen-shannon, but I still see "KL div" throughout the paper. Now there is an inconsistency. Please fix this.

If they fix this, I think you had thoughtful responses to my questions. I would support publication.

Author:

We thank the referee for the careful reading and apologize for this oversight. We have corrected all instances of "KL div" to "JS div" in the main text.

Reviewer #3 (Remarks to the Author):

The authors carefully and appropriately addressed the comments I had, and the presentation of the results and context of this work has much improved.

In my opinion, the figures and some results still need a careful look at:

Fig 1: A schematic overview of the principles of flow networks would help the reader better understand how they are different from diffusion models, and what the two loss functions physically/computationally represent. For fig 1c, the text denoting the various messages and their variables is really too small to read. Additionally, I would like to see which architectural choices have been made regarding the MPNN. The Methods section on this is quite extensive, but it would be useful to the reader to see some of this back in the manuscript.

Author:

We thank the referee for these constructive suggestions. We agree that the original Fig. 1 did not sufficiently emphasize the conceptual difference of flow matching from diffusion models, and that the architectural summary shown in the main text could be clearer. In the revised manuscript, we have therefore:

(1) revised Fig. 1a to include a more explicit schematic of the flow matching path, showing the optimal-transport nature of the path;

(2) enlarged the text and labels in Fig. 1c to improve readability; and

(3) We expanded the caption of Fig. 1 to clarify how the loss functions relate to the schematic and to summarize the key architectural choices of the MPNN, which were previously described primarily in the Methods.

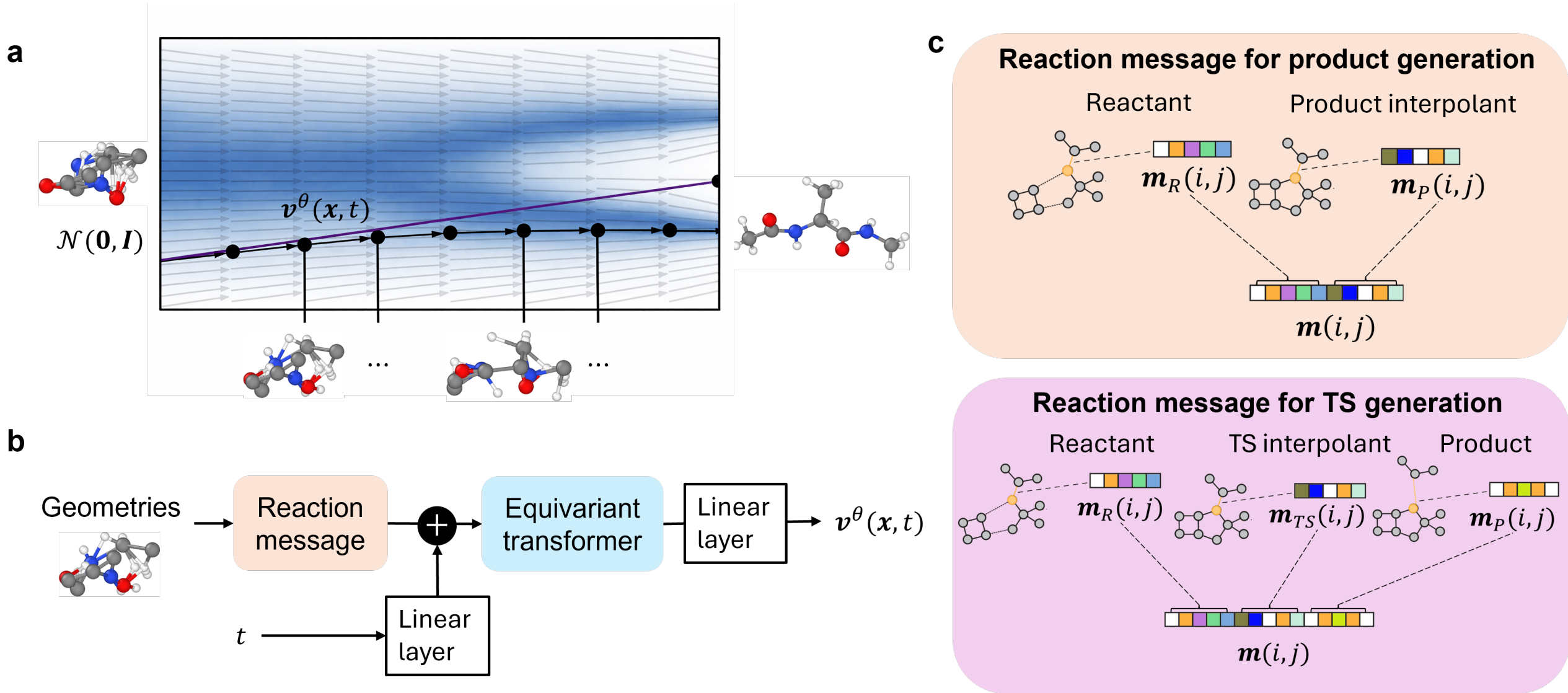

Figure 1: **Overview of MolGEN. a.** Schematic illustration of the flow matching workflow. The reactant (or reactant and product) geometry and a randomly initialized product (or TS) geometry are fed into a flow network, which predicts a velocity field ("flow"). This field is then applied iteratively to the random geometry, transforming it into the target product (or TS). The model is trained with the FM loss to match the target flow and with the JS loss to match the probability path, illustrated by the blue shading. **b.** A schematic of the MPNN flow model. The workflow takes the input geometries (e.g., the reactant and the product interpolant) and encodes them into a reaction message via Clebsch–Gordan contractions of SE(3)-equivariant tensor features constructed from spherical harmonics and radial basis functions [21]. In parallel, the scalar integration time t is passed through a linear layer to produce a time embedding, which is added to the reaction messages and then processed by an SE(3)-equivariant transformer [22], and a final linear layer outputs the flow v_t, which iteratively updates the product interpolant. **c.** Illustration of the reaction messages. For product generation, the molecular messages of the reactant $\boldsymbol{m}_R(i,j)$ is computed from its geometry, while the product message $\boldsymbol{m}_P(i,j)$ is obtained from the flow-matching interpolants. The overall reaction message, $\boldsymbol{m}(i,j)$, is constructed by concatenating $\boldsymbol{m}_R(i,j)$ and $\boldsymbol{m}_P(i,j)$. The reaction message for TS generation is constructed similarly by concatenating $\boldsymbol{m}_R(i,j)$ and $\boldsymbol{m}_P(i,j)$ constructed from the corresponding geometries, and $\boldsymbol{m}_{\mathrm{TS}}(i,j)$ constructed from the TS interpolants.

Fig 2. The cumulative probability of barrier height error is a confusing way of showing the (relative)

model performance. Why not just show error histograms, or predicted-vs-true plots for every model? This would allow the reader to more easily assess the relative performance and also the performance for different barrier heights.

Author:

We agree that an error histogram provides a more intuitive comparison of relative performance. Accordingly, we have revised Fig. 2 to include an error histogram.

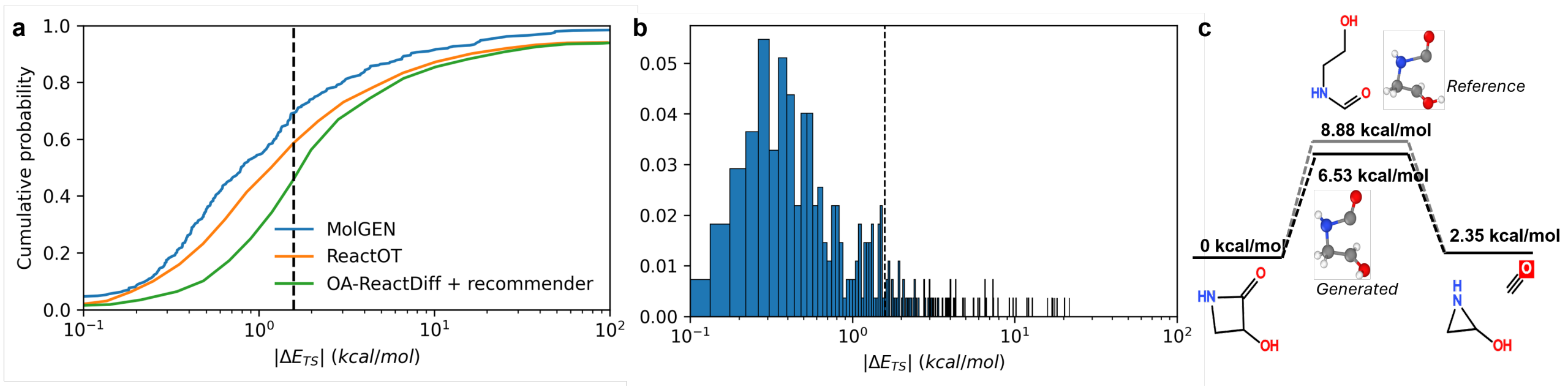

Figure 2: **a.** Cumulative probability of the barrier height error. The ReactOT and OA-ReactDiff results are reproduced from Reference [28] and [17], respectively. The dashed line marks the threshold of chemical accuracy. **b.** Histogram of the barrier height error. **c.** An example transition state (TS) generated by MolGEN, with a lower barrier than the reference.

Fig 3. a) The Graph2SMILES benchmark mentioned in the previous version is now missing from both the text and the figures. Furthermore, fig 3b and 3c are not mentioned in the text. For 3b I'm not sure why the distribution of product relaxation energy change is relevant. Is this relaxation done after MolGen, or is it performed by MolGen? And if done after MolGen, then why in Fig 2b are the Reference and Generated TS not the same. They look like a non-relaxed and relaxed counterpart (only a slight difference in geometry).

Author:

We thank the referee for such careful reading and insightful suggestions. The referee raised 3 questions: 1) The Graph2SMILES benchmark mentioned in the previous version is now missing from both the text and the figures; 2) Why is the distribution of product relaxation energy change relevant? 3) Why in Fig. 2b are the Reference and Generated TS not the same? As they look like a non-relaxed and relaxed counterpart with only a slight difference in geometry. In addition, the referee pointed out that Fig. 3b and 3c are not mentioned in the text.

In response to the 1st question, the referee previously raised concerns about the lack of a direct comparison between MolGEN and FlowER/Graph2SMILES in the figure. We therefore attempted to reproduce results using both FlowER and Graph2SMILES. The FlowER results are now included in Fig. 3a. However, we were unable to run Graph2SMILES because of deprecated version-compatibility issues, and thus only FlowER was retained for benchmark.

In response to the second question, Fig. 2 presents the product prediction performance in two panels. Figure 2a reports the top-k accuracy, which evaluates whether the adjacency matrix of the predicted product is correct. Figure 2b reports the energy deviation of the predicted geometry from the energy minimum, which assesses the absolute accuracy of the predicted coordinates and is widely used to evaluate the quality of geometries generated by generative models. To clarify this point, we now add the following explanation in line 215:

"Figure 3b presents energy deviation of the predicted geometry from the local minimum, which assesses the absolute accuracy of the predicted coordinates. A representative example of the predicted product and the corresponding reference is shown in Figure 3c."

In response to the third question, both the reference and generated TS structures in Fig. 2c are relaxed to local minima. Although they appear nearly identical, differing primarily in the orientation of one $O-H$ bond (indicated by the red arrow in the figure below), their energy difference is $2.35\ \mathrm{kcal/mol}$, exceeding the threshold for chemical accuracy. They thus correspond to different conformers of the same molecular formula. This example illustrates the pronounced sensitivity of molecular energy to subtle geometric variations and the resulting challenge of achieving accurate predictions, especially when assessed by ΔE.

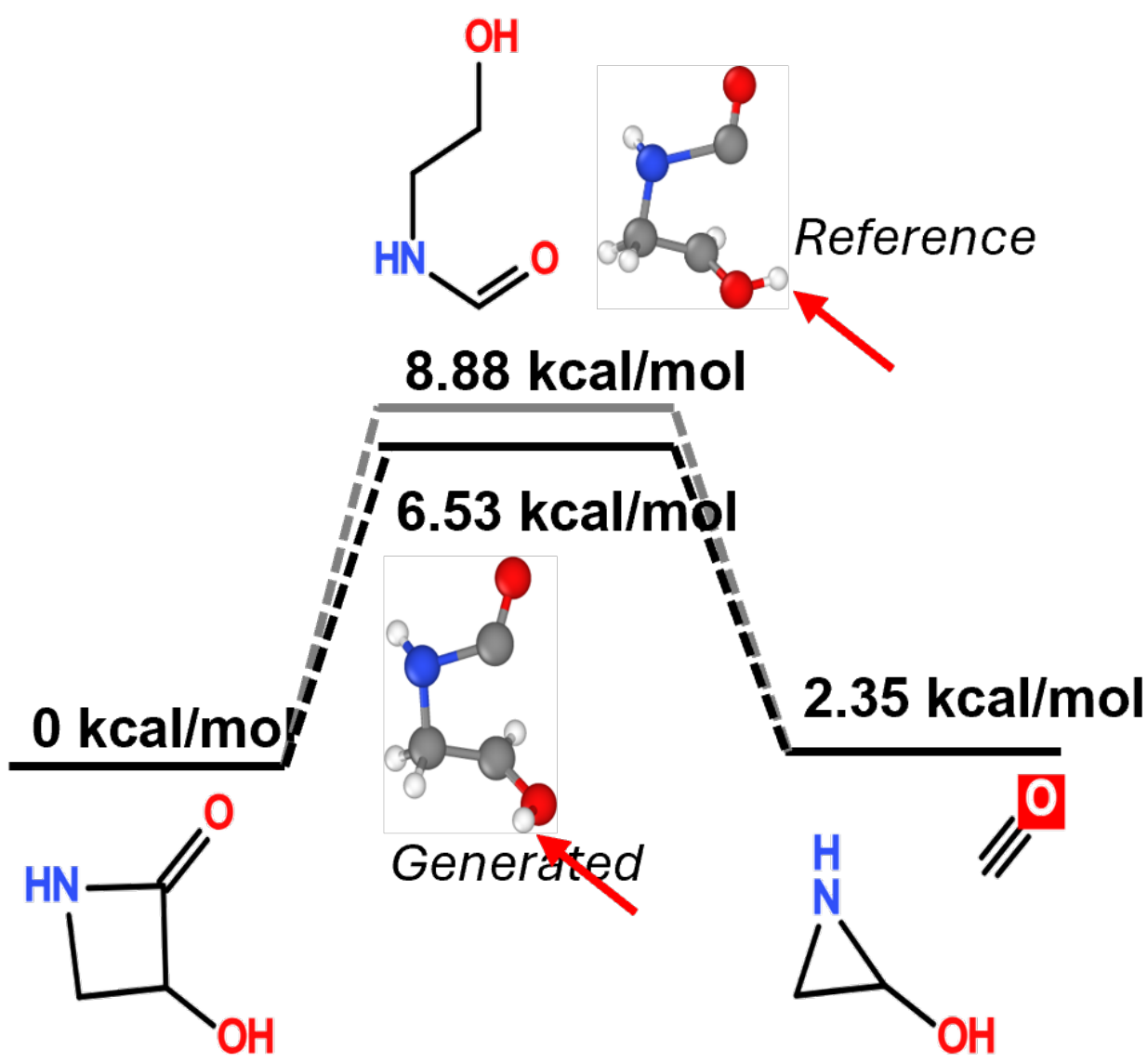

Section 3.3, fig 4. Here, I'm still missing a clear visualization of which products are screened out. There is text in the Methods describing the procedure, but I'm interested to see which exact products are generated and filtered at every step.

Author:

We thank the referee for pointing out that the manuscript seems to lacked a clear visualization of which products were screened out. In response, we have listed all generated products in the Supplementary Information, together with the key features used in the screening process.

Lastly, for a framework focussed on speed vs diffusion methods, I am still missing explicit results on their speed comparison, and how speed and accuracy scale for both flow and diffusion models.

Author:

We thank the referee for pointing out that an explicit speed comparison between flow and diffusion models is missing. In Section 3.1, we already include a paragraph on inference time, where we show the accuracy scaling of MolGEN with the number of function evaluations (nfe) required for converged sampling accuracy. The accuracy of MolGEN converges at nfe=10. To make the comparison between flow and diffusion models more explicit, we now add the following statement in lines 241–242.

"This stands in stark contrast to diffusion models, as TSDiff requires 5000 nfe for sampling [16], and OA-ReactDiff requires $\gtrsim 800$ nfe [17]."
